# Diagnostic performance of various liquid biopsy methods in detecting colorectal cancer: A meta‐analysis

**DOI:** 10.1002/cam4.3276

**Published:** 2020-07-06

**Authors:** Yuzhou Zhu, Tinghan Yang, Qingbin Wu, Xuyang Yang, Jianqi Hao, Xiangbing Deng, Shuo Yang, Chaoyang Gu, Ziqiang Wang

**Affiliations:** ^1^ Department of Gastrointestinal Surgery West China Hospital Sichuan University Chengdu Sichuan Province China; ^2^ West China Hospital Sichuan University Chengdu Sichuan Province China

**Keywords:** cell‐free DNA, colorectal cancer, CTCs, exosomes, liquid biopsy

## Abstract

Liquid biopsy is a promising method in detecting colorectal cancer (CRC). However, previous meta‐analyses only focused on the diagnostic performance of cell‐free DNA (cfDNA). Therefore, we firstly evaluated the overall performance of all liquid biopsy methods. The pooled sensitivities, specificities, diagnostic odds ratios, and area under curve (AUC) of summary receiver operating characteristic curve for all liquid biopsy methods, exosomes, circulating tumor cells (CTCs), and cfDNA were calculated, respectively. A total of 62 articles involving 18 739 individuals were included. Fifty‐one articles were about cfDNA, five articles were about CTCs, and six articles were about exosomes. The overall performance of all liquid biopsy methods had a pooled sensitivity, specificity, and AUC of 0.77 (95% confidence interval [CI] 0.76‐0.78), 0.89 (95% CI 0.88‐0.90), and 0.9004, respectively. The sensitivities were 0.82 (95% CI 0.79‐0.85), 0.76 (95% CI 0.72‐0.80), and 0.76 (95% CI 0.75‐0.77) for CTCs, exosomes, and cfDNA, respectively. The specificities were 0.97 (95% CI95% CI 0.95‐0.99), 0.92 (95% CI 0.89‐0.94), and 0.88 (95% CI 0.87‐0.89) for CTCs, exosomes, and cfDNA, respectively. The AUC were 0.9772, 0.9037, and 0.8963 for CTCs, exosomes, and cfDNA, respectively. The overall performance of all liquid biopsy methods had great diagnostic value in detecting CRC, regardless of subtypes. Among all liquid biopsy methods, CTCs showed the best diagnostic performance.

## Graphical abstract

The area under curve was 0.9772, 0.9037, 0.8963, and 0.9004 for circulating tumor cells (CTCs), exosomes, cell‐free DNA, and overall performance of all liquid biopsy methods in detecting colorectal cancer, respectively. The liquid biopsy had great diagnostic value in detecting CRC, regardless of subtypes. Among all liquid biopsy methods, CTCs showed the best diagnostic performance.

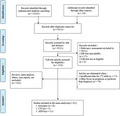

## 1 INTRODUCTION

Colorectal cancer (CRC), characterized by its high lethality and morbidity, is the third most common cancer in the world.^1^ Data from the US Preventive Services Working Group indicate that if CRC could be diagnosed earlier, about 60% of CRC deaths could be averted, and the average 5‐year survival rate could be raised from 46% to 73%.^2^ Therefore, early detection of CRC is the key to reduce mortality.^3^

Presently, colonoscopy is the gold standard for the diagnosis of CRC,^4^, ^5^ which decreases mortality by 68% and incidence by 69%.^6^ However, the benefits of colonoscopy are not without a price, as it is invasive, with undesirable compliance and sometimes harmful to the patients.^5^ Carcinoembryonic antigen (CEA) and carbohydrate antigen 19‐9 (CA 19‐9) are blood‐based markers in current clinical use, mainly for monitoring the response to treatment and progression of CRC. However, owing to low sensitivity and specificity, CEA and CA 19‐9 have limited value in CRC detection.^7^

In recent years, liquid biopsy is regarded as a method for cancer detection. Several methods have been used as liquid biopsy to detect biomolecules or cells in blood released from tumors, including cell‐free DNA (cfDNA) (nucleic acid chains derived from various cell types, including but not limited to tumor cells),^8^ circulating tumor cell (CTCs) (cells fall off from primary cancer into blood or lymphatic vessels^9^), and exosomes (vesicles secreted by cancer cells, with a diameter between 30 nm and 100 nm^10^). Those methods are mini‐invasive and could provide a representative sample of the whole tumor, which eliminates tumor heterogeneity.^11^ In the last decades, the diagnostic value of various liquid biopsy methods in detecting CRC has been extensively studied. However, all researches only evaluated diagnostic performance in single liquid biopsy method (cfDNA, CTCs, or exosomes). Currently, it is still under debate what the best liquid biopsy method is and what is the overall performance of all liquid biopsy methods. In the present study, we performed a meta‐analysis to evaluate the diagnostic performance of CTCs, exosomes, and cfDNA and the overall performance of those methods in detecting CRC.

## 2 MATERIALS AND METHODS

### 2.1 Search strategy

Two independent reviewers performed a systemic search on PubMed, EMBASE, Medline, and Web of science for eligible studies from 2002 to August 2019. The search strategy is shown in Table S1.

### 2.2 Inclusion and exclusion criteria

Potential articles were appraised by the reviewers independently based on the inclusion and exclusion criteria mentioned below. Any discrepancy was adjudicated by the third reviewer. The screening process was in accordance with the Preferred Reporting Items for Systematic Reviews and Meta—Analyses recommendation.^12^

Inclusion criteria: (a) human studies; (b) diagnostic studies reporting the absolute numbers of true‐positive (TP), false‐positive (FP), false‐negative (FN), and true‐negative (TN); and (c) the total number of patients and control group should be more than 20.

Exclusion criteria: (a) animal studies; (b) studies without sufficient data to build a 2 × 2 contingency table; (c) prognostic studies; (d) article types were systematic reviews, meta‐analysis, case‐reports, and letters or comments.

### 2.3 Quality assessment and data extraction

Two reviewers evaluated the quality of each eligible article according to Quality Assessment of Diagnostic Accuracy Studies‐2 (QUADAS‐2) ^13^ and extracted the following data from each included article: author, year of publication, sample size, country, control type, absolute numbers of TP, FP, FN, TN, measuring object, and assay methods. Sensitivity and specificity were then calculated. If more than one set was available in the same article, the set of data (TP, FP, FN, and TN) with highest area under curve (AUC) value was extracted.

### 2.4 Statistical analysis

Circulating tumor cells, exosomes, and cfDNA were the three major subtypes of liquid biopsy. Assay methods of cfDNA included measuring cfDNA level (CFD level), DNA integrity, and methylation of DNA. Pooled sensitivities (95% confidence interval [CI]), pooled specificities (95% CI), pooled positive likelihood ratio (PLR) (95% CI), pooled negative likelihood ratio (NLR) (95% CI), and pooled diagnostic odds ratio (DOR) for the overall performance of all liquid biopsy methods, CTCs, exosomes, and cfDNA were calculated using the bivariate model.^14^ These data were shown in both tabular form and in forest plots.

We used Spearman's correlation coefficient to evaluate the threshold effect and to assess heterogeneity among researches.^15^, ^16^, ^17^ A *P* value less than .05 implied significant threshold effect with a negative correlation between specificity and sensitivity.^18^ We also adopted Higgins *I* ^2^ statistic to measure heterogeneity among researches. If *I* ^2^ was more than 50%, which demonstrated substantial heterogeneity,^15^, ^19^, ^20^, ^21^ random‐effect model was adopted to integrate the data.^20^, ^22^, ^23^, ^24^ Publication bias was evaluated by Deeks’ funnel plots asymmetry test.^25^ A *P* value less than .05 suggested statistically significant publication bias.^25^ All statistical analyses were completed using Stata 14.0 software (Stata Corporation) and Meta‐disc 1.4 software (XI Cochrane Colloquium)^26^, ^27^

## 3 RESULTS

### 3.1 Study selection and description

**FIGURE 1:**
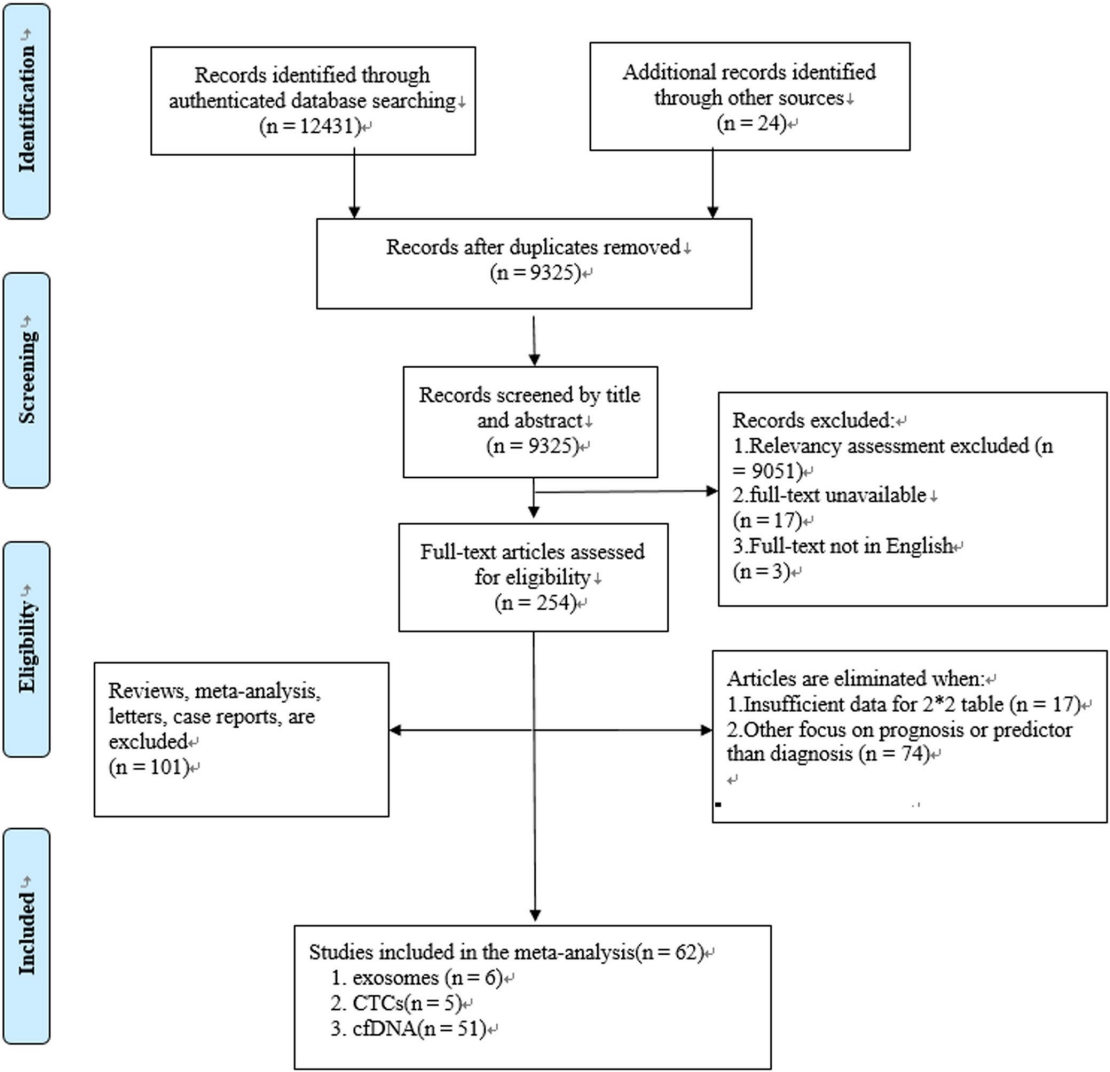
Flow diagram according to Preferred Reporting Items for Systematic Reviews and Meta—Analyses recommendation

The initial search yielded 12 455 articles. After screening titles and abstracts, and removing duplications, the full text of 256 articles was reviewed. Finally, 62 articles involving 18 739 individuals were included^28^, ^29^, ^30^, ^31^, ^32^, ^33^, ^34^, ^35^, ^36^, ^37^, ^38^, ^39^, ^40^, ^41^, ^42^, ^43^, ^44^, ^45^, ^46^, ^47^, ^48^, ^49^, ^50^, ^51^, ^52^, ^53^, ^54^, ^55^, ^56^, ^57^, ^58^, ^59^, ^60^, ^61^, ^62^, ^63^, ^64^, ^65^, ^66^, ^67^, ^68^, ^69^, ^70^, ^71^, ^72^, ^73^, ^74^, ^75^, ^76^, ^77^, ^78^, ^79^, ^80^, ^81^, ^82^, ^83^, ^84^, ^85^, ^86^, ^87^, ^88^, ^89^:51 articles were on cfDNA,^28^, ^29^, ^30^, ^31^, ^32^, ^33^, ^34^, ^35^, ^36^, ^37^, ^38^, ^39^, ^40^, ^41^, ^42^, ^43^, ^44^, ^45^, ^46^, ^47^, ^48^, ^49^, ^50^, ^51^, ^52^, ^53^, ^54^, ^55^, ^56^, ^57^, ^58^, ^59^, ^60^, ^61^, ^62^, ^63^, ^64^, ^65^, ^66^, ^67^, ^68^, ^69^, ^70^, ^71^, ^72^, ^73^, ^74^, ^75^, ^81^, ^82^, ^83^ five articles were on CTCs,^76^, ^77^, ^78^, ^79^, ^80^ and six articles were on exosomes.^84^, ^85^, ^86^, ^87^, ^88^, ^89^ The flow chart is summarized in Figure 1

Cell‐free DNA level, DNA integrity, and DNA methylation were the three different assay methods for cfDNA. Among the 51 studies on cfDNA,^28^, ^29^, ^30^, ^31^, ^32^, ^33^, ^34^, ^35^, ^36^, ^37^, ^38^, ^39^, ^40^, ^41^, ^42^, ^43^, ^44^, ^45^, ^46^, ^47^, ^48^, ^49^, ^50^, ^51^, ^52^, ^53^, ^54^, ^55^, ^56^, ^57^, ^58^, ^59^, ^60^, ^61^, ^62^, ^63^, ^64^, ^65^, ^66^, ^67^, ^68^, ^69^, ^70^, ^71^, ^72^, ^73^, ^74^, ^75^, ^81^, ^82^, ^83^ 11 measured CFD level,^28^, ^29^, ^30^, ^31^, ^32^, ^33^, ^34^, ^35^, ^57^, ^58^, ^60^ four measured DNA integrity,^57^, ^58^, ^59^, ^60^ and 36 measured DNA methylation.^36^, ^37^, ^38^, ^39^, ^40^, ^41^, ^42^, ^43^, ^44^, ^45^, ^46^, ^47^, ^48^, ^49^, ^50^, ^51^, ^52^, ^53^, ^54^, ^55^, ^56^, ^61^, ^62^, ^63^, ^64^, ^65^, ^66^, ^67^, ^68^, ^69^, ^70^, ^71^, ^72^, ^73^, ^74^, ^75^ Three articles measured both DNA integrity and CFD level.^57^, ^58^, ^60^ For DNA methylation, 21 focused on the methylation of SEPT9,^36^, ^37^, ^38^, ^39^, ^40^, ^41^, ^42^, ^43^, ^44^, ^45^, ^46^, ^47^, ^48^, ^49^, ^50^, ^51^, ^52^, ^53^, ^54^, ^55^, ^56^ and 15 were on the methylation of other sites.^61^, ^62^, ^63^, ^64^, ^65^, ^66^, ^67^, ^68^, ^69^, ^70^, ^71^, ^72^, ^73^, ^74^, ^75^ Basic characteristics of all 62 included studies are shown in Table S2.

### 3.2 Quality assessment

Methodology quality of 62 included studies was evaluated by QUADAS‐2 quality assessment tool and shown in Figure S1. Study quality was generally acceptable, varying from moderate to high.

### 3.3 Heterogeneity

Spearman's correlation coefficient was used to evaluate the threshold effect among researches. The *P* value was .567, revealing that no significant threshold effect existed. DOR was an appropriate balance between sensitivity and specificity, and we used Higgins *I* ^2^ of DOR to evaluate heterogeneity among studies. *I* ^2^ of DOR for all liquid biopsy methods was 78.1%.

### 3.4 Diagnostic performance for all liquid biopsy methods in detecting CRC

*I* ^2^ more than 50% showed that significant heterogeneity existed among studies; therefore, random‐effect model was adopted to pool data. The pooled sensitivity, specificity, PLR, NLR, and DOR for overall performance of all liquid biopsy methods in detecting CRC were 0.77 (95% CI 0.76‐0.78), 0.89 (95% CI 0.88‐0.90), 7.37 (95% CI 6.17‐8.81), 0.27 (95% CI 0.24‐0.31), and 30.28 (95% CI 23.82‐38.50), respectively. Summary receiver operating characteristic curve (SROC) was drawn and liquid biopsy yielded an AUC of 0.9004. The results are shown in Table 1 and Figure S2.

**TABLE 1:** Summary of subgroup analysis for liquid biopsy in the diagnosis of colorectal cancer

	Sensitivity (95% CI)	Specificity (95% CI)	PLR (95% CI)	NLR (95% CI)	DOR (95% CI)	AUC for SROC
*All liquid biopsy methods*	0.77(0.76‐0.78)	0.89(0.88‐0.90)	7.37(6.17‐8.81)	0.27(0.24‐0.31)	30.28(23.82‐38.50)	0.9004
CTCs	0.82(0.79‐0.85)	0.97(0.95‐0.99)	23.80(13.41‐42.24)	0.20(0.10‐0.37)	159.99(72.38‐353.67)	0.9772
Exosomes	0.76(0.72‐0.80)	0.92(0.89‐0.94)	7.17(4.00‐12.87)	0.24(0.16‐0.36)	27.67(12.94‐59.15)	0.9037
Overall cfDNA	0.76(0.75‐0.77)	0.88(0.87‐0.89)	6.20(5.17‐7.45)	0.28(0.25‐0.33)	23.98(18.74‐30.69)	0.8963
CFD level	0.77(0.74‐0.80)	0.92(0.90‐0.94)	8.85(4.04‐19.36)	0.29(0.20‐0.43)	33.83(12.93‐88.47)	0.8838
DNA integrity	0.71(0.66‐0.76)	0.94(0.91‐0.97)	8.95(4.36‐18.39)	0.32(0.21‐0.47)	31.40(11.87‐83.10)	0.9187
DNA methylation	0.76(0.75‐0.77)	0.88(0.87‐0.89)	6.20(5.17‐7.45)	0.28(0.25‐0.33)	23.98(18.74‐30.69)	0.8963
Methylation of SEPT9	0.74(0.72‐0.76)	0.87(0.86‐0.88)	6.70(5.34‐8.39)	0.31(0.26‐0.35)	24.44(17.67‐33.82)	0.8976
Methylation of other sites	0.78(0.76‐0.80)	0.89(0.88‐0.90)	5.54(4.02‐7.64)	0.27(0.20‐0.36)	22.58(15.09‐33.78)	0.8940

Abbreviations: AUC, area under curve; NLR, negative likelihood ratio; PLR, positive likelihood ratio.

### 3.5 Diagnostic performance for CTCs in detecting CRC

Five of all included articles were on CTCs. The pooled sensitivity, specificity, PLR, NLR, and DOR were 0.82 (95% CI 0.79‐0.85), 0.97 (95% CI 0.95‐0.99), 23.80 (95% CI 13.41‐42.24), 0.20 (95% CI 0.10‐0.37), and 159.99 (95% CI 72.38‐353.67), respectively. The AUC was 0.9772 (Table 1; Figure S3).

### 3.6 Diagnostic performance for exosomes in detecting CRC

Six articles were on exosomes.^84^, ^85^, ^86^, ^87^, ^88^, ^89^ The pooled sensitivity, specificity, PLR, NLR, and DOR were 0.76 (95% CI 0.72‐0.80), 0.92 (95% CI 0.89‐0.94), 7.17 (95% CI 4.00‐12.87), 0.24 (95% CI 0.16‐0.36), and 27.67 (95% CI 12.94‐59.15), respectively. The AUC was 0.9037. (Table 1; Figure S4).

### 3.7 Diagnostic performance for cfDNA in detecting CRC

After pooling 51 studies on cfDNA,^28^, ^29^, ^30^, ^31^, ^32^, ^33^, ^34^, ^35^, ^36^, ^37^, ^38^, ^39^, ^40^, ^41^, ^42^, ^43^, ^44^, ^45^, ^46^, ^47^, ^48^, ^49^, ^50^, ^51^, ^52^, ^53^, ^54^, ^55^, ^56^, ^57^, ^58^, ^59^, ^60^, ^61^, ^62^, ^63^, ^64^, ^65^, ^66^, ^67^, ^68^, ^69^, ^70^, ^71^, ^72^, ^73^, ^74^, ^75^, ^81^, ^82^, ^83^ the pooled sensitivity, specificity, PLR, NLR, and DOR for cfDNA in detecting CRC were 0.76 (95% CI 0.75‐0.77), 0.88 (95% CI 0.87‐0.89), 6.20 (95% CI 5.17‐7.45), 0.28 (95% CI 0.25‐0.33), and 23.98(95% CI 18.74‐30.69), respectively. Cell‐free DNA yielded an AUC of 0.8963 (Table 1; Figure S5).

Cell‐free DNA level, DNA integrity, and DNA methylation were the three assay methods for cfDNA. Subgroup analysis found that the pooled sensitivity, specificity, PLR, NLR, DOR, and AUC were 0.77 (95% CI 0.74‐0.80), 0.92 (95% CI 0.90‐0.94), 8.85 (95% CI 4.04‐19.36), 0.29 (95% CI 0.20‐0.43), 33.83 (95% CI 12.93‐88.47), and 0.8838 for CFD level group (Table 1; Figure S6), 0.71 (95% CI 0.66‐0.76), 0.94 (95% CI 0.91‐0.97), 8.95 (95% CI 4.36‐18.39), 0.32 (95% CI 0.21‐0.47), 31.40 (95% CI 11.87‐83.10), 0.9187 for DNA integrity group (Table 1; Figure S7), and 0.76 (95% CI 0.75‐0.77), 0.88 (95% CI 0.87‐0.89), 6.20 (95% CI 5.17‐7.45), 0.28 (95% CI 0.25‐0.33), 23.98 (95% CI 18.74‐30.69), 0.8963 for DNA methylation group (Table 1; Figure S8).

For DNA methylation, 21 articles were about SEPT9,^36^, ^37^, ^38^, ^39^, ^40^, ^41^, ^42^, ^43^, ^44^, ^45^, ^46^, ^47^, ^48^, ^49^, ^50^, ^51^, ^52^, ^53^, ^54^, ^55^, ^56^ with pooled sensitivity, specificity, PLR, NLR, DOR, and AUC of 0.74 (95% CI 0.72‐0.76), 0.87 (95% CI 0.86‐0.88), 6.70 (95% CI 5.34‐8.39), 0.31 (95% CI 0.26‐0.35), 24.44 (95% CI 17.67‐33.82), and 0.8976, respectively (Table 1; Figure S9). Fifteen articles were about the other methylation sites,^61^, ^62^, ^63^, ^64^, ^65^, ^66^, ^67^, ^68^, ^69^, ^70^, ^71^, ^72^, ^73^, ^74^, ^75^ with pooled sensitivity, specificity, PLR, NLR, DOR, and AUC of 0.78 (95% CI 0.76‐0.80), 0.89 (95% CI 0.88‐0.90), 5.54 (95% CI 4.02‐7.64), 0.27 (95% CI 0.20‐0.36), 22.58 (95% CI 15.09‐33.78), and 0.8940, respectively (Table 1; Figure S10).

### 3.8 Publication bias

Publication bias was assessed by the *P* value of Deeks' funnel plot asymmetry test. The *p* value was 0.94, revealing that publication bias was not significant. The result of Deeks’ funnel plot asymmetry test is shown in Figure S11.

## 4 DISCUSSION

This meta‐analysis included 62 articles on liquid biopsy involving 18 739 individuals; the pooled sensitivity and specificity for all liquid biopsy methods were 0.77 (95% CI 0.76‐0.78) and 0.89 (95% CI 0.88‐0.90), respectively. The AUC of SROC for all liquid biopsy methods was 0.9004 and revealed high diagnostic value, suggesting liquid biopsy was a powerful diagnostic tool for CRC.

A series of tumor markers have been associated with CRC, particularly CEA. However, all these markers have a poor diagnostic performance in detecting CRC because of significant overlap with benign disease and low sensitivity for early stage disease.^90^ A meta‐analysis concluded that the pooled sensitivity for CEA in detecting CRC was only 46% (95% CI 0.45‐0.47).^90^ No other tumor marker have a higher diagnostic sensitivity, including CA 19‐9 (pooled sensitivity 0.30, 95% CI 0.28‐0.32). Furthermore, there is also limitation in the specificity of CEA, which was reported to be 89% (95% CI 0.88‐0.92).^90^ Gastritis, liver disease, diverticulitis, peptic ulcer disease, diabetes, and any acute or chronic inflammatory state could all contribute to an elevated CEA level. Moreover, CEA levels are significantly higher in cigarette smokers than in nonsmokers.^91^ Compared with them, liquid biopsy has the same degree of invasion to patients, but a much better diagnostic performance (pooled sensitivity 0.77, 95% CI 0.76‐0.78; pooled specificity 0.89, 95% CI 0.88‐0.90).

Fecal occult blood test (FOBT) and fecal immunohistochemistry test (FIT) are adopted in CRC screening. The FOBT has a sensitivity less than 50%.^92^ Aversion to handling stool is a cause for the low uptake of the fecal test, as only 58% of patients who are sent the FOBT return a sample. The pooled sensitivity for FIT was 0.79 (95% CI 0.69‐0.86) and specificity was 0.94 (95% CI 0.92‐0.95).^92^ Compared with liquid biopsy, FIT could be FP due to an upper gastrointestinal bleed that is large enough for hemoglobin to escape degradation during transit.^92^ Moreover, for early stage CRC, because there is usually no hemorrhage, the sensitivity and specificity for FIT are much lower, and have been estimated as 25%‐56% for sensitivity and 68% to 96% for specificity.^92^

Invasive and localized tumors may transfer CTCs into the bloodstream before obvious metastasis occurs, which indicates that individual detection of CTCs is more important^93^ Circulating tumor cells are mainly detected based on transcriptome or epithelial markers.^93^ The former analyses CRC‐derived CTCs through quantificational reverse transcription PCR (qRT‐PCR), while the later through epithelial markers, such as EpCAM and cytokeratins, that are not expressed in the ambient mesenchymal blood cells.^79^ The lack of standardized process of blood collection, biomarkers extraction, isolation, and final analysis contributed to the variance of sensitivity for CTCs in detecting CRC,^77^ which varied from 0.65 to 0.94 in the present study. As CTCs have a short life into the bloodstream, being quickly detected and destroyed in the liver by natural killer, the sensitivity of CTC was lower than its specificity.^76^ Compared with qRT‐PCR‐based methods, EpCAM‐based methods presented lower sensitivity but higher specificity: On one hand, EpCAM‐based methods could not detect the whole CTCs, since cells expressing low levels of epithelial markers could not be recognized by the capture reagent.^94^ However, small changes at transcriptome could still be detected easily by qRT‐PCR.^94^ On the other hand, EpCAM‐based methods detected CTCs on a series of tumor‐specific epithelial markers, which contributed to the high specificity.^94^ Even so, CTCs appeared to have highest sensitivity and specificity compared with cfDNA and exosomes, since patients with various benign inflammatory colon diseases also harbor viable cfDNA fragments or exosomes which could be detected through cfDNA or exosome‐based methods, leading to FP results.^28^

Exosomes are membrane microvesicles that are released by tumor cells and circulate in body fluids.^95^ CRC cells secrete significantly more exosomes than normal cells.^89^ These tumor‐derived exosomes could promote the transfer of small molecules, such as growth factors, chemokines, and DNA.^87^ Therefore, detecting exosomes in blood samples is a practicable method for CRC diagnosis. Besides, exosomes are stable at room temperature for a long time.^84^ Furthermore, protein markers of these exosomes could be adopted to predict future organ metastasis.^85^ In the present study, the pooled sensitivity, specificity, and AUC of exosomes were 0.76 (95% CI 0.72‐0.80), 0.92 (95% CI 0.89‐0.94), and 0.9037. The results showed that exosomes had a robust specificity, but the sensitivity was relatively low. Exosomes could transfer various bioactive molecules (such as proteins, lipids, and microRNA) from donor cells to recipient cells.^84^ The communication of substance and information between cancer cells was tight requiring special signal transmission and possibly secreting some special substances, which could contribute to the high specificity.^96^

Compared with exosomes and CTCs, cfDNA had the lowest specificity. The possible reasons could be as follows: (a) cfDNA derived from various cell types, including but not limited to tumor cells^8^; and (b) some articles took patients with polyps as control groups. Compared with healthy individuals, cell necrosis increased in polyps, which also led to an increase in cfDNA level. Nevertheless, the AUC of SROC for overall cfDNA was 0.8963, indicating cfDNA still had high diagnostic efficiency. A previous meta‐analysis tried to evaluate the diagnostic value of cfDNA^15^; however, it only focused on cfDNA without mutation and methylation gene, and included 14 articles in total. It showed that cfDNA had a sensitivity, specificity, and AUC of 0.735 (95% CI 0.713‐0.757), 0.918 (95% CI 0.900‐0.934), and 0.8818, respectively.^15^ Our research included cfDNA measured by CFD level, DNA integrity, and DNA methylation, revealing that cfDNA had higher diagnostic performance.

Cell‐free DNA was measured based on CFD level, DNA integrity, and DNA methylation. The sizes of cfDNA fragments discharged by necrotic tumor cells were different and longer, while the lengths of cfDNA fragments discharged by apoptotic nontumor cells were the same and shorter, ranging from 185 to 200 base pairs.^97^ Cell‐free DNA level was defined as relative or absolute concentration of all cfDNA, mainly the concentration of ALU115, which represented total cfDNA (long fragments and short fragments). ALU247 represented tumor cfDNA (long fragments). Integrity index calculated the ratio of ALU247 to ALU115, representing the relationship between long and short cfDNA fragments.^34^ Our results indicated that among all cfDNA measuring methods, DNA integrity was perhaps the best for CRC detection with the highest AUC (0.9187 vs 0.8838 [CFD] vs 0.8963 [DNA methylation]). However, it seemed to be less sensitive for CRC compared to the other two methods. CFD level was perhaps the best for CRC screening with highest sensitivity (0.77 vs 0.71 [DNA integrity] vs 0.76 [DNA methylation]). Generally, CFD level had a worse diagnostic value than DNA integrity, since apoptotic nontumor cells could also increase CFD level.^31^ Previous research demonstrated higher cfDNA concentration in patients with late‐stage diseases.^30^, ^32^ This advantage indicated that cfDNA could be used for the follow‐up of metastatic CRC patients during treatment.

Colorectal cancer could be determined by detecting DNA methylation level of several gene sites, SEPT9 is one of the most common sites.^40^, ^47^, ^98^ CpG island in the promoter region of V2 transcript of SEPT9 gene was usually hypermethylated in CRC, which was adopted as detection markers.^98^ Although Song et al^99^ published a meta‐analysis on the performance of SEPT9 gene methylation in CRC diagnosis, their study had several limitations: (a) included fewer articles; (b) some of the included articles had poor quality; and (c) did not compare diagnostic performance of SEPT9 methylation and methylation on other sites. Our results showed that the diagnostic performance of methylation on SEPT9 was similar to methylation on other sites.

There were three meta‐analyses focused on the diagnostic performance of cfDNA^15^, ^99^, ^100^; nevertheless, compared to them, our research had some advantages: (a) we not only focused on the diagnostic value of cfDNA, but also focused on the diagnostic value of CTCs, exosomes, and overall performance of all liquid biopsy methods^15^, ^99^, ^100^; (b) we compared diagnostic performance of CTCs, exosomes, and cfDNA^15^, ^99^, ^100^; and (c) we included much more articles.

Our meta‐analysis had several limitations. Firstly, as shown in Table 1, CTCs, exosomes, and cfDNA were the three major subtypes of liquid biopsy, while assay methods of cfDNA included measuring CFD level, DNA integrity, and methylation of DNA. These contributed to the heterogeneity for overall performance of all liquid biopsy. Secondly, the number of studies on CTCs and exosomes was much smaller than on cfDNA, which may introduce bias. Thirdly, only studies published in full text in English were included. Therefore, we only integrated sensitivity, specificity, PLR, NLR, and DOR of CTCs. Fourthly, unpublished studies were not included.

## 5 CONCLUSIONS

This meta‐analysis concluded that liquid biopsy was a powerful diagnostic tool in detecting CRC with high sensitivity and specificity, which had great potential for clinic application. Among all liquid biopsy methods, CTCs showed the best diagnostic performance with the highest AUC.

## CONFLICT OF INTEREST

The authors declare no conflict of interest. The funders had no role in the design of the study; in the collection, analyses, or interpretation of data; in the writing of the manuscript, or in the decision to publish the results.

## AUTHOR CONTRIBUTIONS

Yuzhou Zhu and Tinghan Yang contributed equally to this article. Yuzhou Zhu designed the project, developed the search strategy, and wrote the manuscript. Tinghan Yang checked the search, and reviewed the manuscript. Qingbin Wu and Xuyang Yang performed literature screening and data extraction. Jianqi Hao and Xiangbing Deng conducted the quality assessment of the included studies. Shuo Yang and Chaoyang Gu carried out the data analysis. Ziqiang Wang reviewed the manuscript and finally approved the version to be published.

## Supporting information

Supplementary Material

## Data Availability

This literature is a meta‐analysis. The original contributions presented in the study are included in the article/Supporting information, further inquiries can be directed to the corresponding author wangziqiang@scu.edu.cn.
