# Supplementary material for: Diagnostic performance of various liquid biopsy methods in detecting colorectal cancer: A meta‐analysis
Source: Cancer Med. 2020 Jul 6;9(16):5699–707. doi: 10.1002/cam4.3276 (PMC7433831; doi:10.1002/cam4.3276)
Supplement: Supplementary file 1 — Supplementary Material [file CAM4-9-5699-s001.docx]

**Diagnostic Performance of Various Liquid Biopsy Methods in Detecting Colorectal Cancer: A Meta-Analysis**

**Table S1: Search Strategy**

| Database | Search Strategy |
| --- | --- |
| Pubmed | ("Liquid Biopsy"[Mesh] OR liquid biopsy OR fluid biopsy OR circulating tumor cell OR "circulating tumor cell"[Mesh] OR CTC OR cell-free tumor DNA OR cfDNA OR ctDNA OR "circulating tumor DNA"[Mesh] OR circulating tumor DNA OR exosome OR "exosome"[Mesh] OR) AND (sensitivity OR “diagnosis”[Mesh] OR secificity OR diagnosis OR screening OR accuracy) AND ("Colorectal Neoplasms"[Mesh]OR Colorectal Neoplasm OR Colorectal Tumors OR Colorectal Tumor OR Colorectal Carcinoma OR Colorectal Carcinomas OR Colorectal Cancer OR colo* OR rect*) |
| Embase | ("Liquid Biopsy"[Mesh] OR liquid biopsy OR fluid biopsy OR circulating tumor cell OR "circulating tumor cell"[Mesh] OR CTC OR cell-free tumour DNA OR cfDNA OR ctDNA OR "circulating tumor DNA"[Mesh] OR circulating tumor DNA OR exosome OR "exosome"[Mesh]) AND (sensitivity OR “diagnosis”[Mesh] OR secificity OR diagnosis OR screening OR accuracy) AND ("Colorectal Neoplasms"[Mesh]OR Colorectal Neoplasm OR Colorectal Tumors OR Colorectal Tumor OR Colorectal Carcinoma OR Colorectal Carcinomas OR Colorectal Cancer OR colo* OR rect*) |
| Web of Science | (liquid biopsy OR fluid biopsy OR circulating tumor cell OR CTC OR cell-free tumour DNA OR cfDNA OR ctDNA OR circulating tumor DNA OR exosome) AND (sensitivity OR secificity OR diagnosis OR screening OR accuracy) AND (Colorectal Neoplasm OR Colorectal Tumors OR Colorectal Tumor OR Colorectal Carcinoma OR Colorectal Carcinomas OR Colorectal Cancer OR colo* OR rect*) |

**Table S2** Characteristics of 62 studies included in the meta-analysis

| Author | Year | | Country | | | Control group | | | TP | | FP | | FN | | TN | Number of cases(P/C) | | | Sen | Spe | | Subtype of CfDNA | | Marker |
| --- | --- | --- | --- | --- | --- | --- | --- | --- | --- | --- | --- | --- | --- | --- | --- | --- | --- | --- | --- | --- | --- | --- | --- | --- |
| **CfDNA** | | |  | |  |  | |  |  | |  | |  | |  | |  |  |  | | |  | |  |
| Nagai *et al* | 2017 | | Japan | | | Healthy | | | 75 | | 5 | | 39 | | 48 | 167(114/53) | | | 65.8 | 90 | | DNA integrity | | LINE-1 hypomethylation index in cfDNA |
| Umetani *et al*. | 2006 | | USA | | | Healthy | | | 18 | | 5 | | 14 | | 46 | 83(32/51) | | | 40.6 | 90.2 | | DNA integrity | | ALU247/115 |
| El-Gayar *et al*. | 2016 | | Egypt | | | Healthy control | | | 45 | | 3 | | 5 | | 17 | 70(50/20) | | | 90.0 | 85.0 | | DNA integrity | | ALU 247/115 |
| Hao *et al.* | 2015 | | China | | | Healthy + polyps | | | 76 | | 5 | | 28 | | 168 | 277(104/173) | | | 73.1 | 97.3 | | DNA integrity | | ALU247/115 |
| Hao *et al.* | 2015 | | China | | | Healthy+ polyps | | | 72 | | 2 | | 32 | | 171 | 246(104/173) | | | 69.2 | 99.1 | | CFD level | | ALU115 |
| Umetani *el al.* | 2006 | | USA | | | Healthy | | | 13 | | 5 | | 19 | | 46 | 83(32/51) | | | 41.0 | 90.0 | | CFD level | | ALU115 |
| Danese *et al.* | 2010 | | Italy | | | Healthy + polyps | | | 98 | | 2 | | 20 | | 24 | 144(118/26) | | | 82.9 | 92.3 | | CFD level | | NA |
| Agostini *et al.* | 2011 | | Italy | | | Healthy | | | 63 | | 0 | | 4 | | 35 | 102(67/35) | | | 94.0 | 100.0 | | CFD level | | Alu 247 |
| Czeiger *et al.* | 2011 | | Israel | | | Healthy | | | 16 | | 2 | | 22 | | 32 | 72(38/34) | | | 42.0 | 94.0 | | CFD level | | Arbitrary fluorescence units at 535 nm |
| Qi *et al.* | 2013 | | China | | | Healthy | | | 20 | | 1 | | 11 | | 91 | 123(31/92) | | | 64.5 | 98.9 | | CFD level | | Alu-based |

| **Table S2(continued)** Characteristics of 62 studies included in the meta-analysis | | | | | | | | | | | | |
| --- | --- | --- | --- | --- | --- | --- | --- | --- | --- | --- | --- | --- |
| Author | Year | Country | Control group | TP | FP | FN | TN | Number of cases(P/C) | Sen | Spe | Subtype of CfDNA | Marker |
| Leszinski *et al.* | 2014 | German | Healthy | 18 | 7 | 6 | 17 | 48(24/24) | 75.0 | 70.8 | CFD level | Alu-based |
| El-Gayar *et al.* | 2016 | Egypt | Healthy | 34 | 7 | 16 | 13 | 70(50/20) | 68.0 | 65.0 | CFD level | ALU115 |
| Berger *et al.* | 2017 | Germany | Healthy | 14 | 3 | 1 | 35 | 53(15/38) | 93.3 | 92.1 | CFD level | NA |
| Lan *et al.* | 2017 | China | Healthy | 272 | 4 | 57 | 91 | 424(329/95) | 82.7 | 95.8 | CFD level | NA |
| Flamini *et al.* | 2006 | Italy | Healthy | 61 | 20 | 14 | 55 | 150(75/75) | 81.3 | 73.3 | CFD level | glyceraldehyde-3-phosphate  dehydrogenase housekeeping gene |
| Oh *et al* | 2013 | South Korea | Healthy | 114 | 6 | 17 | 119 | 256(131/125) | 87.0 | 95.2 | DNA methylation | Methylation of SDC2 |
| Pedersen *et al.* | 2015 | Australia | Healthy | 85 | 50 | 44 | 788 | 967(129/838) | 65.6 | 94.0 | DNA methylation | Methylation of BCAT1,  IKZF1 joint |
| Leung *et al.* | 2005 | China | Healthy | 28 | 4 | 21 | 37 | 90(49/41) | 57.1 | 90.2 | DNA methylation | Methylated of HLTF |

| **Table S2(continued)** Characteristics of 62 studies included in the meta-analysis | | | | | | | | | | | | |
| --- | --- | --- | --- | --- | --- | --- | --- | --- | --- | --- | --- | --- |
| Author | Year | Country | Control group | TP | FP | FN | TN | Number of cases(P/C) | Sen | Spe | Subtype of CfDNA | Marker |
| Rasmussen *et al.* | 2017 | Denmark | Healthy | 175 | 28 | 18 | 74 | 295(193/102) | 90.7 | 72.5 | DNA methylation | Methylation of seven gene promoter regions |
| Perez-Carbonell *et al.* | 2014 | Spain | Healthy | 406 | 2 | 19 | 19 | 446(425/21) | 95.5 | 90.5 | DNA methylation | Methylation of miR137 and IGFBP3 joint |
| Kostin *et al.* | 2010 | Russia | Healthy | 45 | 8 | 10 | 66 | 126(55/71) | 81.0 | 93.0 | DNA methylation | Methylation of SEPT9, CDH1, HLTF and ALX4 joint |
| Zhang *et al.* | 2015 | China | Healthy | 46 | 21 | 11 | 26 | 104(57/47) | 80.7 | 55.32 | DNA methylation | Methylation of GATA5, SFRP2 and ITGA4 joint |
| Melotte *et al.* | 2015 | Germany | Healthy | 125 | 67 | 95 | 617 | 904(220/684) | 56.8 | 89.1 | DNA methylation | Methylation of FOXE1/SYNE1 |
| Takane *et al* | 2013 | Japan | Non-CRC | 108 | 25 | 12 | 72 | 217(120/97) | 90.0 | 64.0 | DNA methylation | Methylation of PPP1R3C and EFHD1 joint |
| Roperch *et al.* | 2013 | France | Non-CRC | 28 | 32 | 4 | 129 | 193(107/86) | 87.0 | 80.0 | DNA methylation | Methylation of NPY |
|  |  |  |  | 25 | 16 | 7 | 145 | 193(107/86) | 78.0 | 90.0 | DNA methylation | Methylation of PENK |
|  |  |  |  | 19 | 8 | 13 | 153 | 193(107/86) | 59.0 | 95.0 | DNA methylation | Methylation of WIF1 |

| **Table S2(continued)** Characteristics of 62 studies included in the meta-analysis | | | | | | | | | | | | |
| --- | --- | --- | --- | --- | --- | --- | --- | --- | --- | --- | --- | --- |
| Author | Year | Country | Control group | TP | FP | FN | TN | Number of cases(P/C) | Sen | Spe | Subtype of CfDNA | Marker |
| Cassinotti, *et al.* | 2012 | The US | healthy | 25 | 10 | 5 | 20 | 60(30/30) | 83.7 | 67.9 | DNA methylation | Methylation of 6 joint gene |
| Tang *et al* | 2011 | China | Non-CRC | 113 | 4 | 56 | 59 | 232(169/63) | 66.9 | 93.7 | DNA methylation | Methylation of SFRP2 |
| Herbst *et al* | 2011 | Germany | Healthy | 61 | 9 | 36 | 36 | 142(61/81) | 62.9 | 80.0 | DNA methylation | Methylation of NEUROG1 |
| Sakamoto *et al* | 2010 | Japan | Non-CRC | 22 | 0 | 29 | 20 | 71(51/20) | 43.1 | 100.0 | DNA methylation | Methylation of P16 |
| Ebert *et al* | 2006 | The US | Non-CRC | 27 | 3 | 14 | 16 | 60(30/30) | 90.0 | 53.3 | DNA methylation | Methylation of ALX4 |
| Grutzmann, *et al.* | 2008 | The US | Healthy | 73 | 18 | 53 | 165 | 309(126/183) | 57.9 | 90.2 | DNA methylation | Methylation of SEPT9 |
| Lofton-Day *et al.* | 2008 | Germany | Healthy | 92 | 25 | 41 | 154 | 312(133/179) | 69.0 | 86.0 | DNA methylation | Methylation of SEPT9 |
| He *et al.* | 2010 | China | Healthy | 136 | 6 | 46 | 164 | 352(182/170) | 74.7 | 96.5 | DNA methylation | Methylation of SEPT9 |
| Tanzer *et al.* | 2010 | Germany | Healthy | 27 | 4 | 6 | 30 | 67(33/34) | 82.0 | 88.0 | DNA methylation | Methylation of SEPT9 |
| Warren *et al.* | 2011 | The US | Healthy | 45 | 11 | 5 | 93 | 144(50/104) | 90.0 | 88.0 | DNA methylation | Methylation of SEPT9 |
| Ahlquist *et al.* | 2012 | The US | Healthy | 18 | 9 | 12 | 34 | 73(30/43) | 60.0 | 79.1 | DNA methylation | Methylation of SEPT9 |
| Toth *et al.* | 2012 | Hungary | Healthy | 88 | 14 | 4 | 78 | 184(92/92) | 95.6 | 84.8 | DNA methylation | Methylation of SEPT9 |

| **Table S2(continued)** Characteristics of 62 studies included in the meta-analysis | | | | | | | | | | | | |
| --- | --- | --- | --- | --- | --- | --- | --- | --- | --- | --- | --- | --- |
| Author | Year | Country | Control group | TP | FP | FN | TN | Number of cases(P/C) | Sen | Spe | Subtype of CfDNA | Marker |
| Liu *et al.* | 2013 | China | Healthy | 20 | 2 | 17 | 18 | 57(37/20) | 54.1 | 90.0 | DNA methylation | Methylation of SEPT9 |
| Church *et al.* | 2014 | The US | Healthy + adenoma | 34 | 169 | 19 | 1288 | 1510(53/1457) | 63.9 | 88.4 | DNA methylation | Methylation of SEPT9 |
| Potter *et al.* | 2014 | Germany | Healthy+ adenoma | 30 | 300 | 14 | 1200 | 1544(44/1500) | 68.2 | 80.0 | DNA methylation | Methylation of SEPT9 |
| Johnson *et al.* | 2014 | The US | Healthy + adenoma | 74 | 37 | 27 | 163 | 301(101/200 | 73.3 | 81.5 | DNA methylation | Methylation of SEPT9 |
| Toth *et al.* | 2014 | Hungary | Healthy | 30 | 2 | 4 | 22 | 58(34/24) | 88.2 | 91.7 | DNA methylation | Methylation of SEPT9 |
| Kang *et al.* | 2014 | China | Healthy | 60 | 1 | 20 | 51 | 132(80/52) | 75.0 | 98.1 | DNA methylation | Methylation of SEPT9 |
| Jin *et al.* | 2015 | China | Healthy | 101 | 3 | 34 | 88 | 226(135/91) | 74.8 | 96.7 | DNA methylation | Methylation of SEPT9 |
| Ørntoft, *et al.* | 2015 | Denmark | Healthy | 93 | 27 | 35 | 123 | 278(128/150) | 72.7 | 82.0 | DNA methylation | Methylation of SEPT9 |
| Wu *et al.* | 2016 | China | Healthy | 223 | 12 | 68 | 283 | 586(291/295) | 76.6 | 95.9 | DNA methylation | Methylation of SEPT9 |
| Song *et al.* | 2016 | China | Healthy | 303 | 88 | 66 | 402 | 859(369/490) | 82.4 | 82.0 | DNA methylation | Methylation of SEPT9 |
| DeVos *et al.* | 2009 | Germany | Healthy | 61 | 17 | 29 | 138 | 245(90/155) | 67.8 | 89.0 | DNA methylation | Methylation of SEPT9 |
| Sun *et al.* | 2019 | China | Healthy | 46 | 32 | 17 | 555 | 650(63/587) | 73.0 | 94.5 | DNA methylation | Methylation of SEPT9 |
| He *et al* | 2018 | China | Without cancer | 221 | 99 | 79 | 694 | 1093(300/793) | 73.7 | 87.5 | DNA methylation | Methylation of SEPT9 |
| Fu *et al* | 2018 | China | Healthy | 60 | 16 | 38 | 237 | 341(98/243) | 61.2 | 93.7 | DNA methylation | Methylation of SEPT9 |

| **Table S2(continued)** Characteristics of 62 studies included in the meta-analysis | | | | | | | | | | | | | | | | | | | | | | | | | | | | | | | | | | | | |
| --- | --- | --- | --- | --- | --- | --- | --- | --- | --- | --- | --- | --- | --- | --- | --- | --- | --- | --- | --- | --- | --- | --- | --- | --- | --- | --- | --- | --- | --- | --- | --- | --- | --- | --- | --- | --- |
| Author | | | | Year | | Country | | | | Control group | | | | | | | TP | | FP | FN | | | TN | | | | | Number of cases(P/C) | Sen | | | Spe | | | Subtype of CfDNA | Marker |
| Molparia *et al.* | | | | 2018 | | America | | | | Without cancer | | | | | | | 19 | | 0 | 5 | | | 25 | | | | | 49(24/25) | 79.2 | | | 100 | | | NA | copy numbers variations |
| R.Borchers, *et al.* | | | | 2002 | | Germany | | | | Healthy | | | | | | | 5 | | 0 | 11 | | | 20 | | | | | 36(16/20) | 31.3 | | | 100 | | | NA | K-ras |
| Valladares, *et al*. | | | | 2012 | | Spain | | | | Healthy | | | | | | | 36 | | 1 | 18 | | | 18 | | | | | 73(54/19) | 67.4 | | | 94.7 | | | NA | AGR2 and LGR5 |
| **CTCs** | | |  | |  | | |  | |  | | |  | |  | |  | |  | | | |  | | |  | |  | | | |  | | |  |  |
| Tsai *et al.* | | | | 2018 | | Taiwan | | | | Healthy | | | | | | | 285 | | 6 | 42 | | | 176 | | | | | 509(327/182) | 87.2 | | | 96.7 | | | NA | EpCAM+CK20+/CD45- |
| Chen *et al.* | | | | 2016 | | Tai wan | | | | Healthy | | | | | | | 35 | | 1 | 19 | | | 8 | | | | | 63(54/9) | 64.8 | | | 88.9 | | | NA | NA |
| Wong *et al* | | | | 2011 | | China | | | | Without cancer | | | | | | | 73 | | 3 | 17 | | | 167 | | | | | 260(90/170) | 81.0 | | | 98.2 | | | NA | CDX2 |
| Wang *et al* | | | | 2006 | | Taiwan | | | | Healthy | | | | | | | 83 | | 3 | 5 | | | 47 | | | | | 138(88/50) | 94.3 | | | 94.0 | | | NA | 18 oligonucleotide clones |
| Douard *et al* | | | | 2005 | | France | | | | Healthy | | | | | | | 53 | | 0 | 31 | | | 41 | | | | | 125(84/41) | 63.0 | | | 100 | | | NA | CECAM5,CEACAM7 |
| **Exosomes** | | |  | |  | |  | | |  | | |  | |  | |  | |  | | | |  | | |  | |  | | | |  | | |  |  |
| Sun *et al.* | | | | 2019 | | China | | | | Healthy | | | | | | | 62 | | 5 | 30 | | | 27 | | | | | 124(92/32) | 67.4 | | | 84.4 | | | NA | Exosomal CPNE3 |
| Liu *et al.* | | | | 2016 | | China | | | | Without cancer | | | | | | | 104 | | 18 | 48 | | | 302 | | | | | 468(152/320) | 68.4 | | | 94.4 | | | NA | long noncoding RNA |

| **Table S2(continued)** Characteristics of 62 studies included in the meta-analysis | | | | | | | | | | | | |
| --- | --- | --- | --- | --- | --- | --- | --- | --- | --- | --- | --- | --- |
| Author | Year | Country | Control group | TP | FP | FN | TN | Number of cases(P/C) | Sen | Spe | Subtype of CfDNA | Marker |
| Liu *et al* | 2018 | China | Healthy | 68 | 4 | 12 | 36 | 120(80/40) | 85.0 | 90.0 | NA | Exosomal  miR-27a and miR-130a combination |
| Ogata-Kawata, *et al.* | 2014 | Japan | Healthy | 81 | 0 | 7 | 11 | 99(88/11) | 92.0 | 100 | NA | miR-23a |
| Graham *et al.* | 2011 | Australia | Healthy | 13 | 1 | 2 | 14 | 30(15/15) | 86.7 | 93.3 | NA | CRNDE-h |
| Uratani *et al.* | 2016 | Japan | Healthy + adenomas | 18 | 9 | 8 | 38 | 73(26/47) | 69.2 | 80.9 | NA | miRNAs, miR-21, miR-29a, miR-92a, and miR135b |
| TP = true-positive; FP = false-positive; FN = false-negative; TN = true-negative; P/C = numbers of patients/control; Sen/Spe = Sensitivity/ Specificity; cfDNA = cell-free DNA; CTCs = circulating tumor cells; CFD level = cell-free DNA level; NA = not applicable; qMSP = Quantitative Methylation-specific PCR; qPCR = quantitative polymerase chain reaction; ddPCR = Droplet digital PCR; RFLP-PCR = Restriction Fragment Length Polymorphism PCR; qRT-PCR = Quantitative reverse transcriptase PCR; | | | | | | | | | | | | |


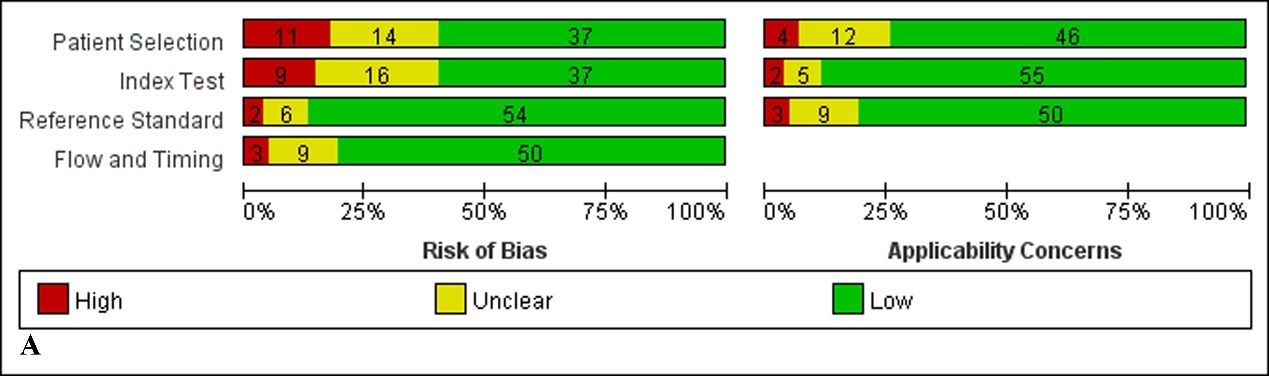


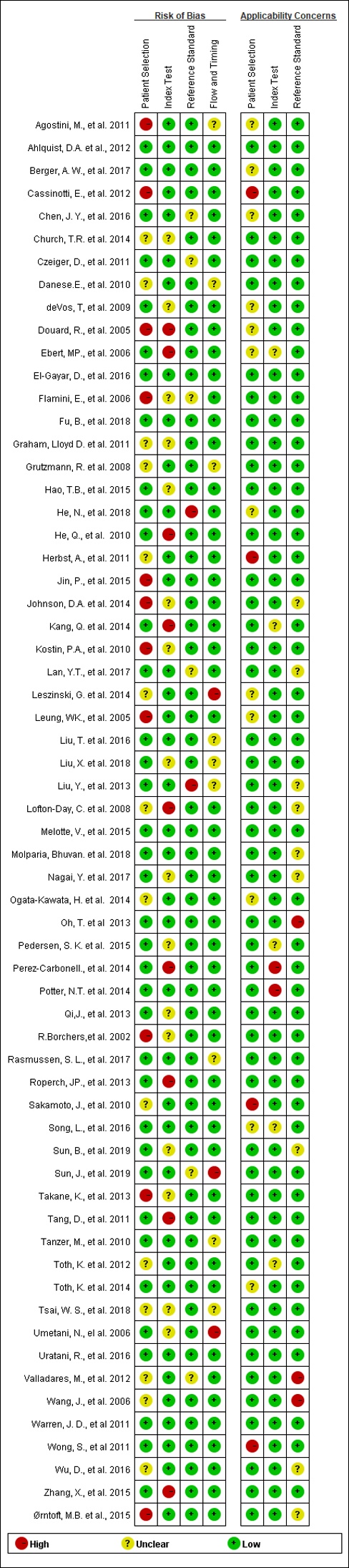


**B**

**Figure S1**. Quality assessment of studies by Quadas-2 evaluation tool. (**A**) Risk of bias graph. (**B**) Risk of summary.


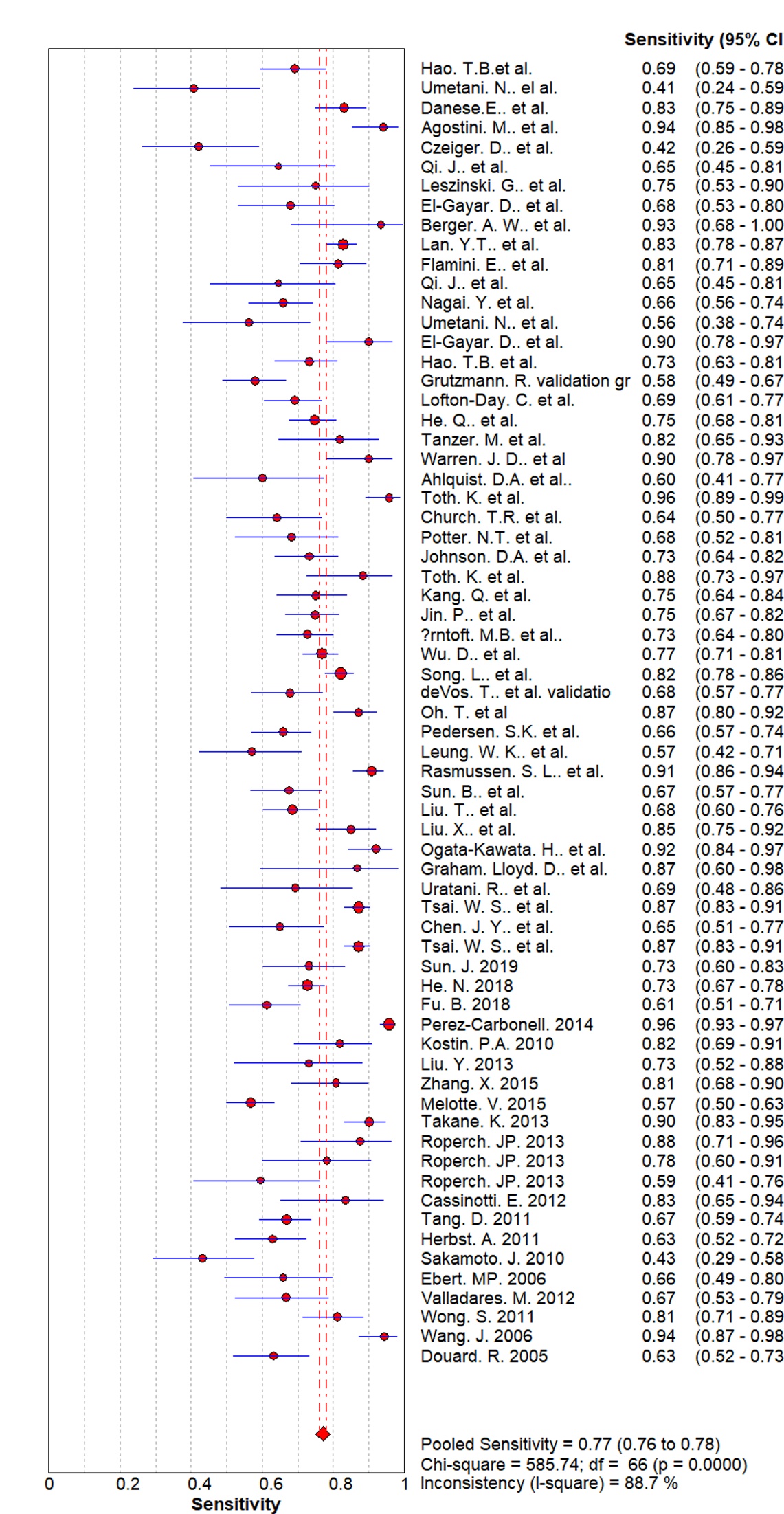


**A**


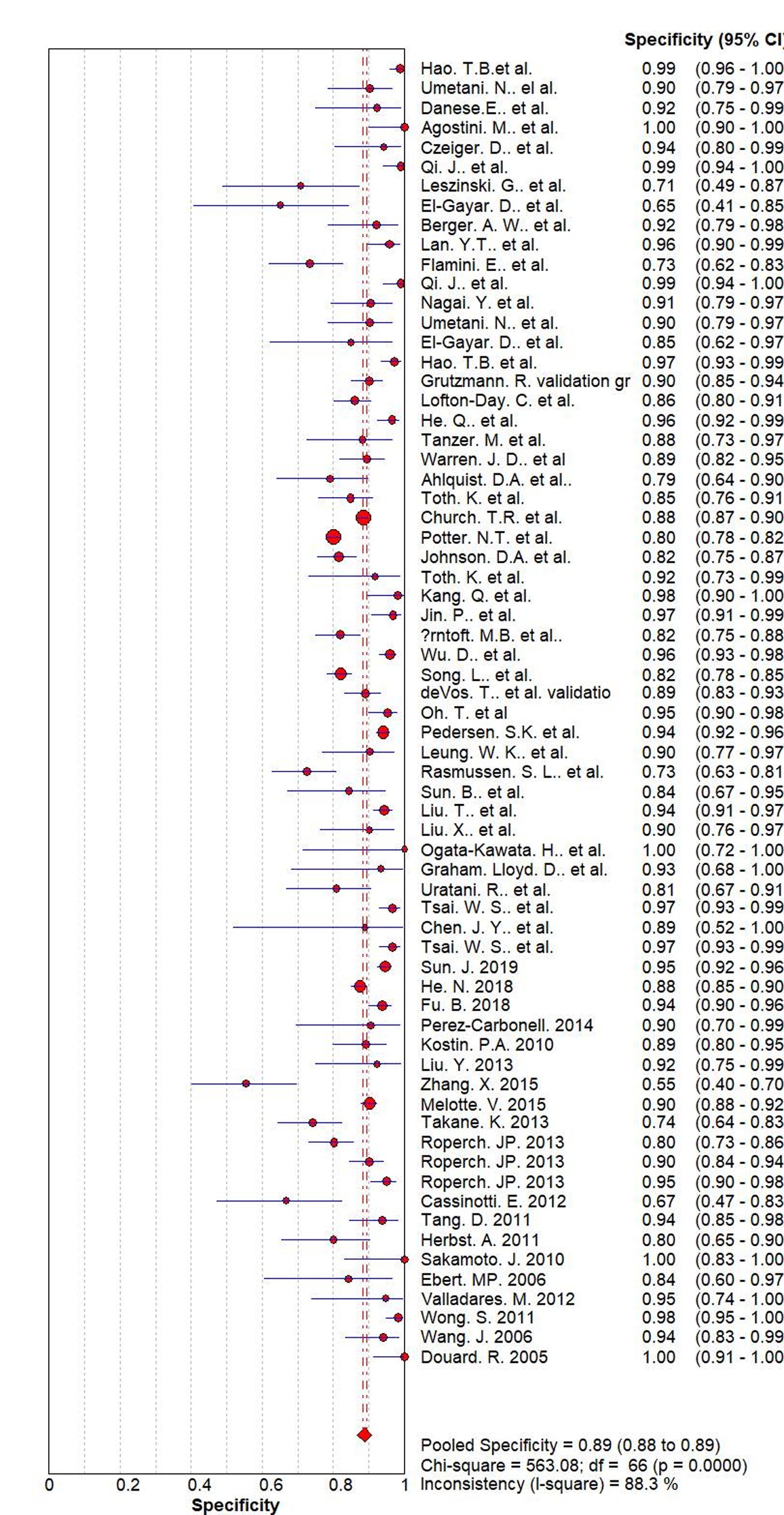


**B**


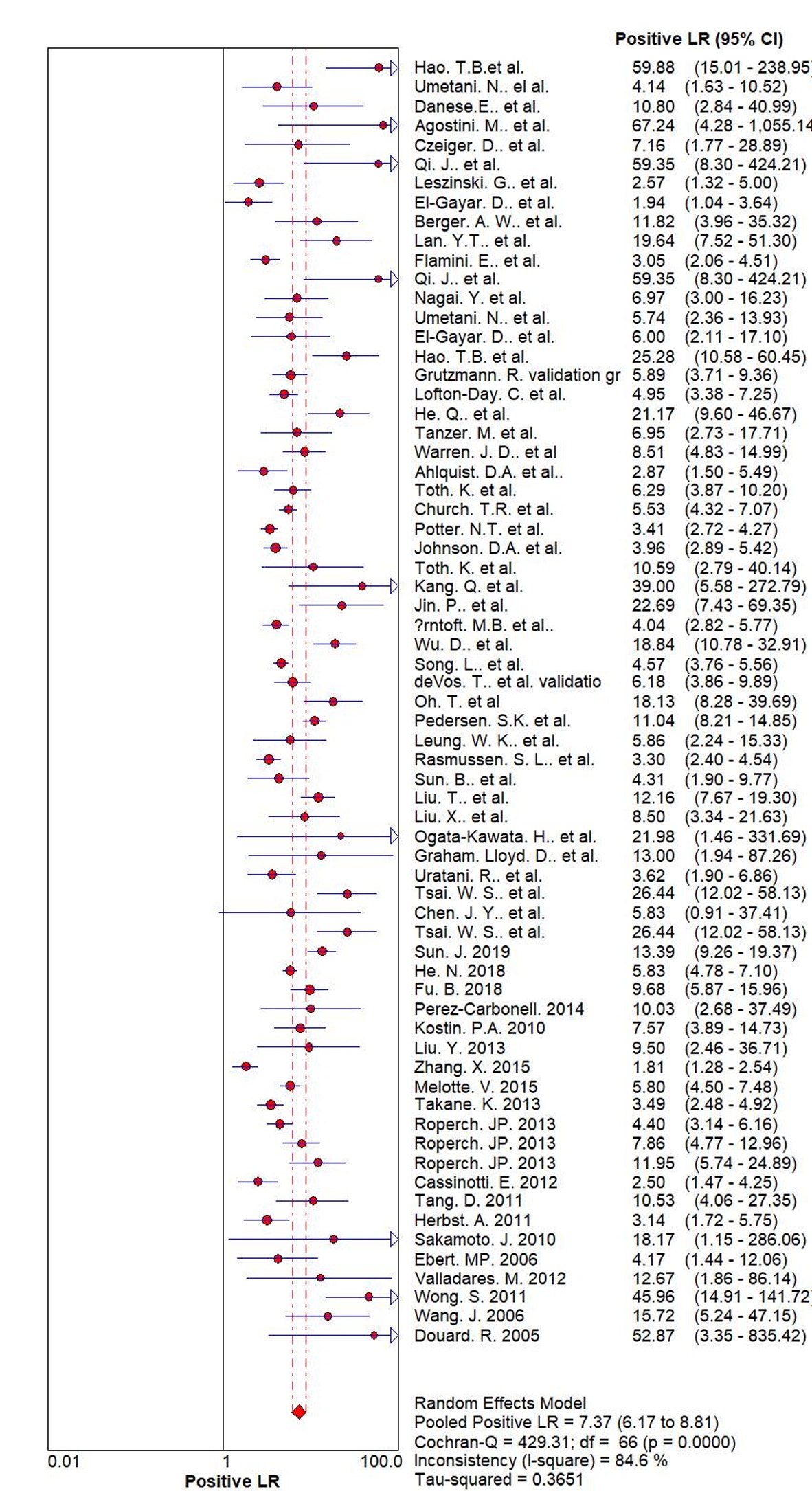

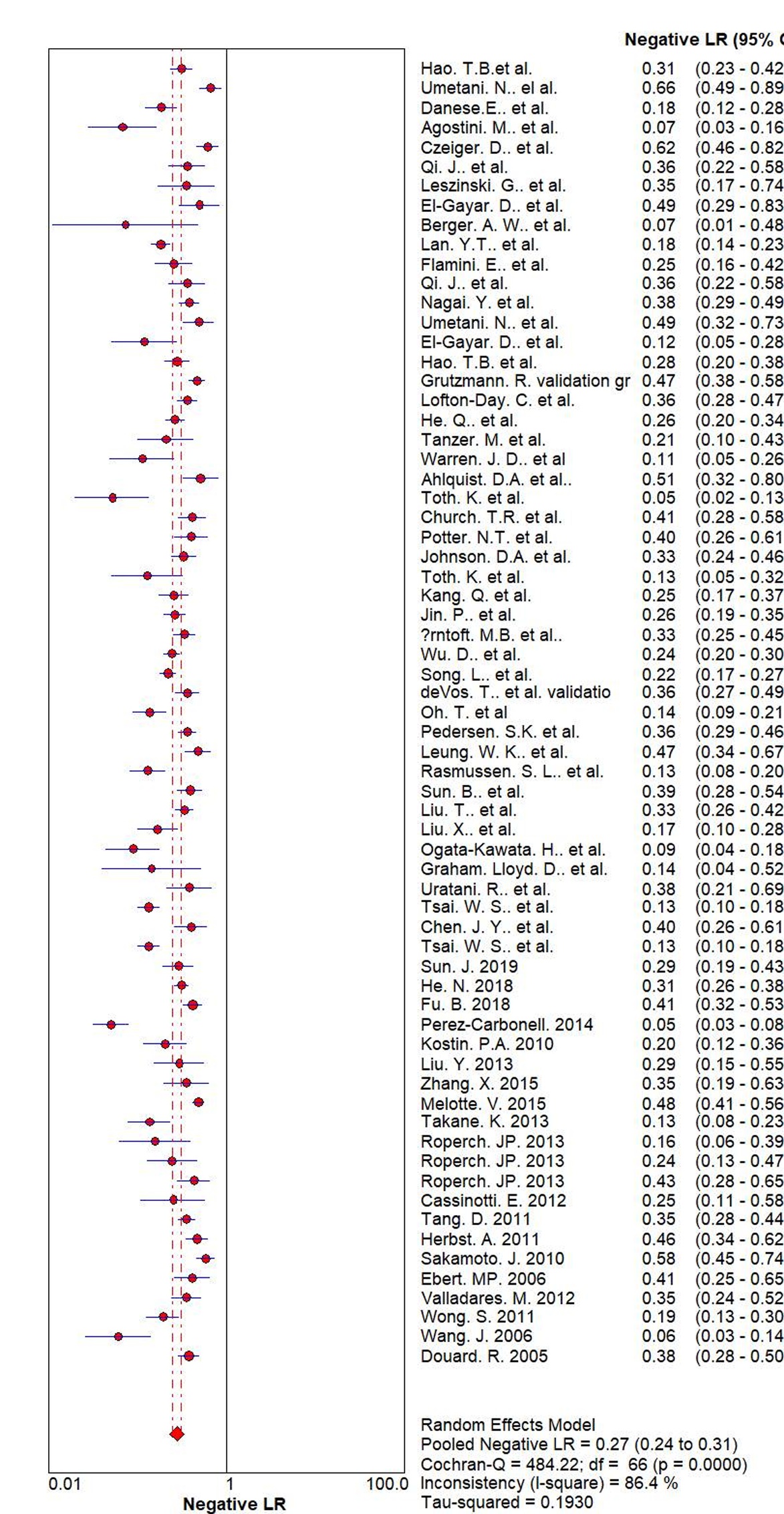

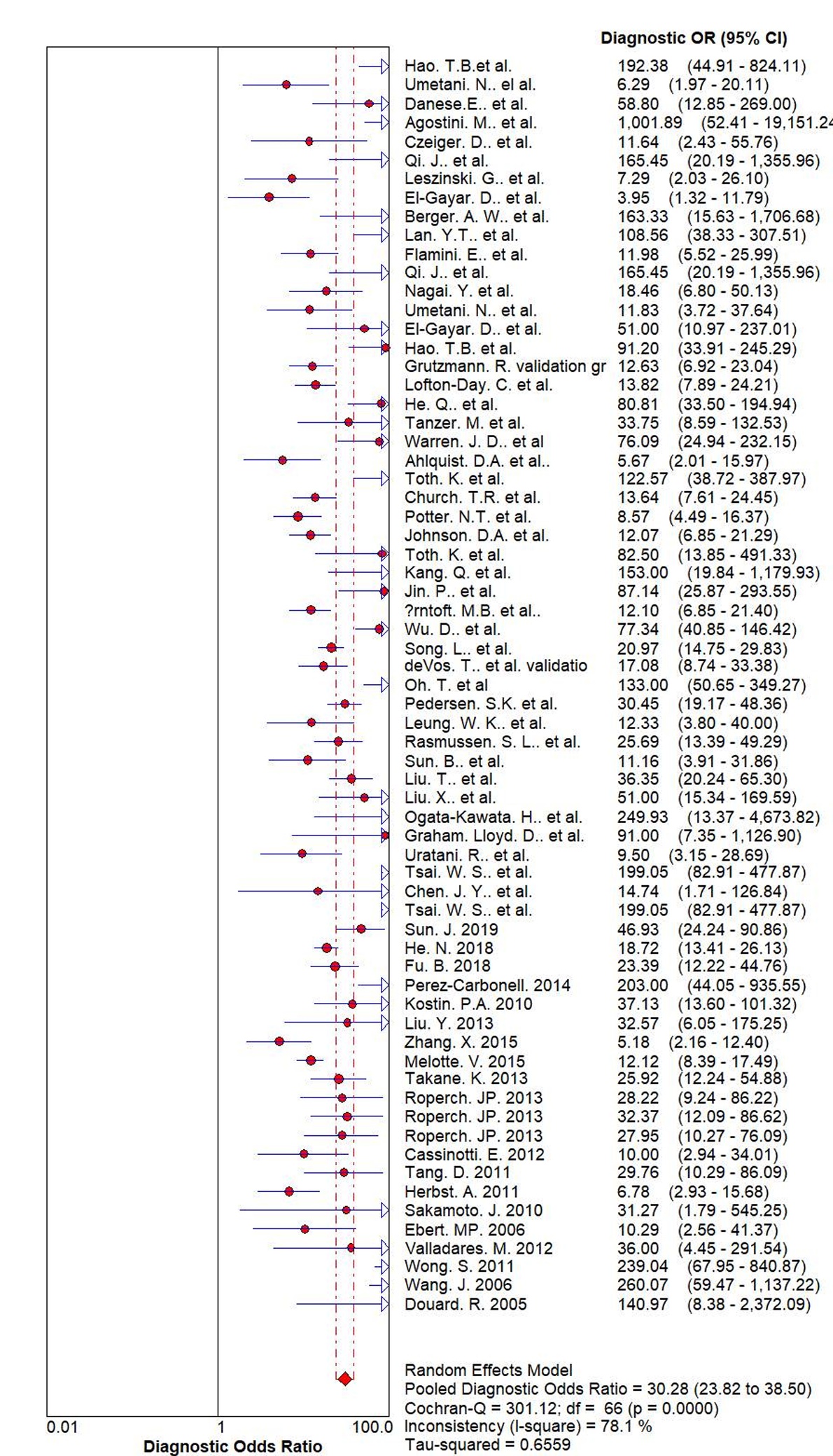

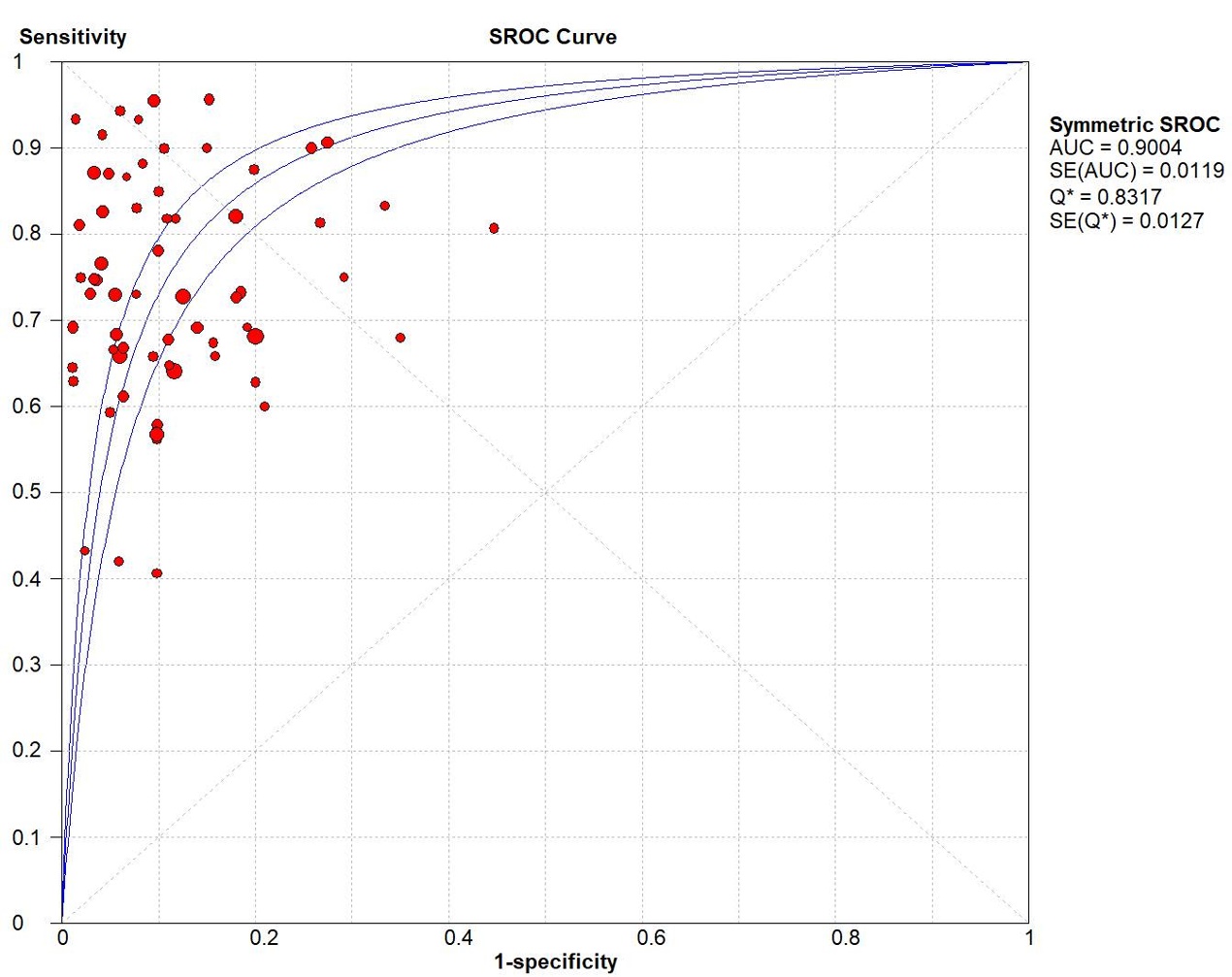


**C**

**D**

**E**

**F**

**Figure S2.** Forest plots of the diagnostic value for overall liquid biopsy in detecting colorectal cancer. (**A**) Sensitivity. (**B**) Specificity. (**C**) positive likelihood ratio. (**D**) negative likelihood ratio. (**E**) Diagnostic odds ratio. (**F**) SROC curve.


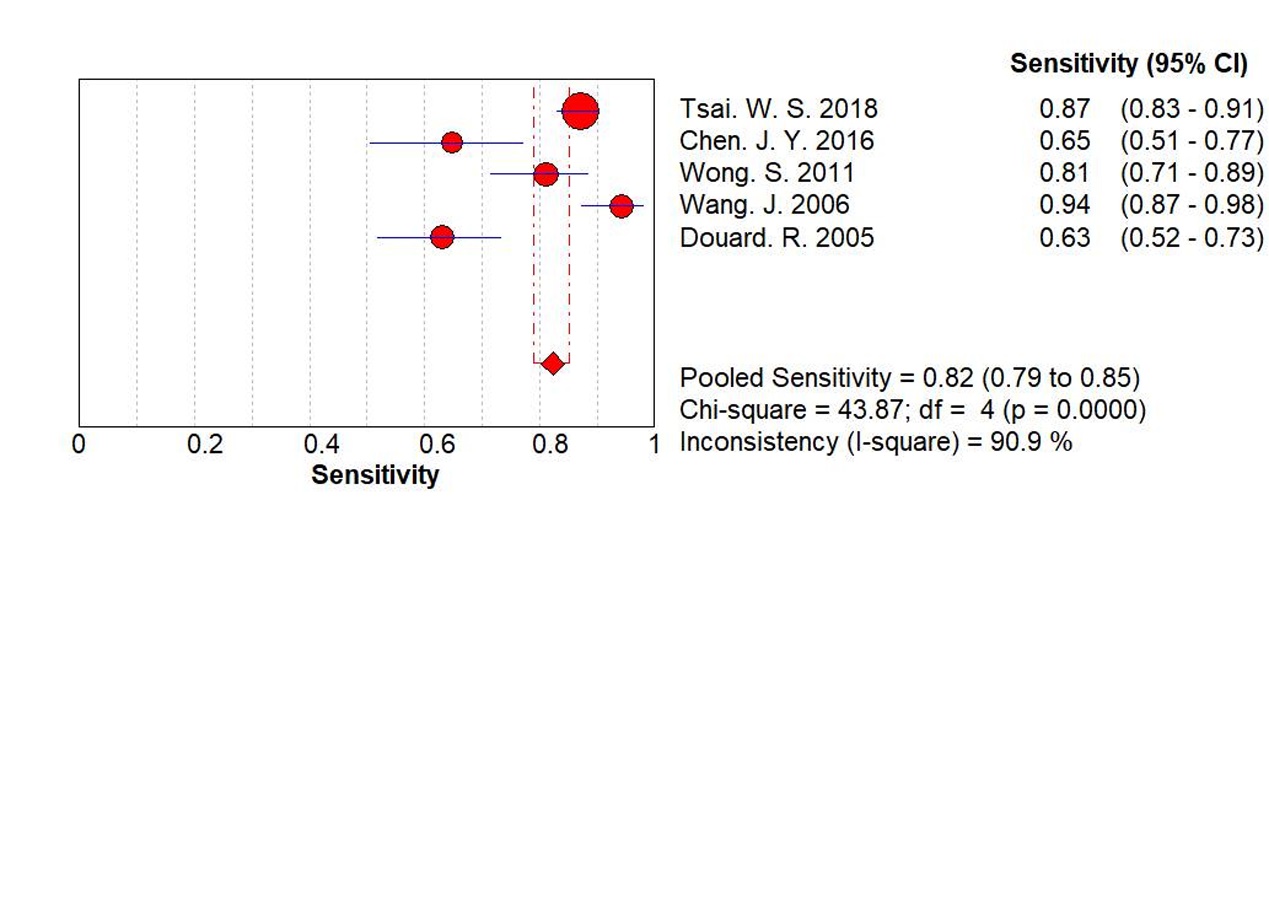


**A**


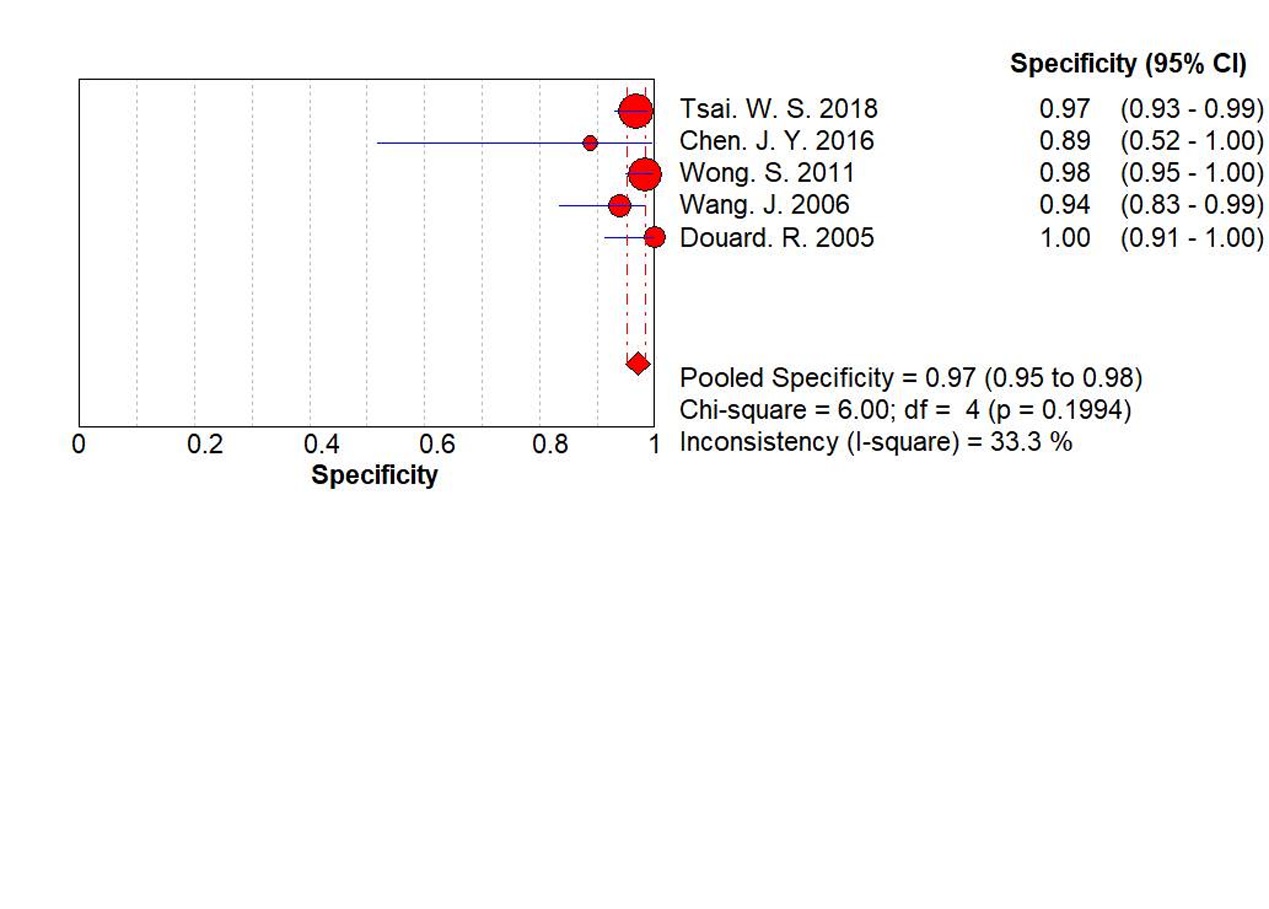


**B**


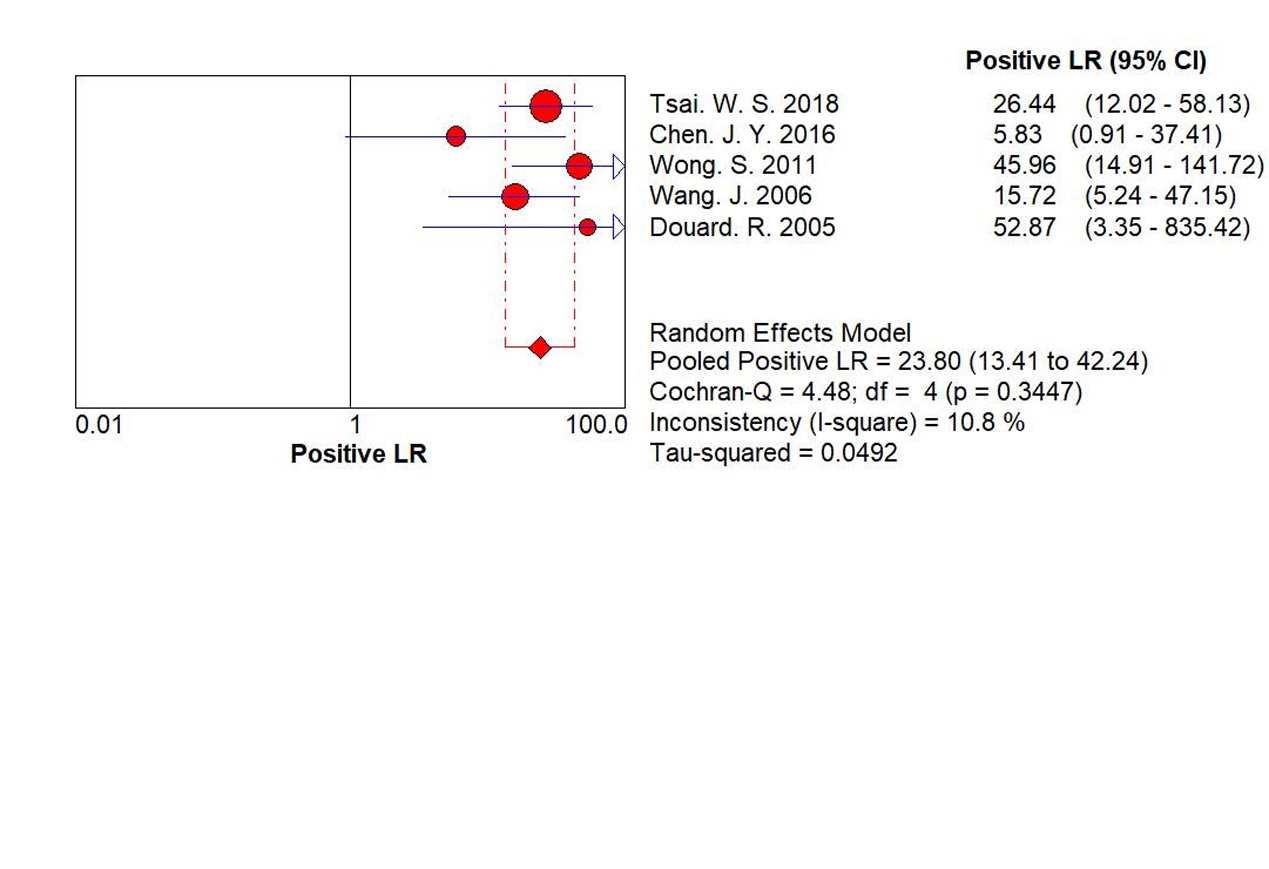


**C**


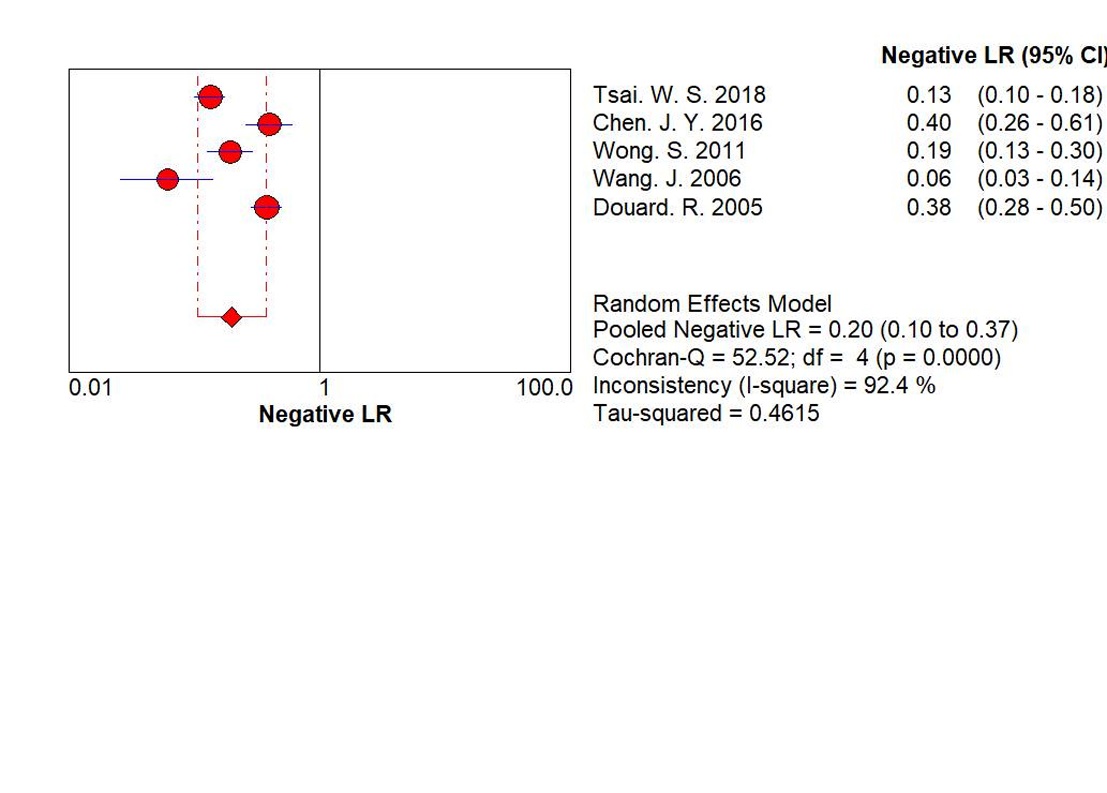

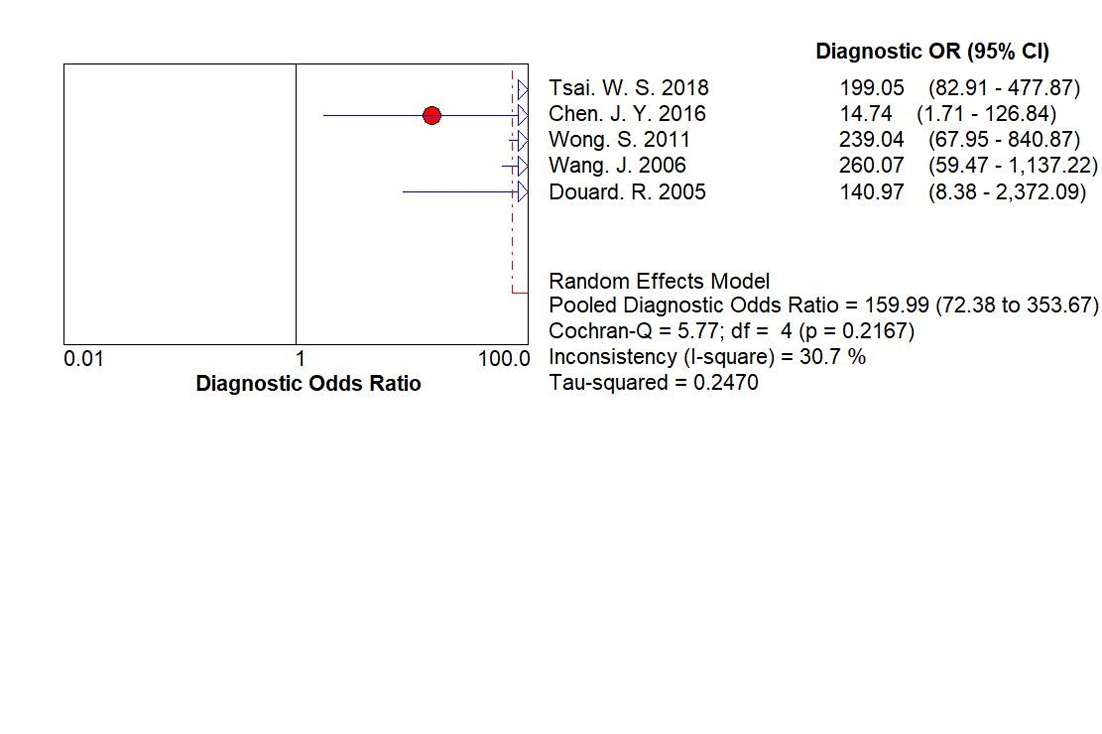


**E**

**D**


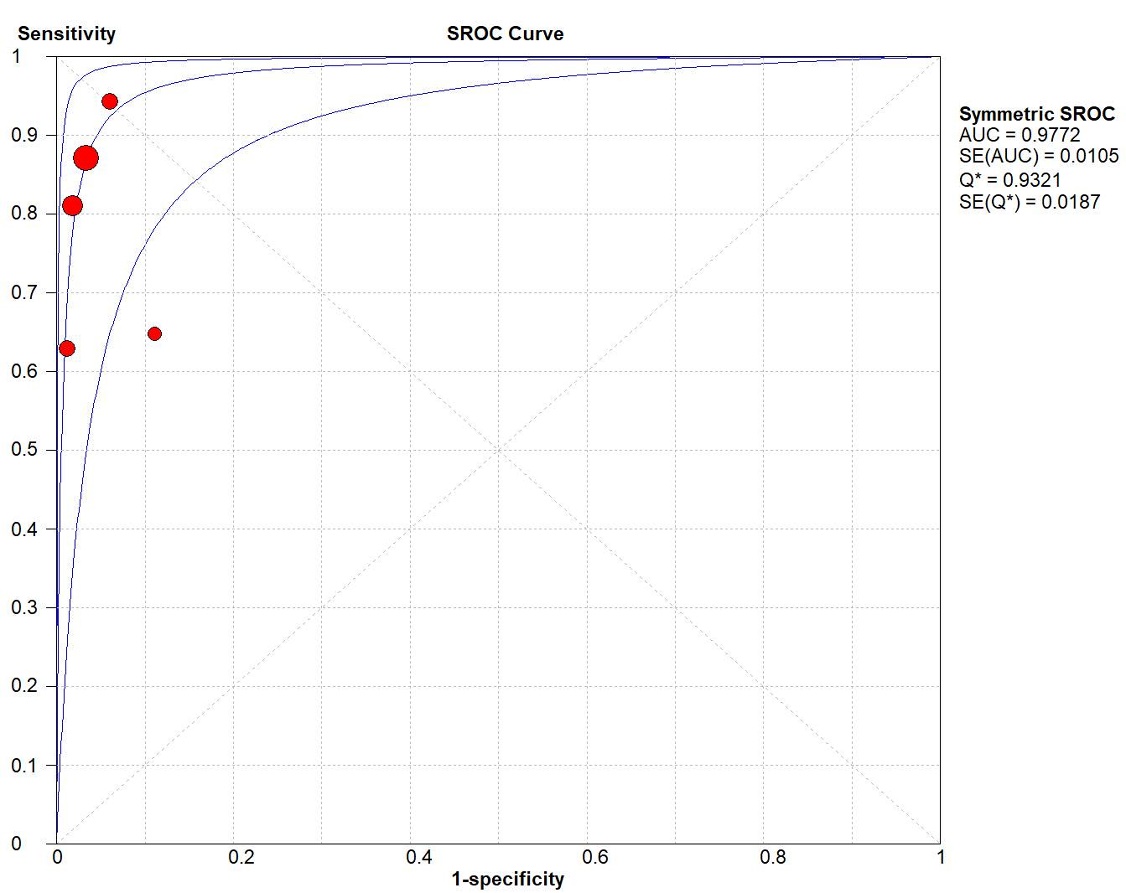


**F**

**Figure S3.** Forest plots of the diagnostic value for CTCs in detecting colorectal cancer. (**A**) Sensitivity. (**B**) Specificity. (**C**) positive likelihood ratio. (**D**) negative likelihood ratio. (**E**) Diagnostic odds ratio. (**F**) SROC curve.


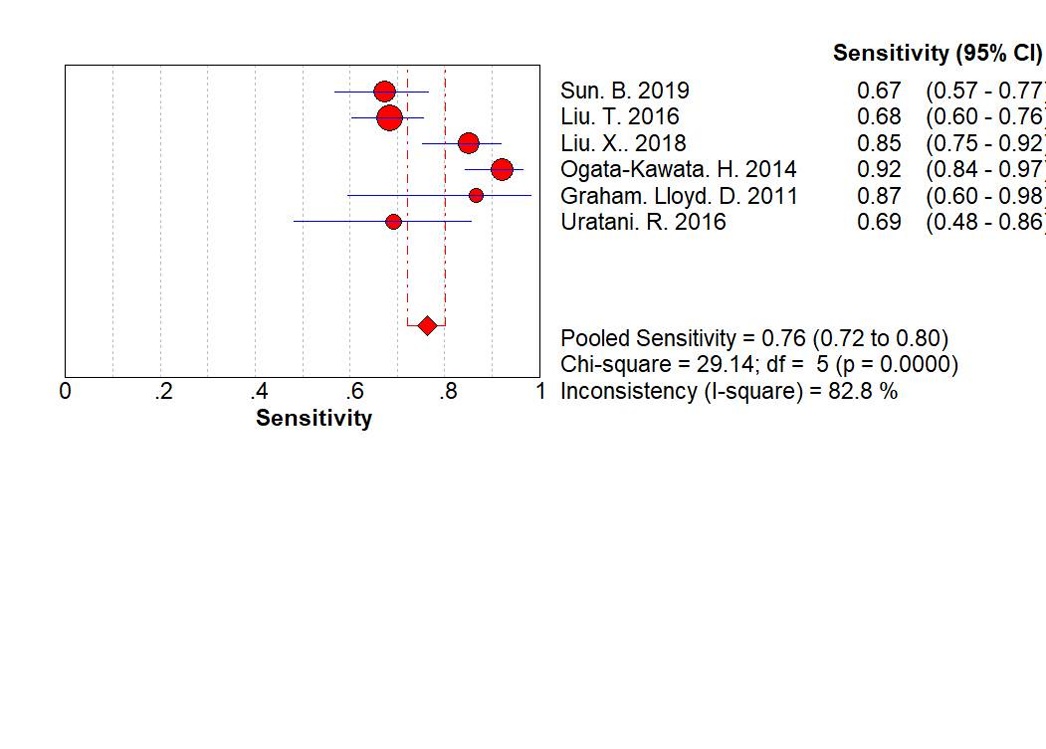


**A**


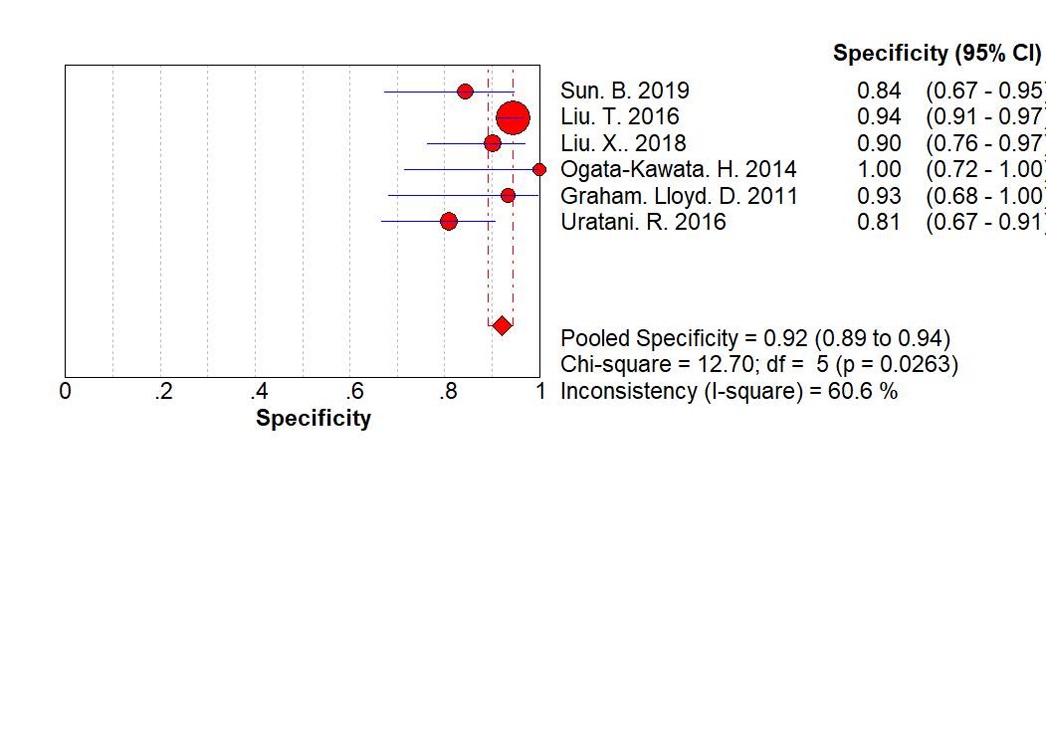

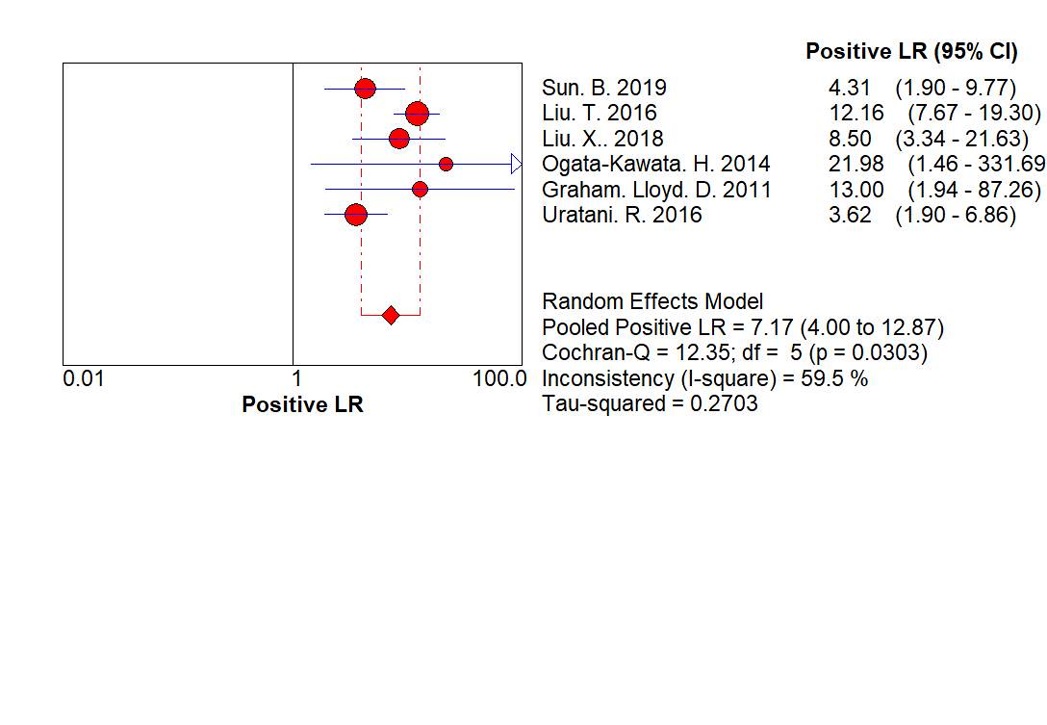


**C**

**B**


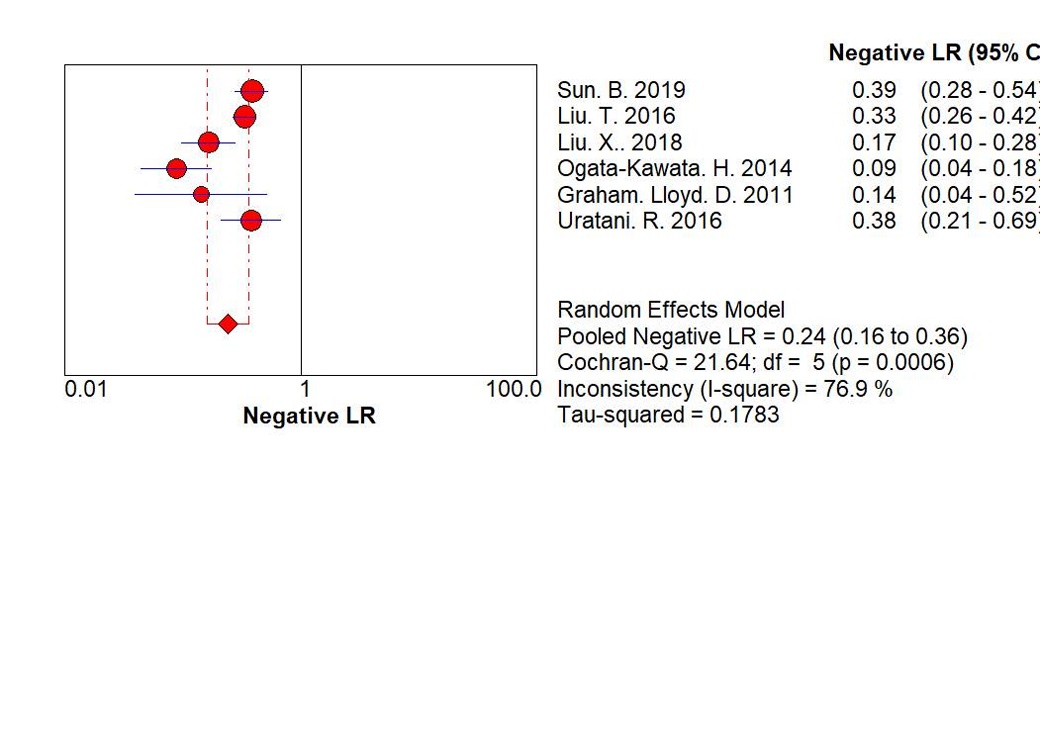


**D**


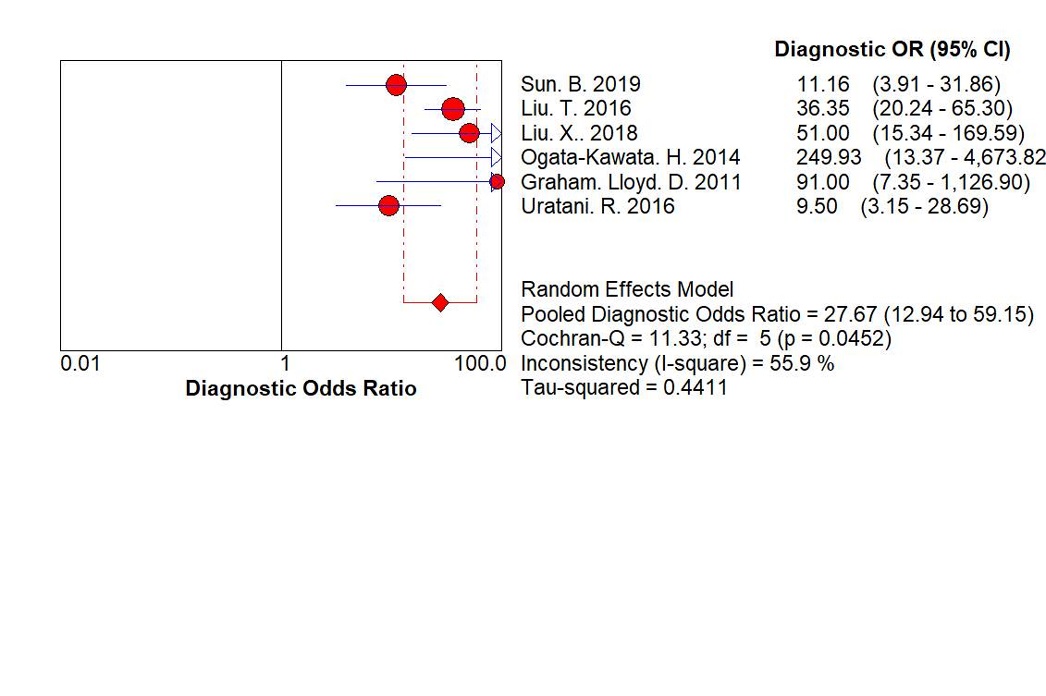


**E**


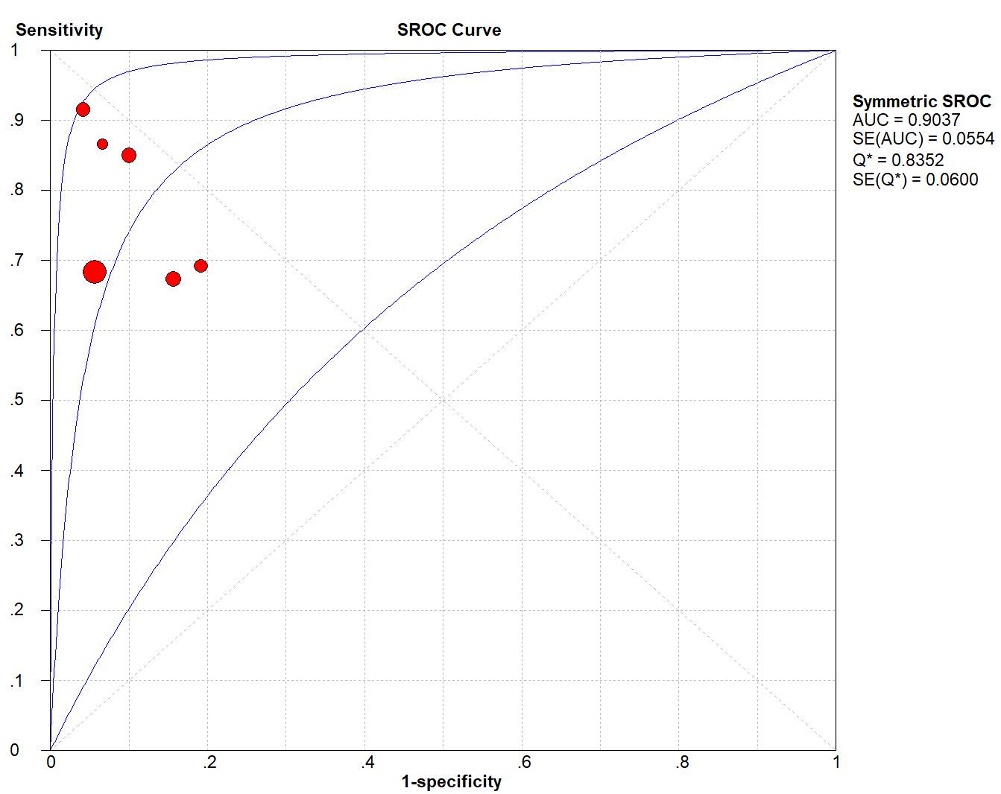


**F**

**Figure S4.** Forest plots of the diagnostic value for exosomes in detecting colorectal cancer. (**A**) Sensitivity. (**B**) Specificity. (**C**) positive likelihood ratio. (**D**) negative likelihood ratio. (**E**) Diagnostic odds ratio. (**F**) SROC curve.


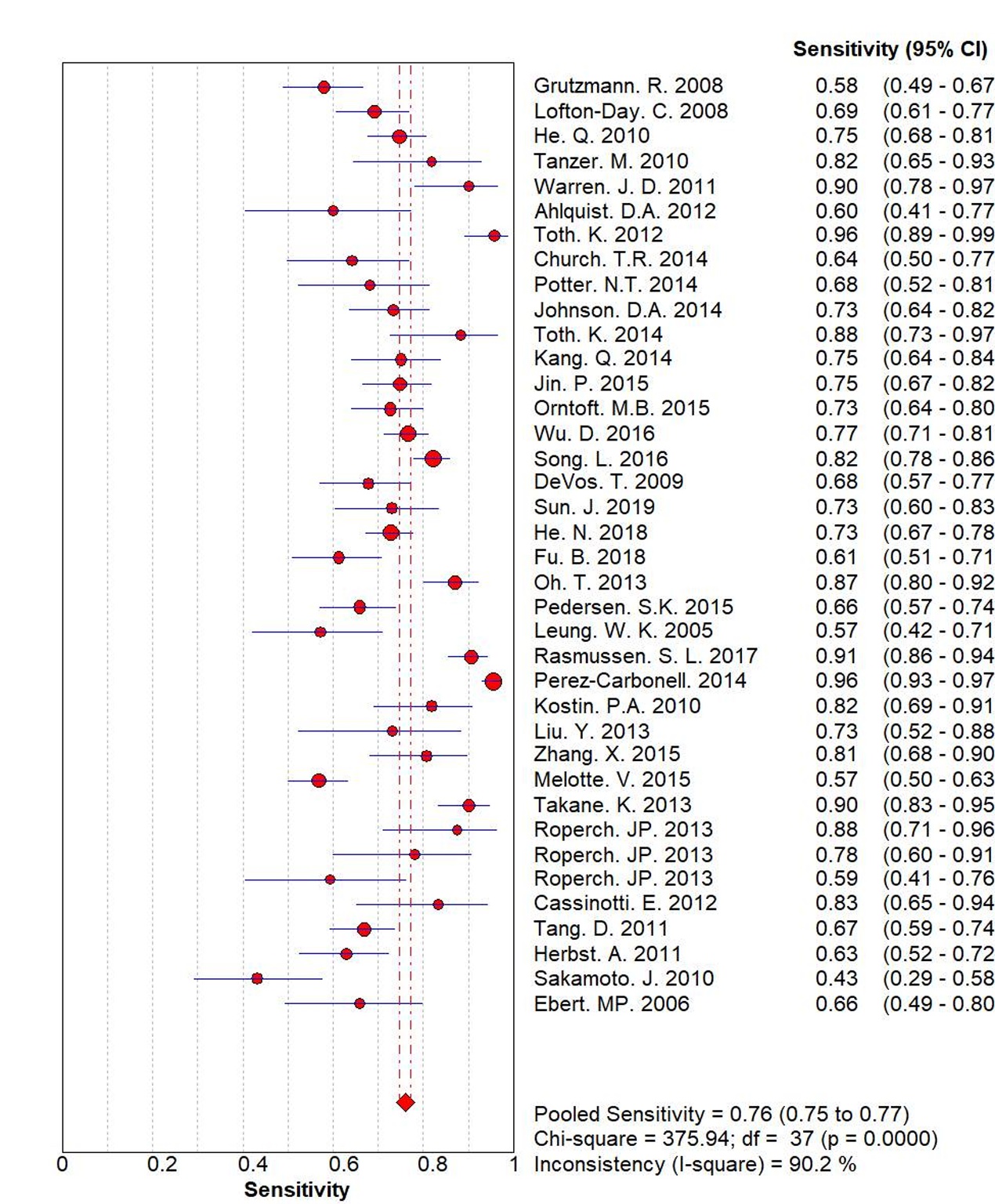

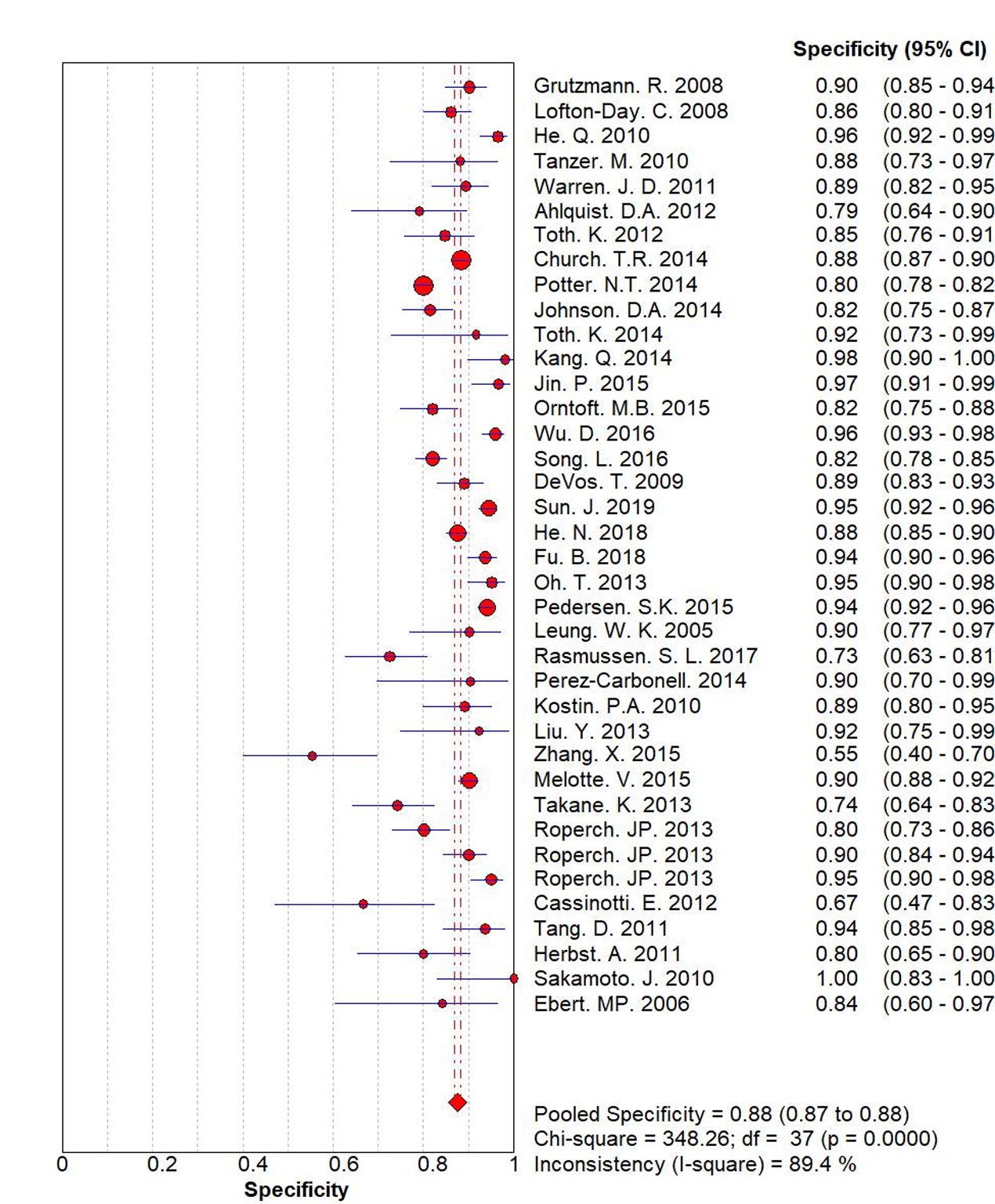

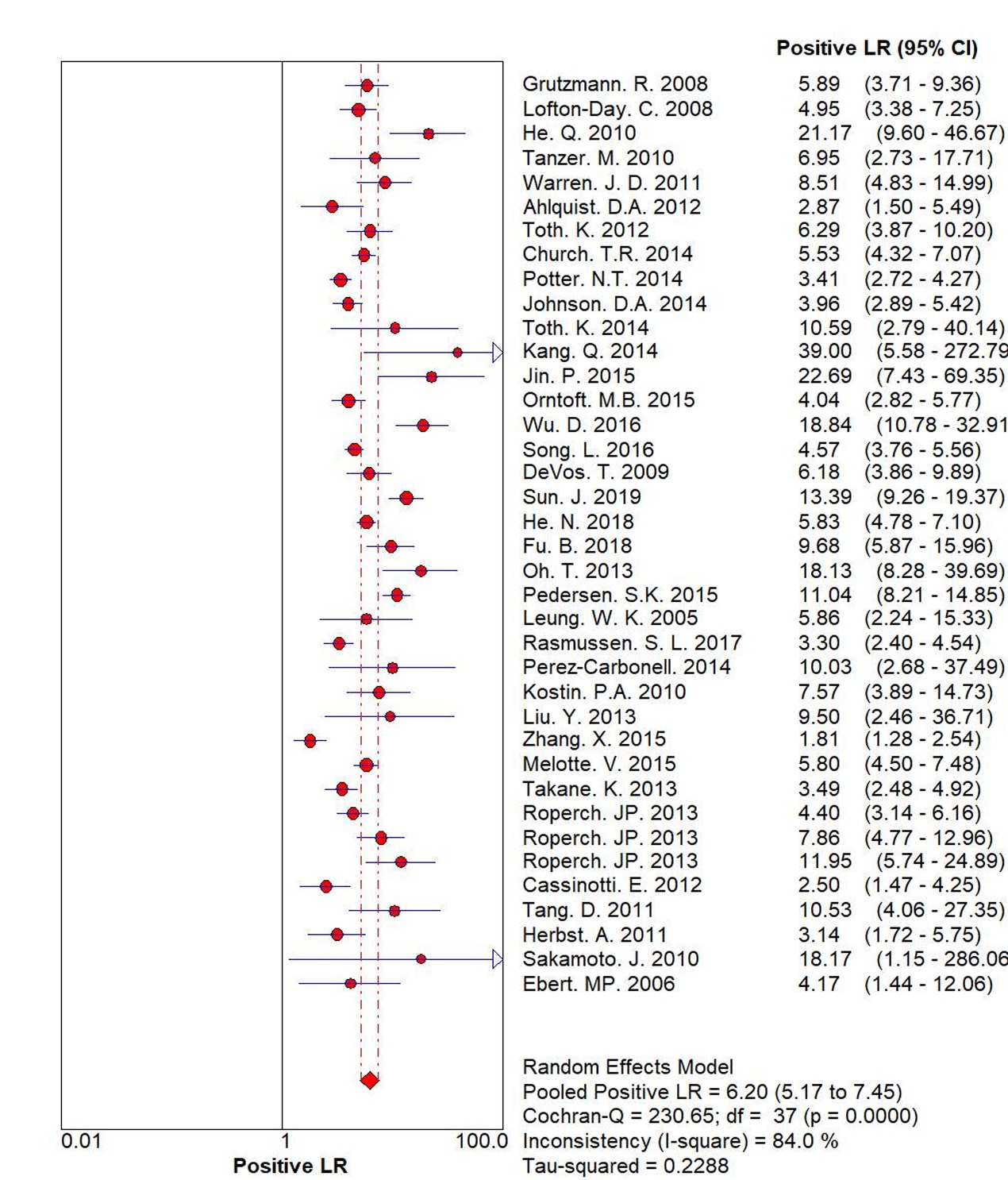

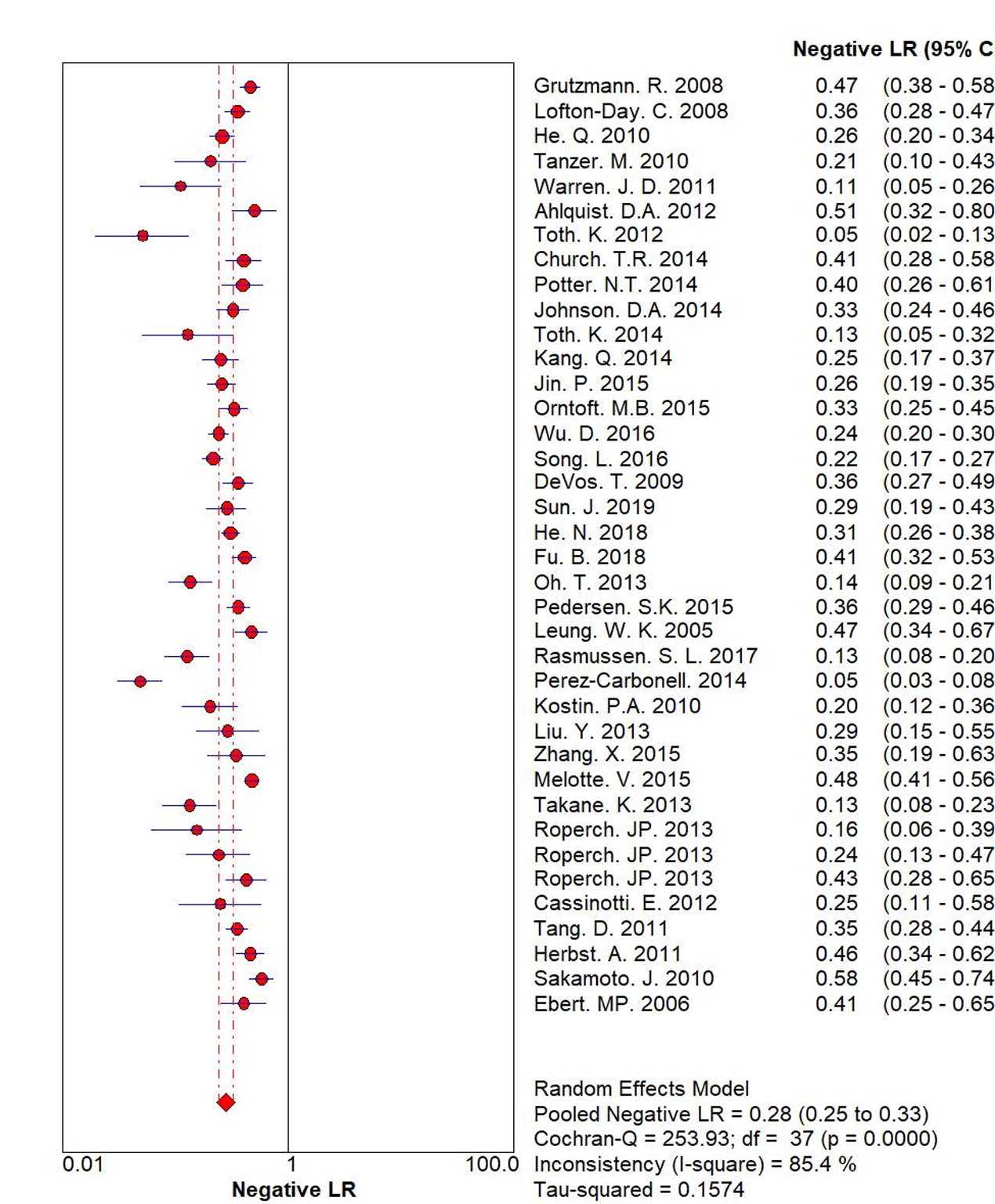


**A**

**B**

**C**

**D**


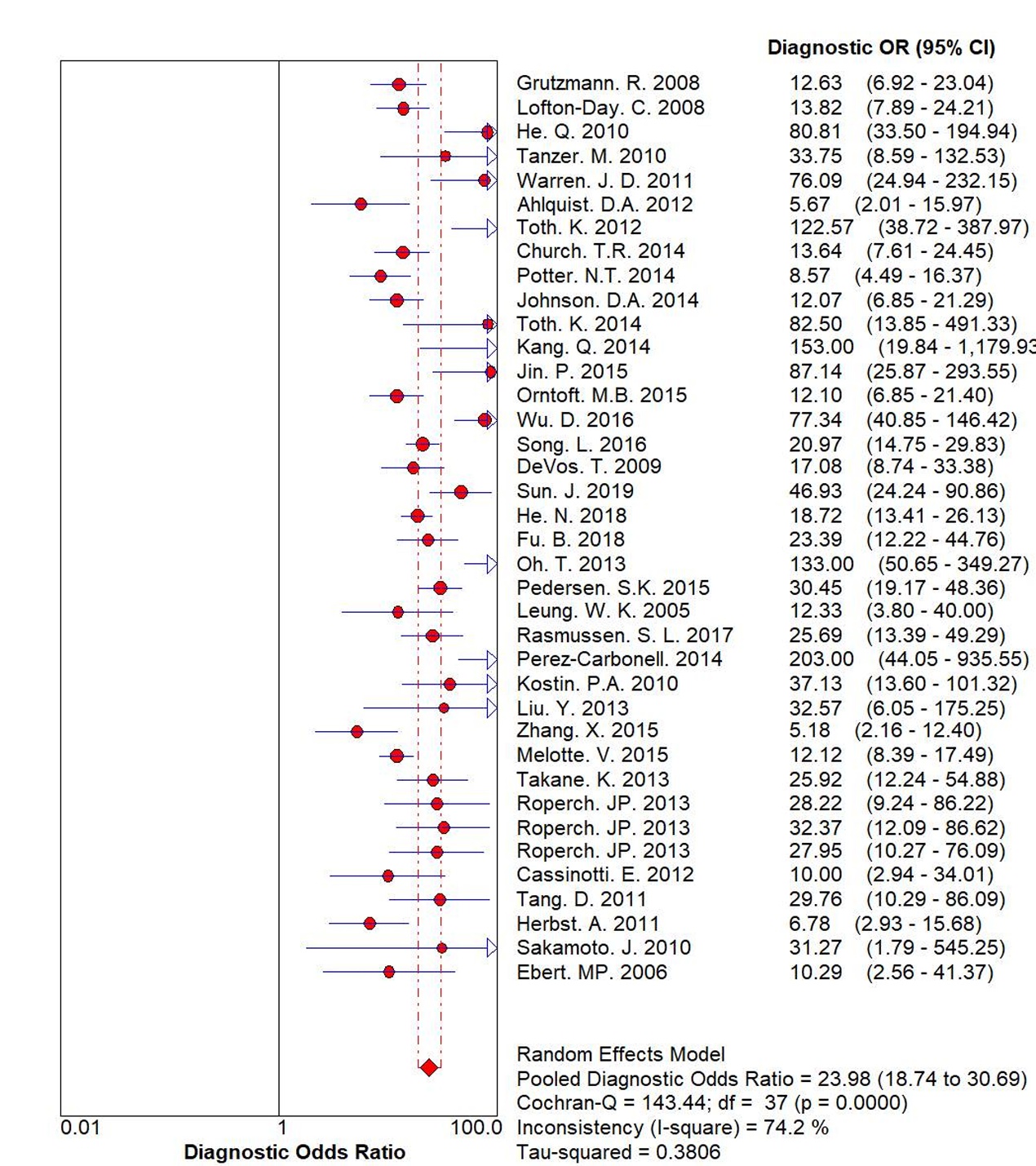


**D**

**E**


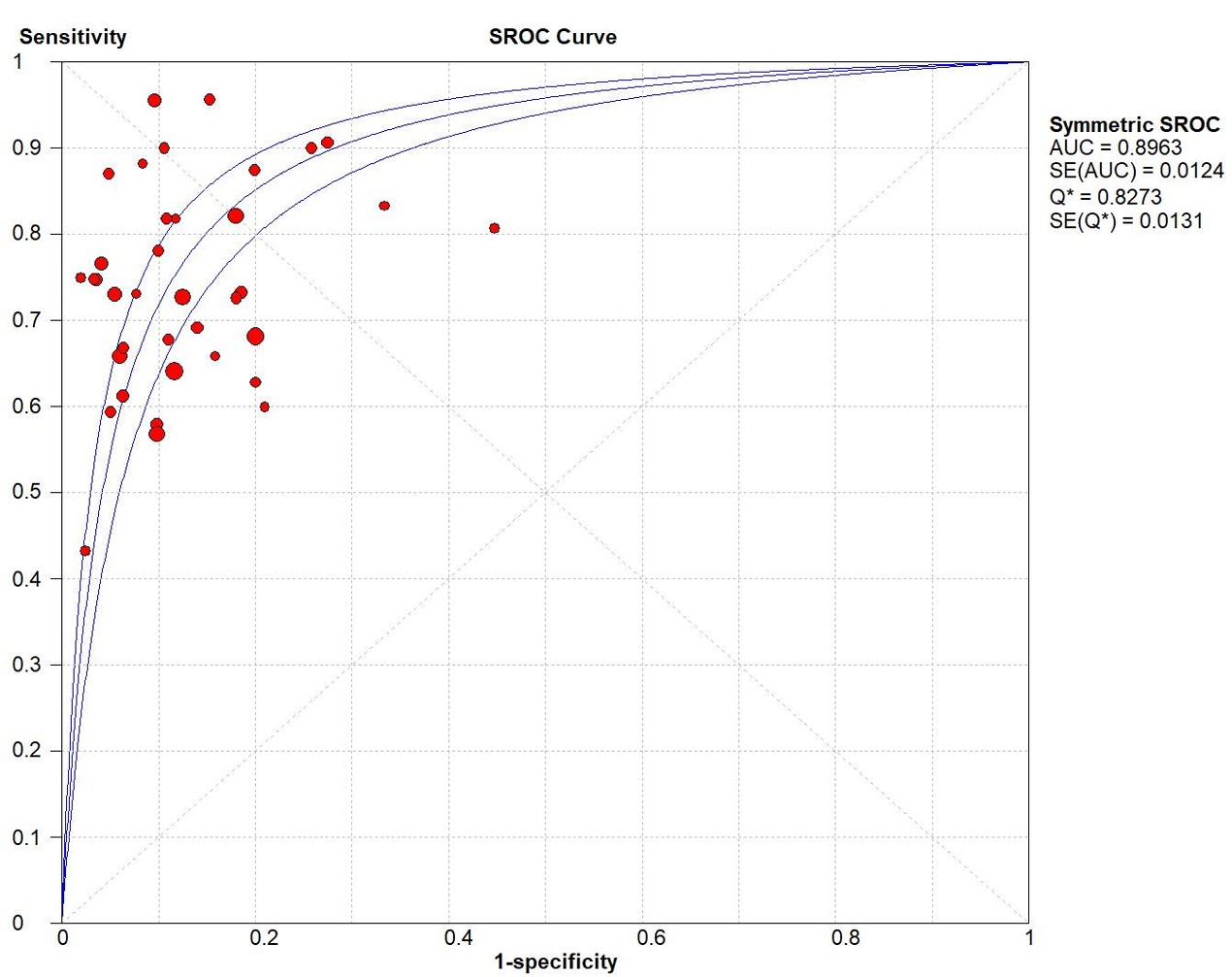


**F**

**Figure S5.** Forest plots of the diagnostic value for overall cfDNA in detecting colorectal cancer. (**A**) Sensitivity. (**B**) Specificity. (**C**) Positive likelihood ratio. (**D**) Negative likelihood ratio. (**E**) Diagnostic odds ratio. (**F**) SROC curve.


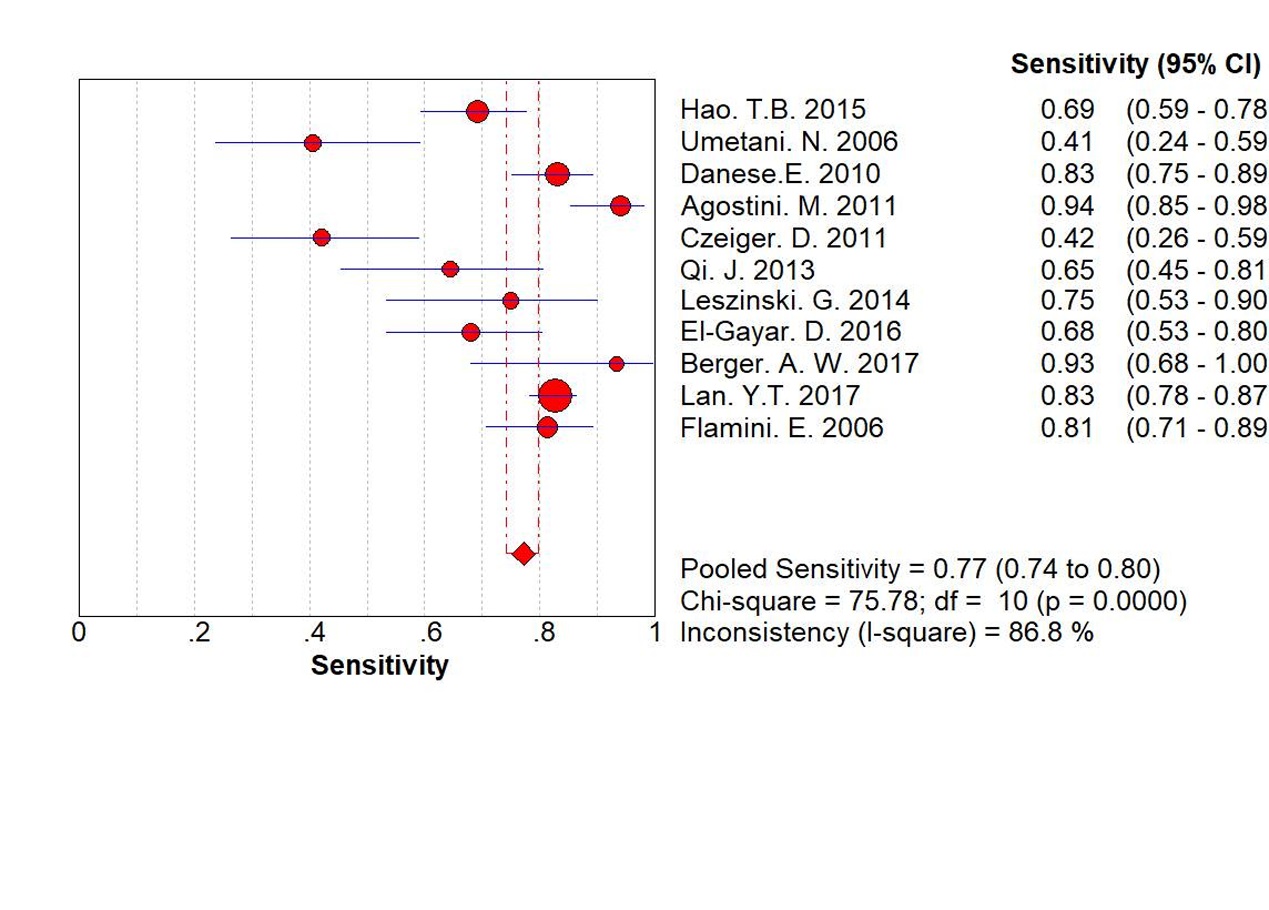

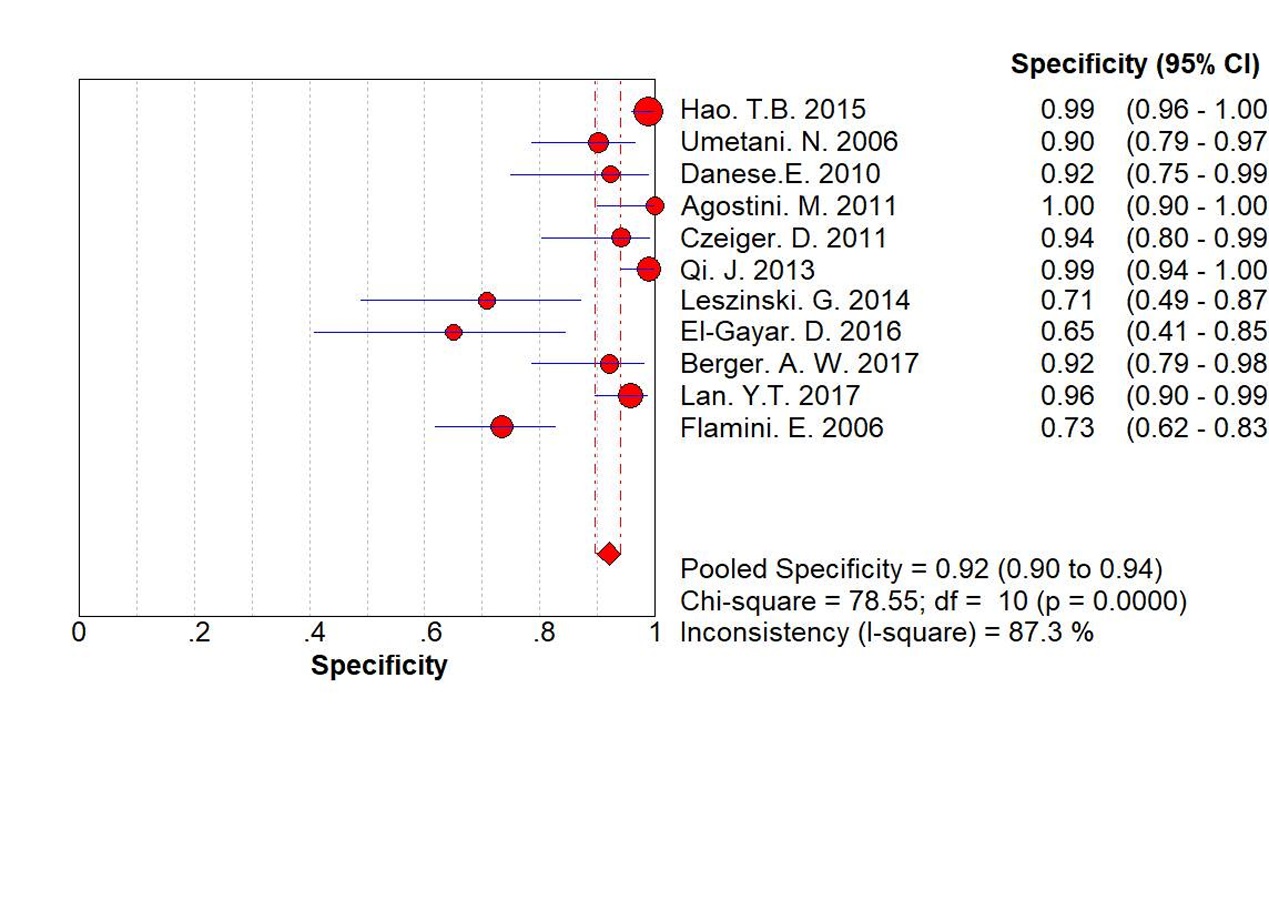

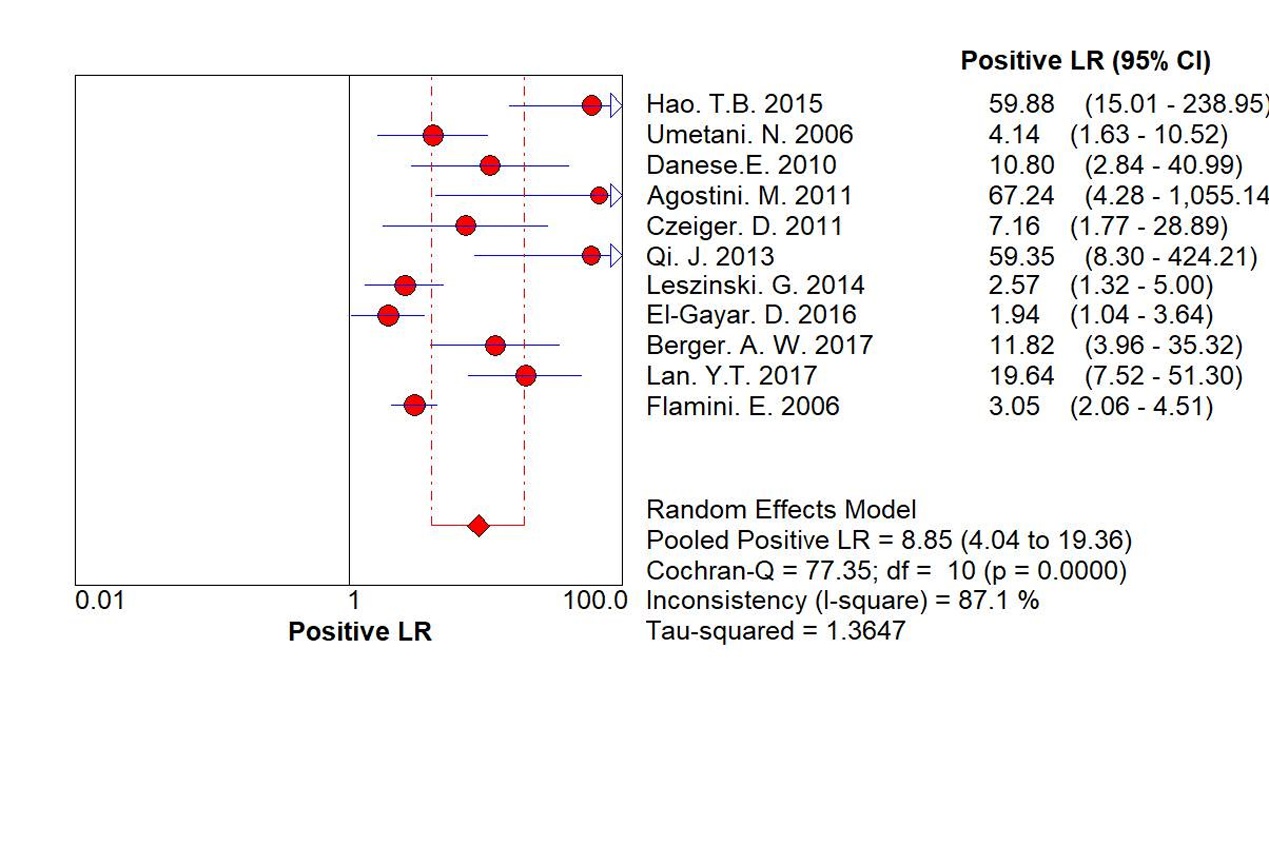


**B**

**A**

**C**


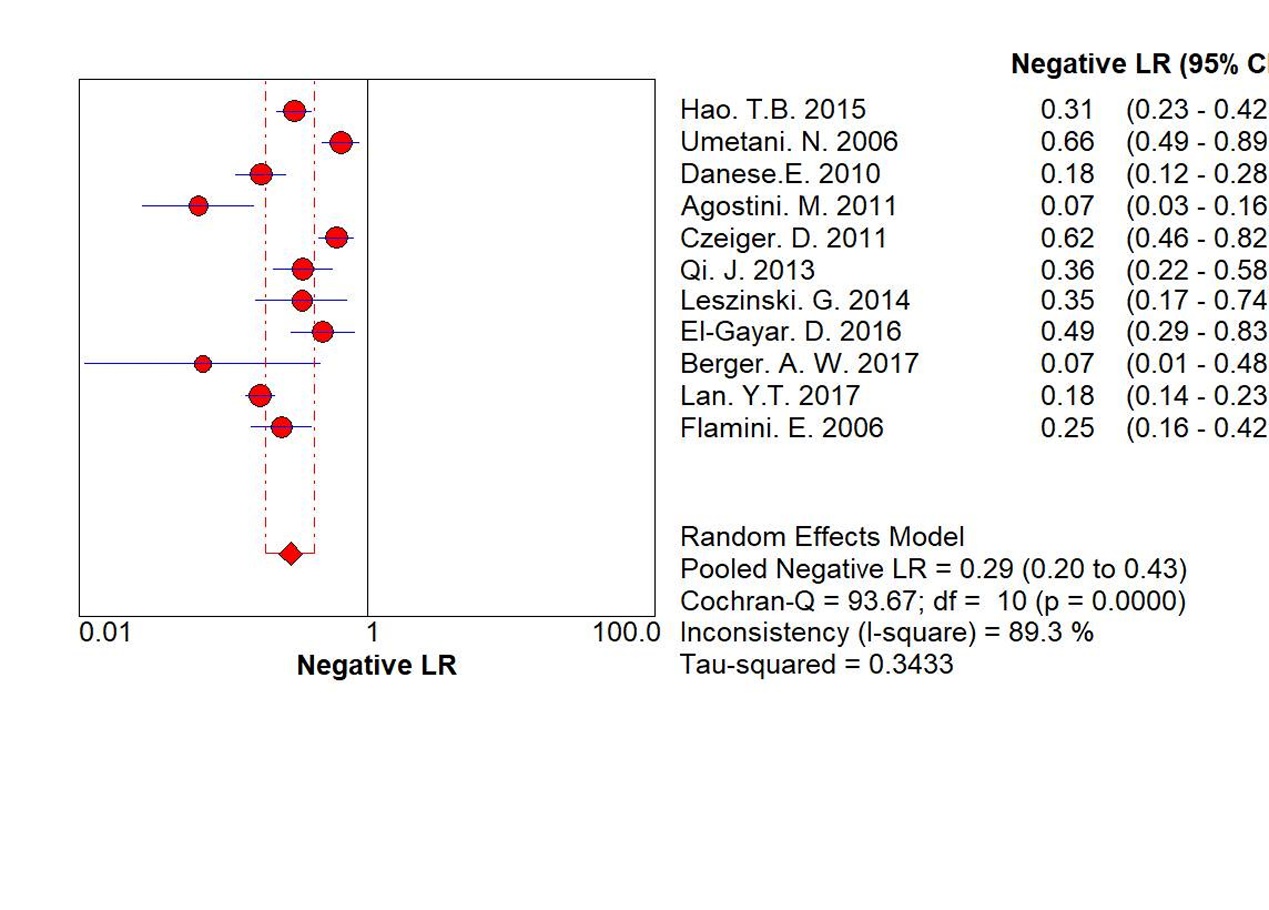

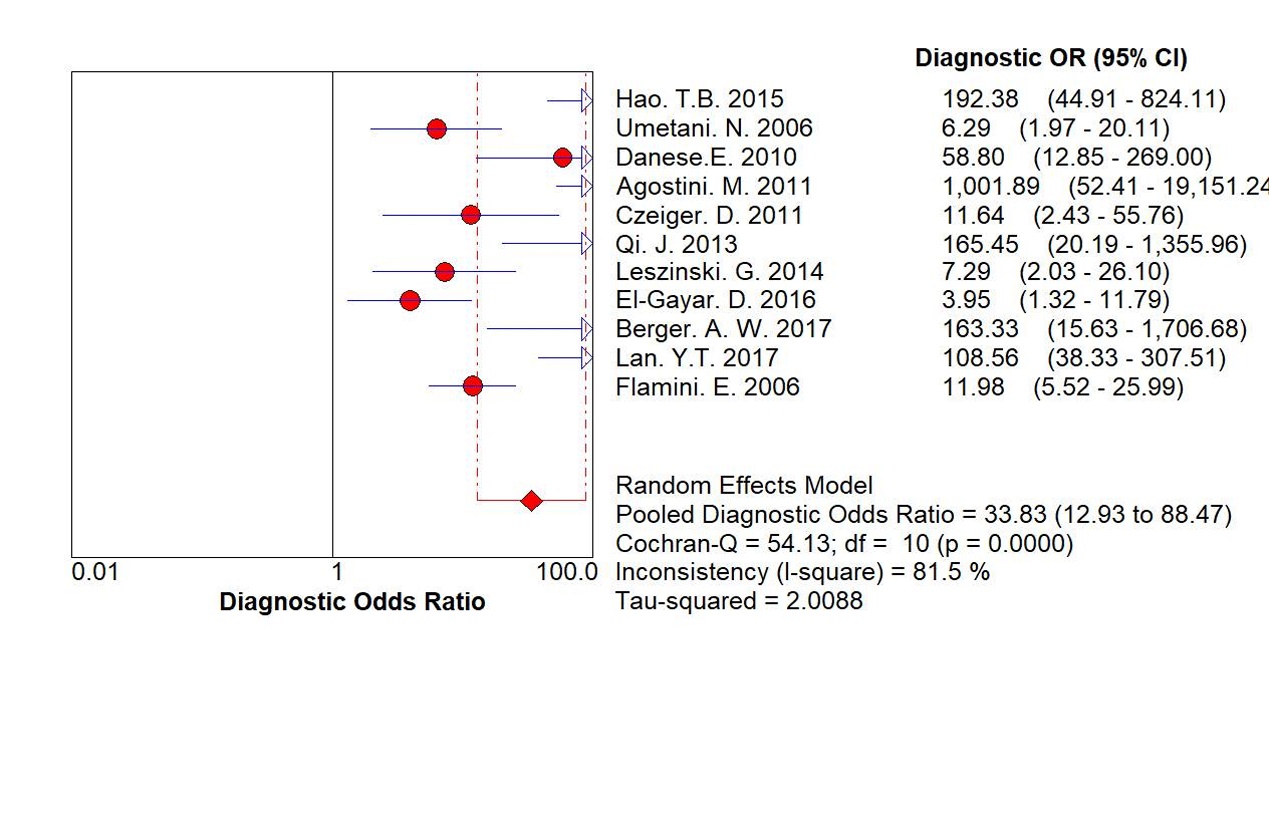


**D**

**E**


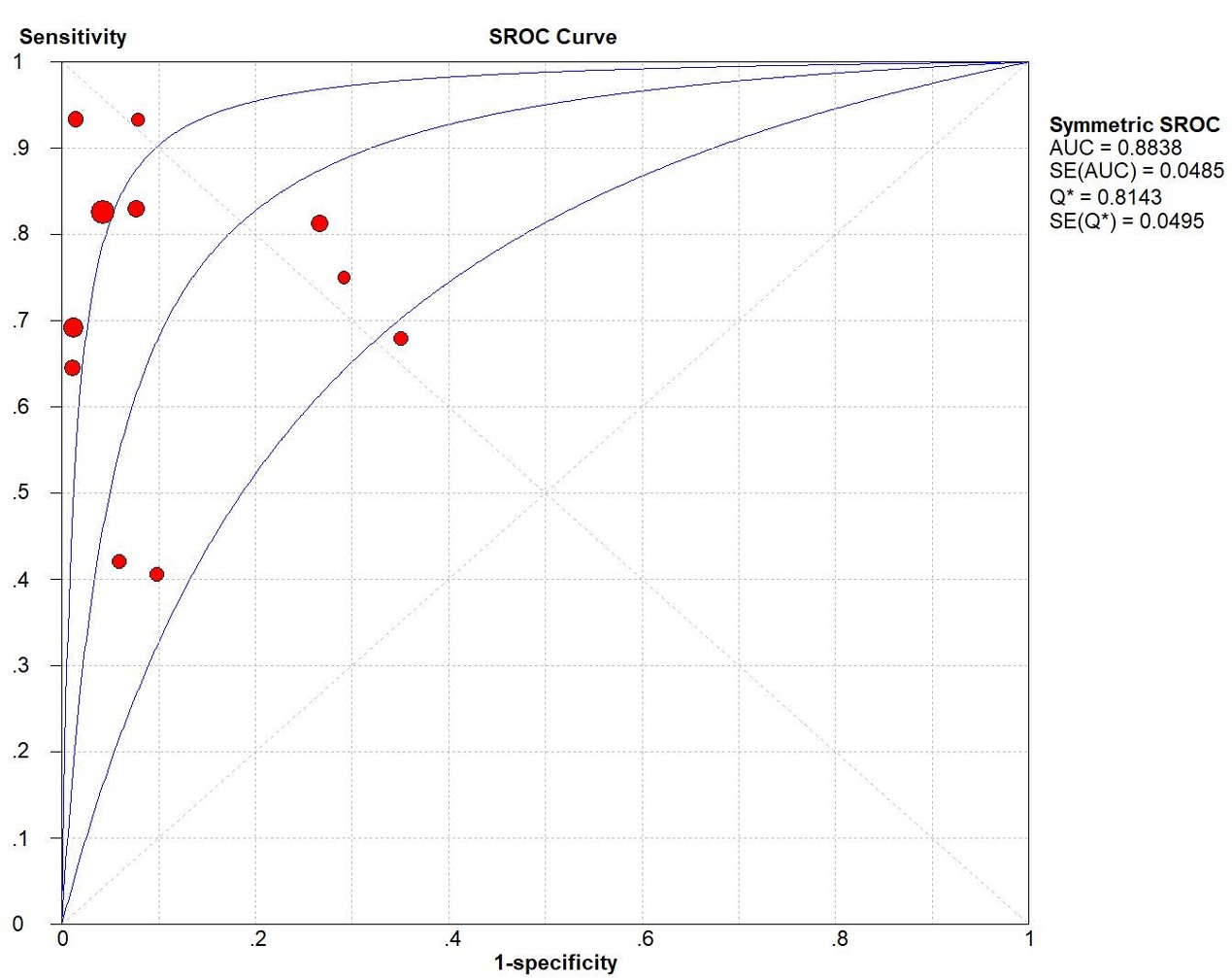


**F**

**Figure S6.** Forest plots of the diagnostic value for cell-free DNA level (CFD level) in detecting colorectal cancer. (**A**) Sensitivity. (**B**) Specificity. (**C**) positive likelihood ratio. (**D**) negative likelihood ratio. (**E**) Diagnostic odds ratio. (**F**) SROC curve.


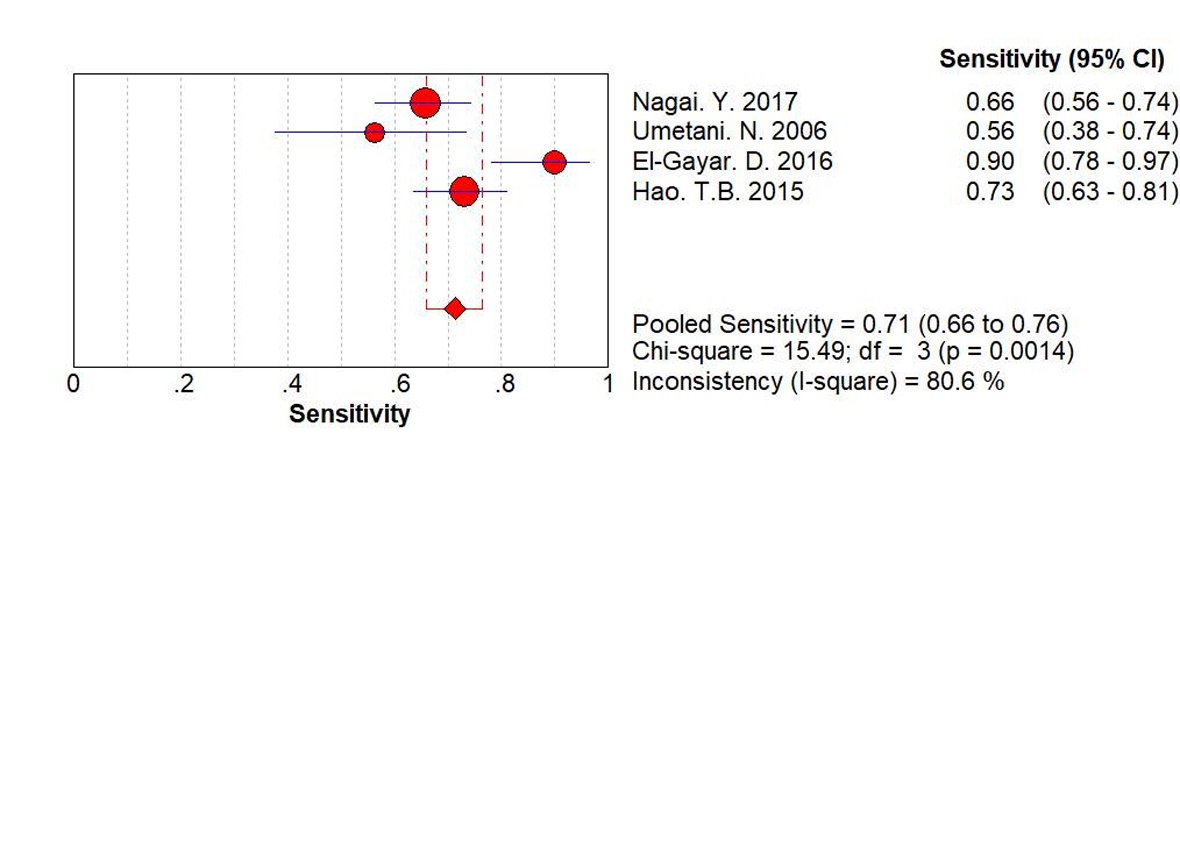


**A**


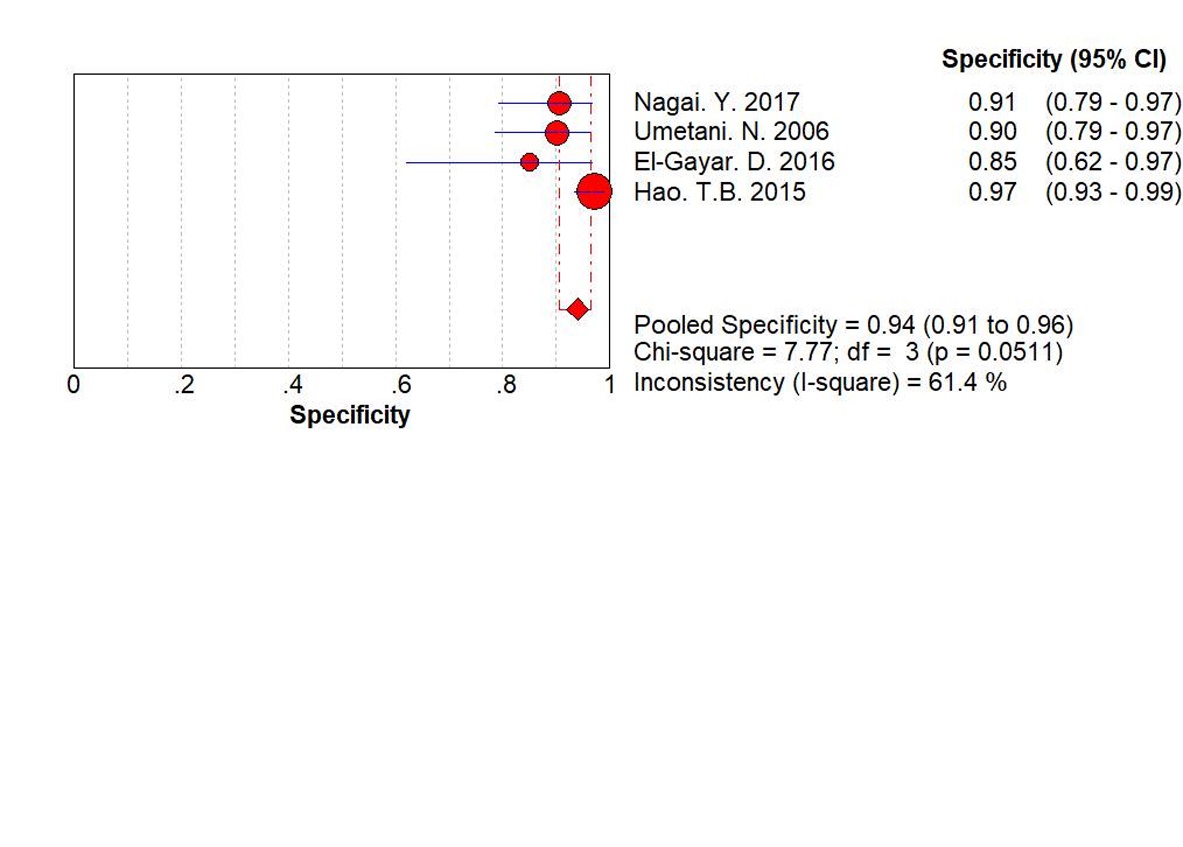


**B**


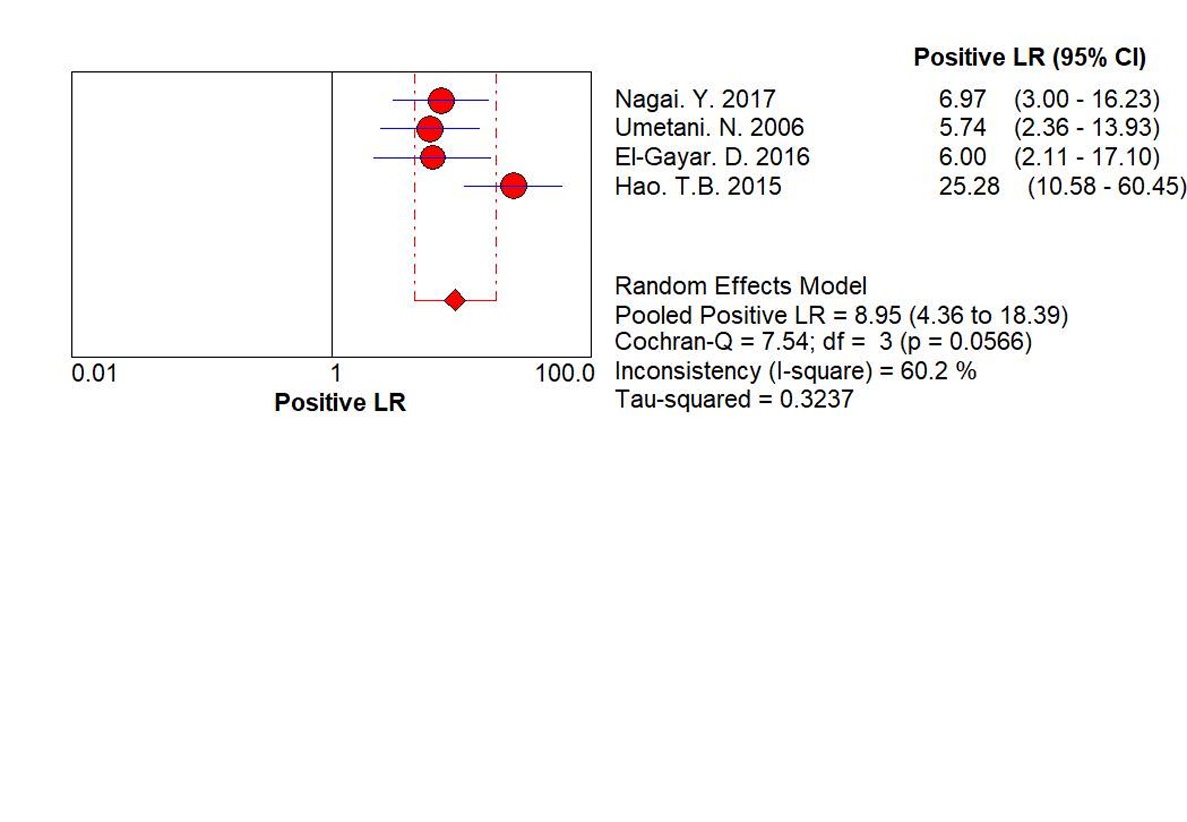

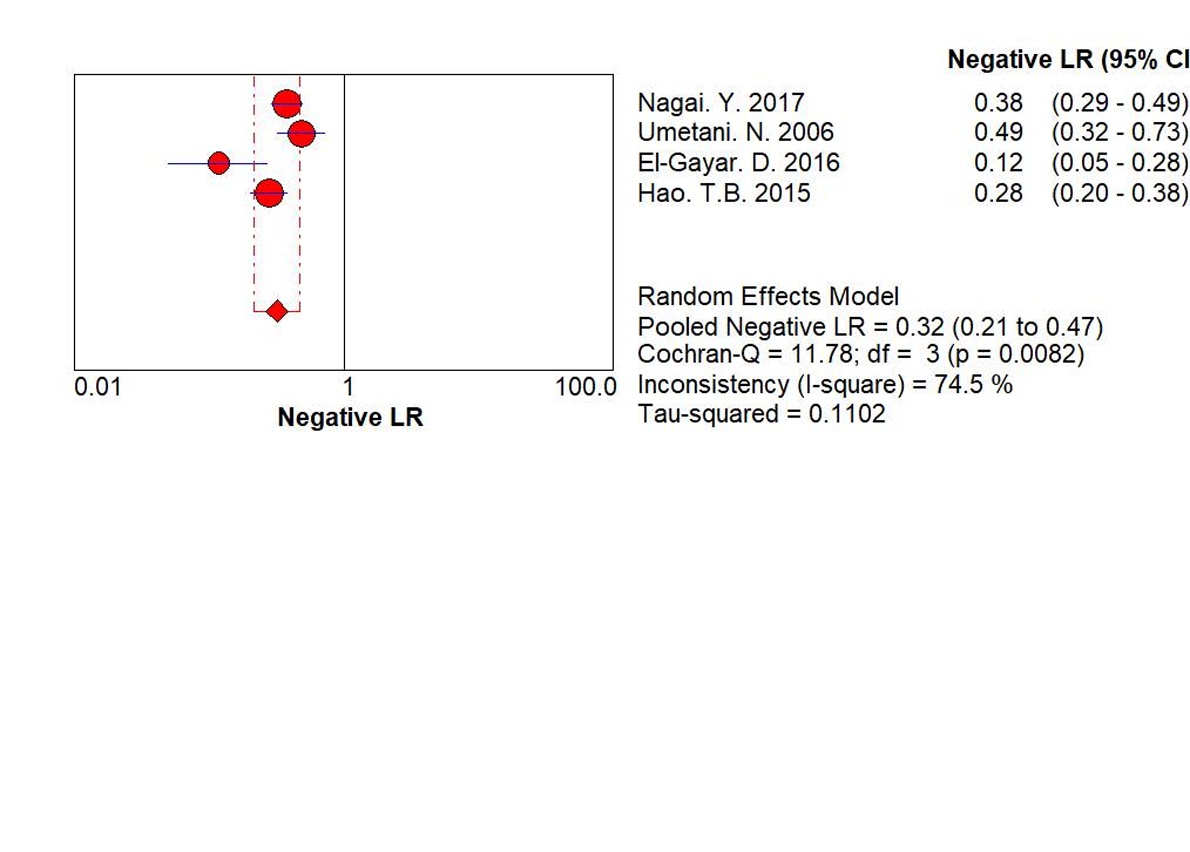

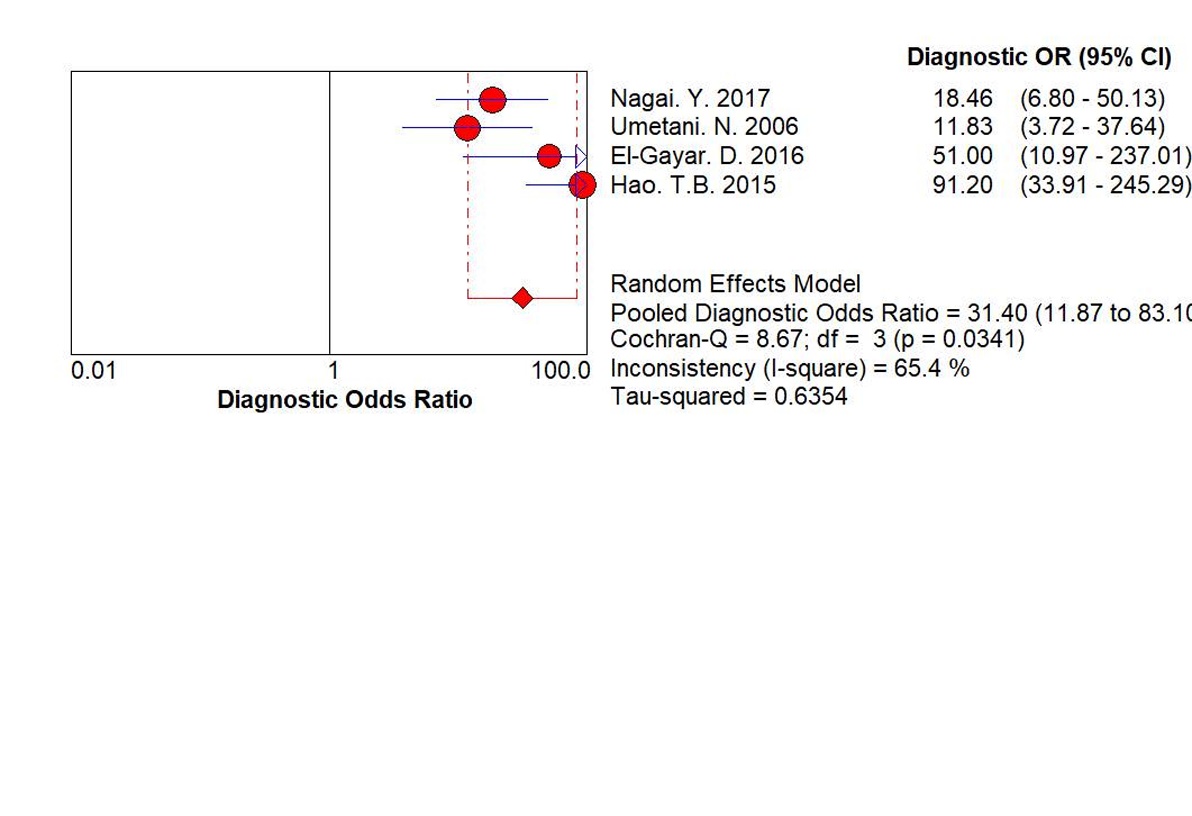


**C**

**D**

**E**


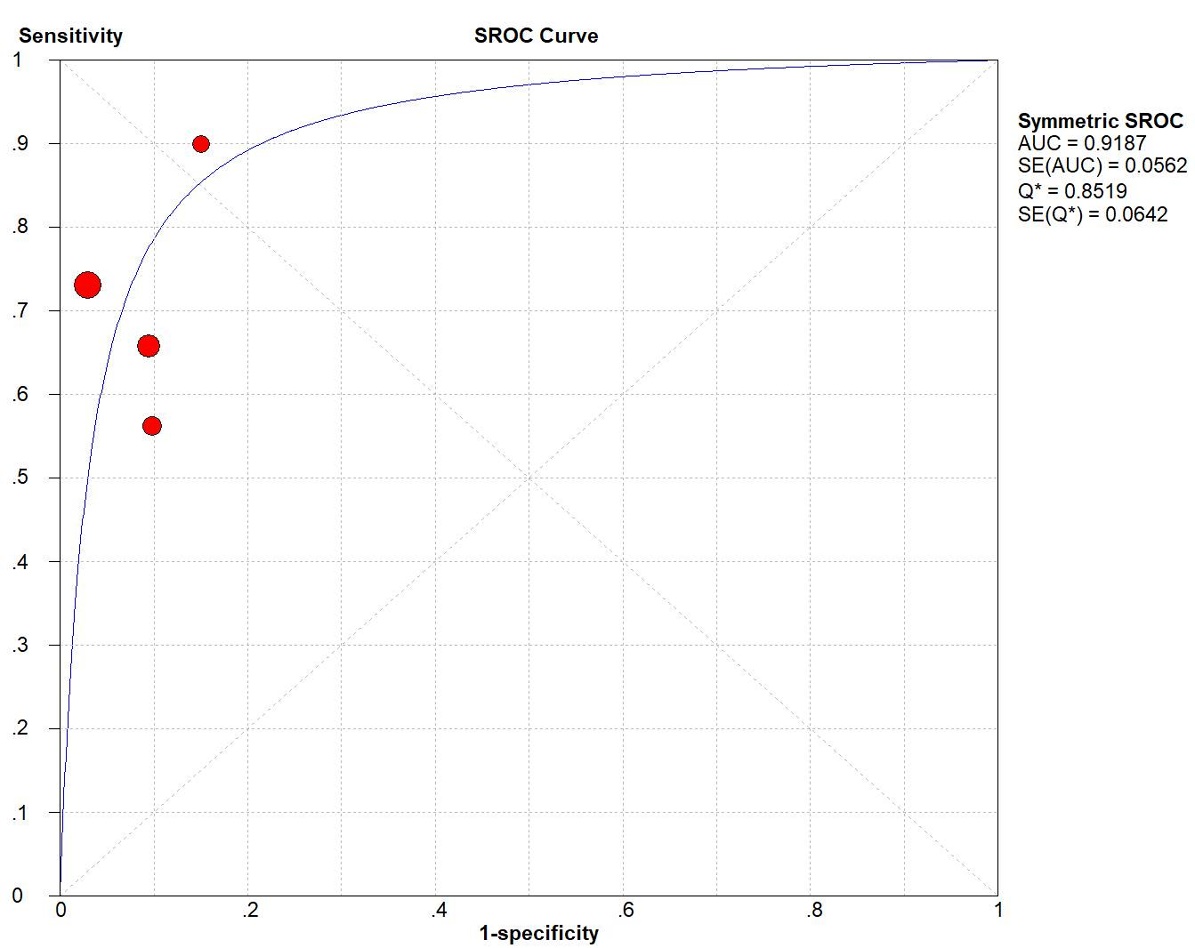


**F**

**Figure S7.** Forest plots of the diagnostic value for cell-free DNA integrity in detecting colorectal cancer. (**A**) Sensitivity. (**B**) Specificity. (**C**) positive likelihood ratio. (**D**) negative likelihood ratio. (**E**) Diagnostic odds ratio. (**F**) SROC curve.


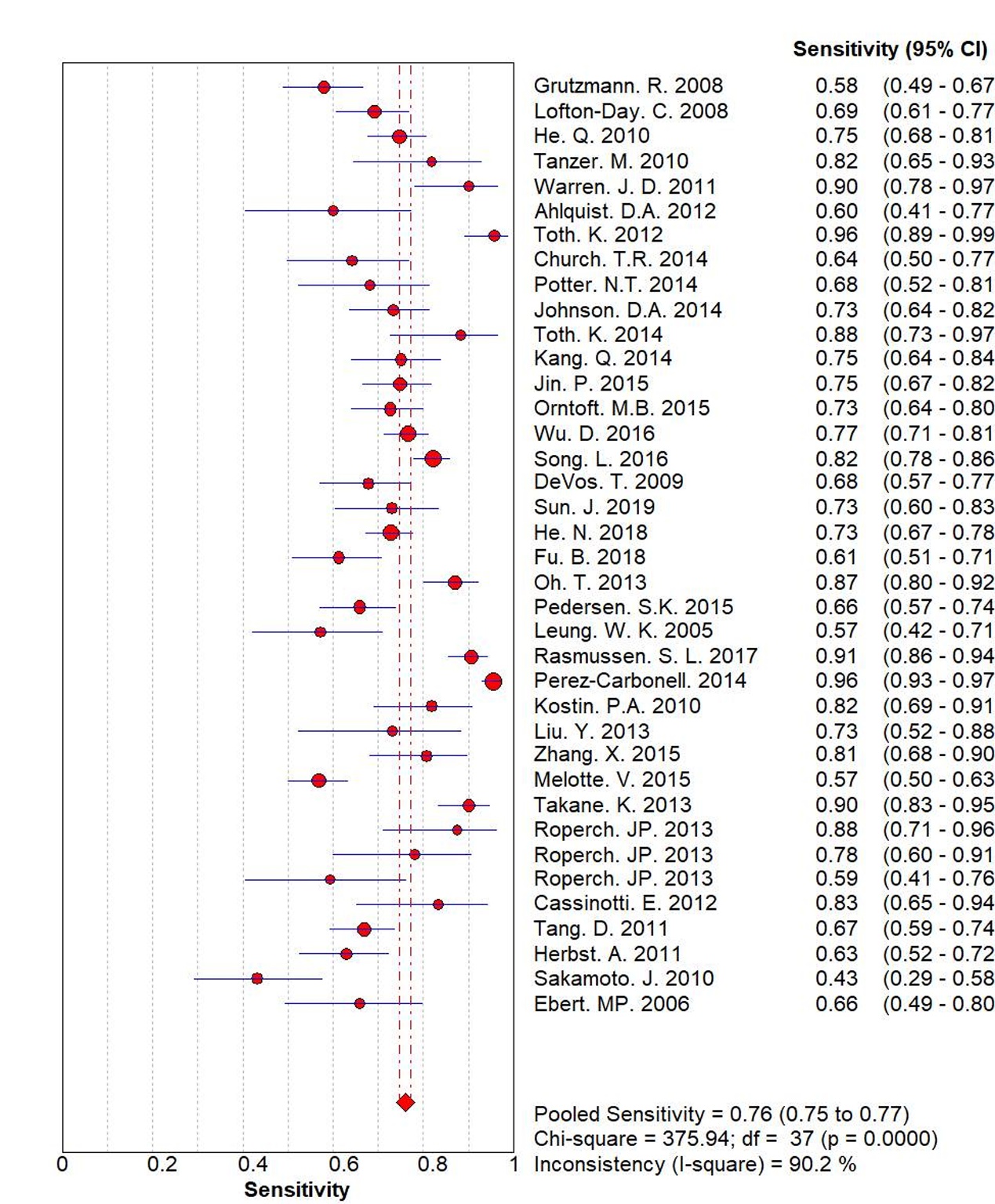

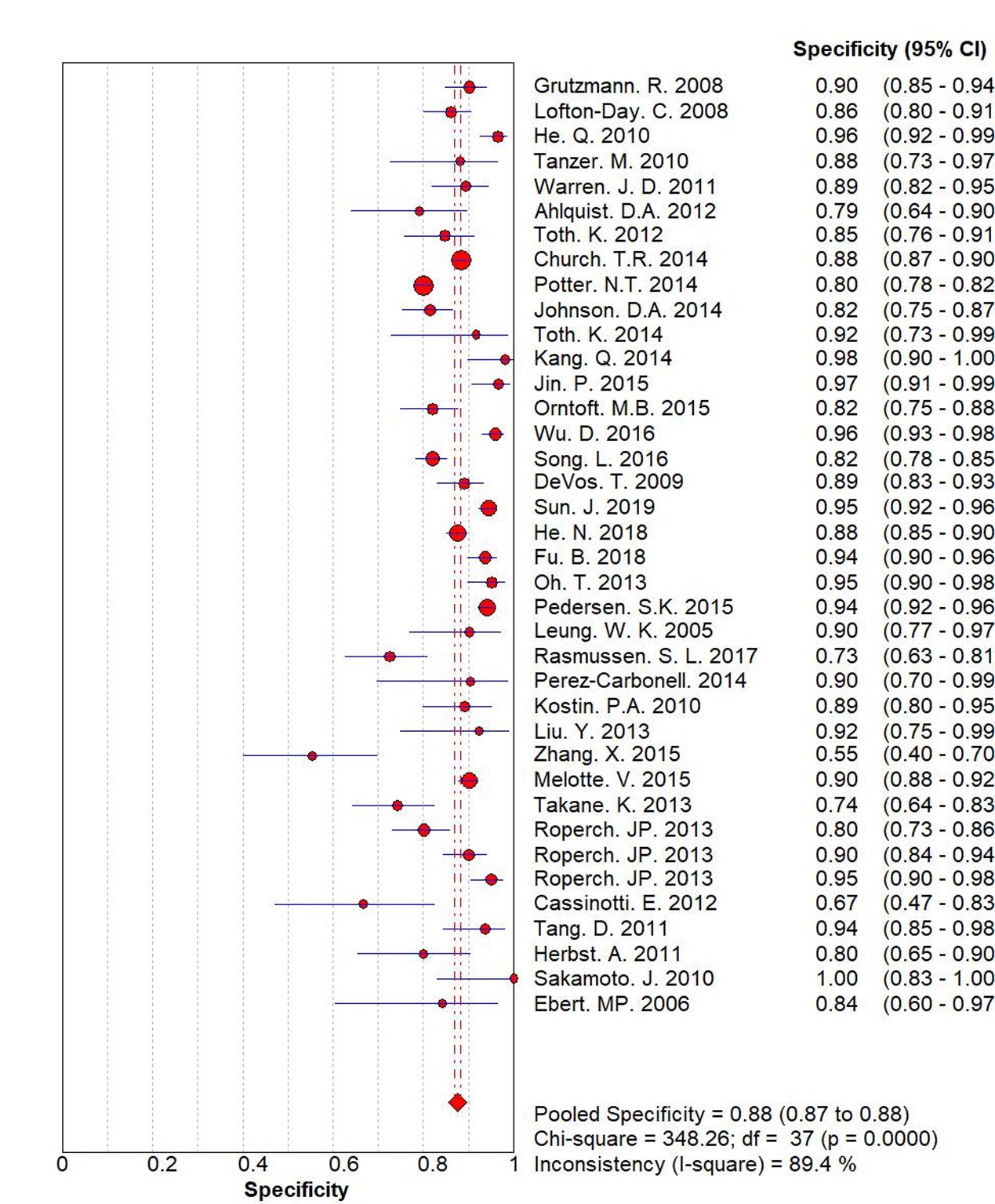


**A**

**B**

**B**

**C**


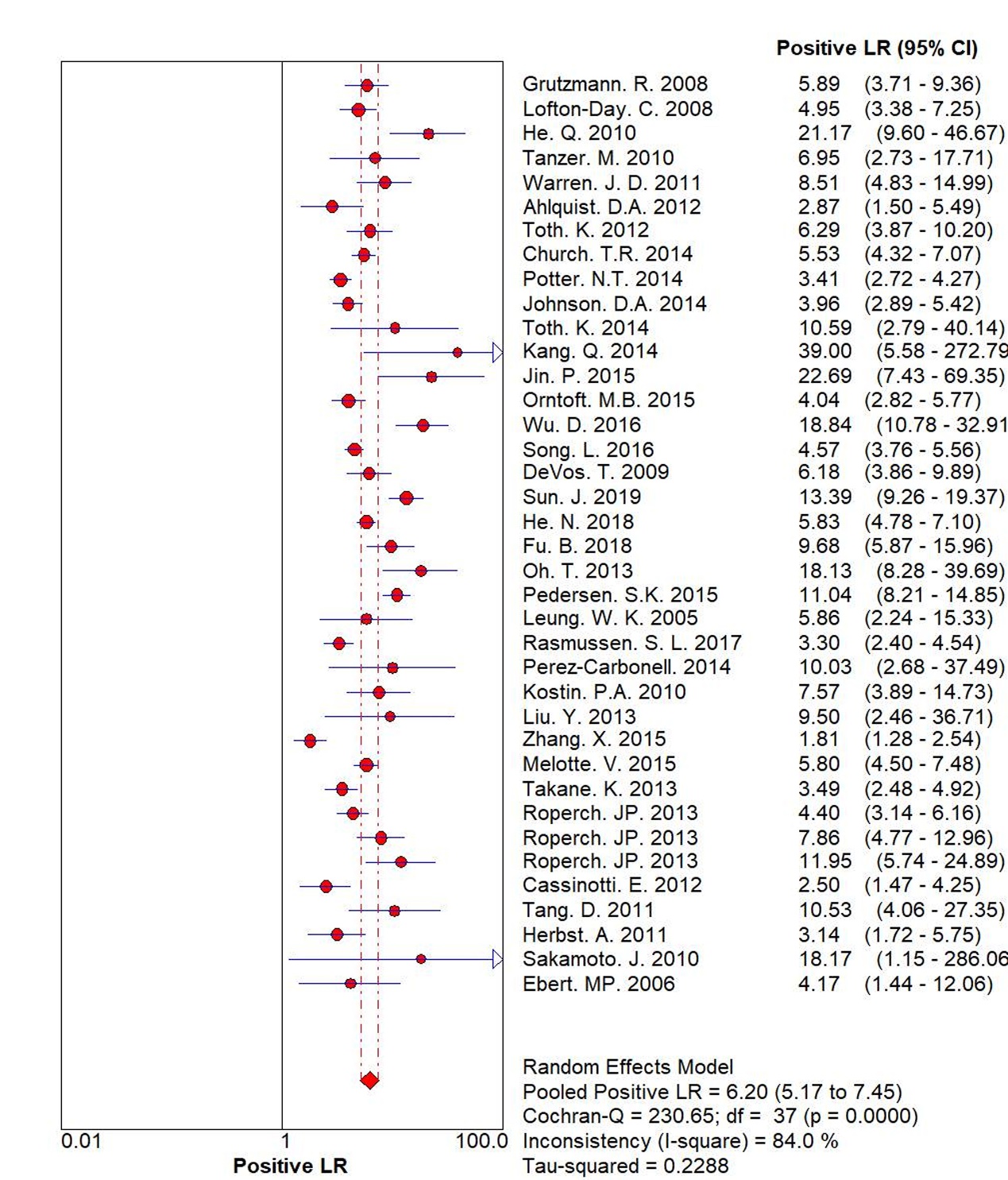

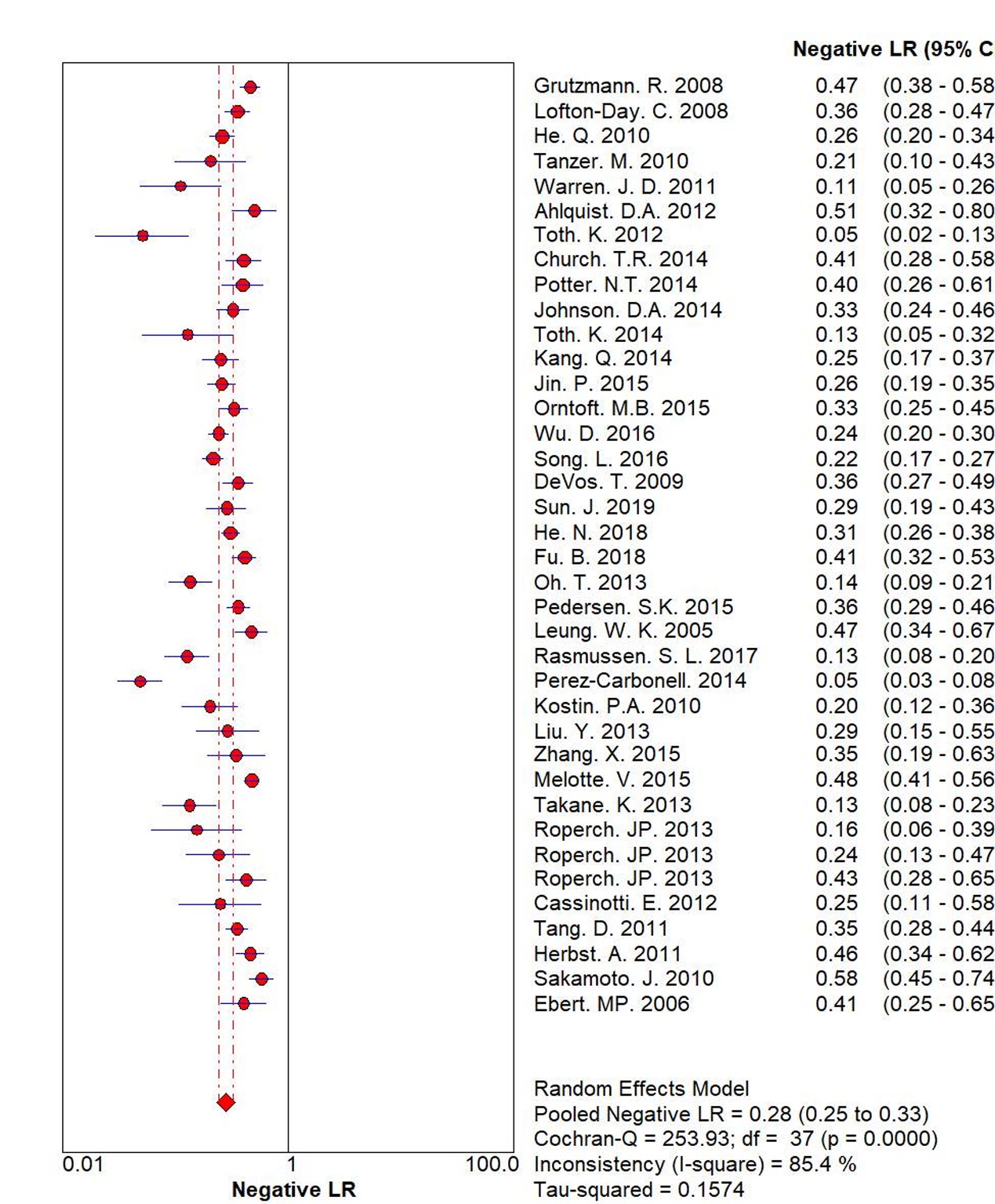

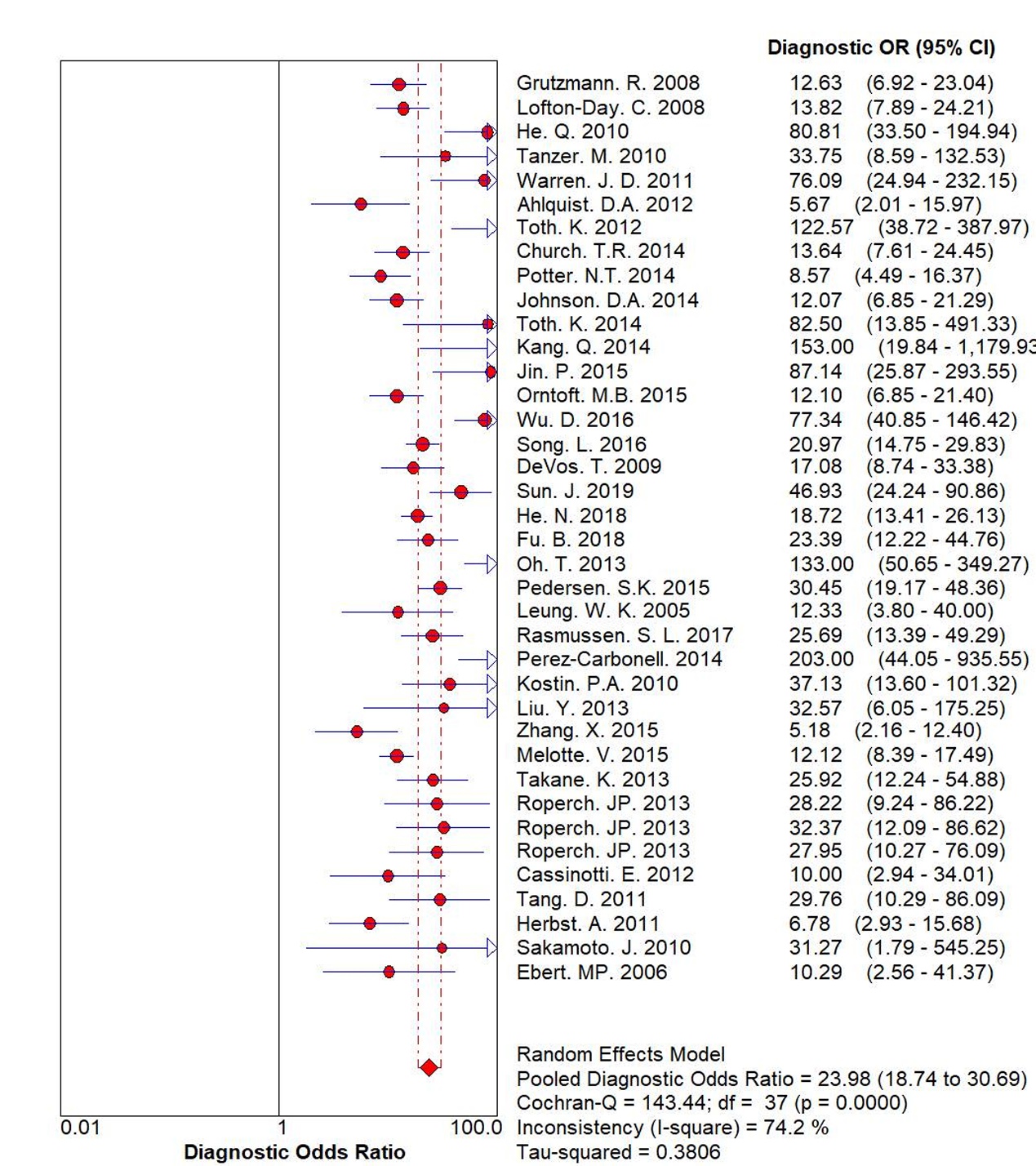

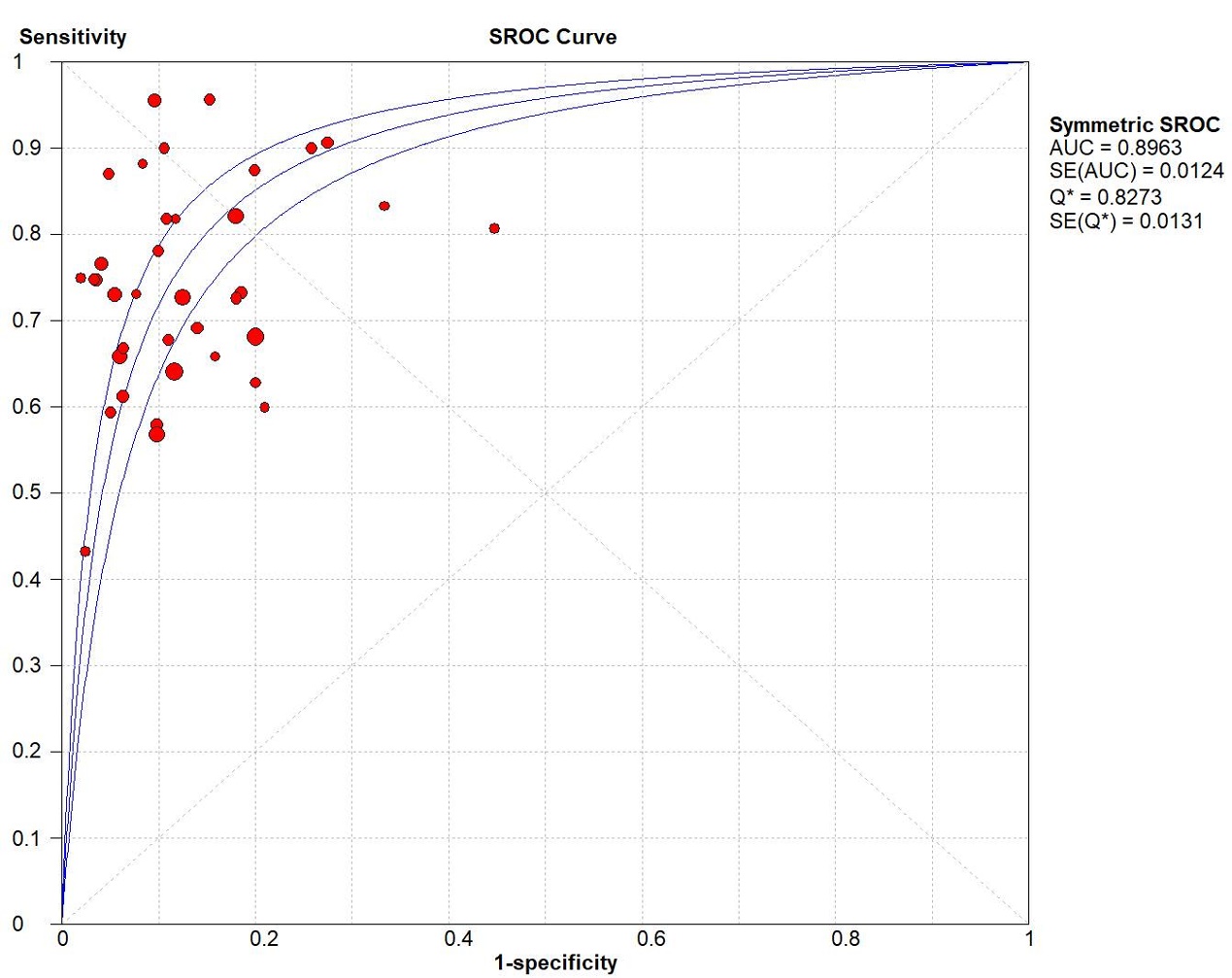


**C**

**D**

**E**

**F**

**F**

**Figure S8.** Forest plots of the diagnostic value for overall cfDNA methylation in detecting colorectal cancer. (**A**) Sensitivity. (**B**) Specificity. (**C**) positive likelihood ratio. (**D**) negative likelihood ratio. (**E**) Diagnostic odds ratio. (**F**) SROC curve.


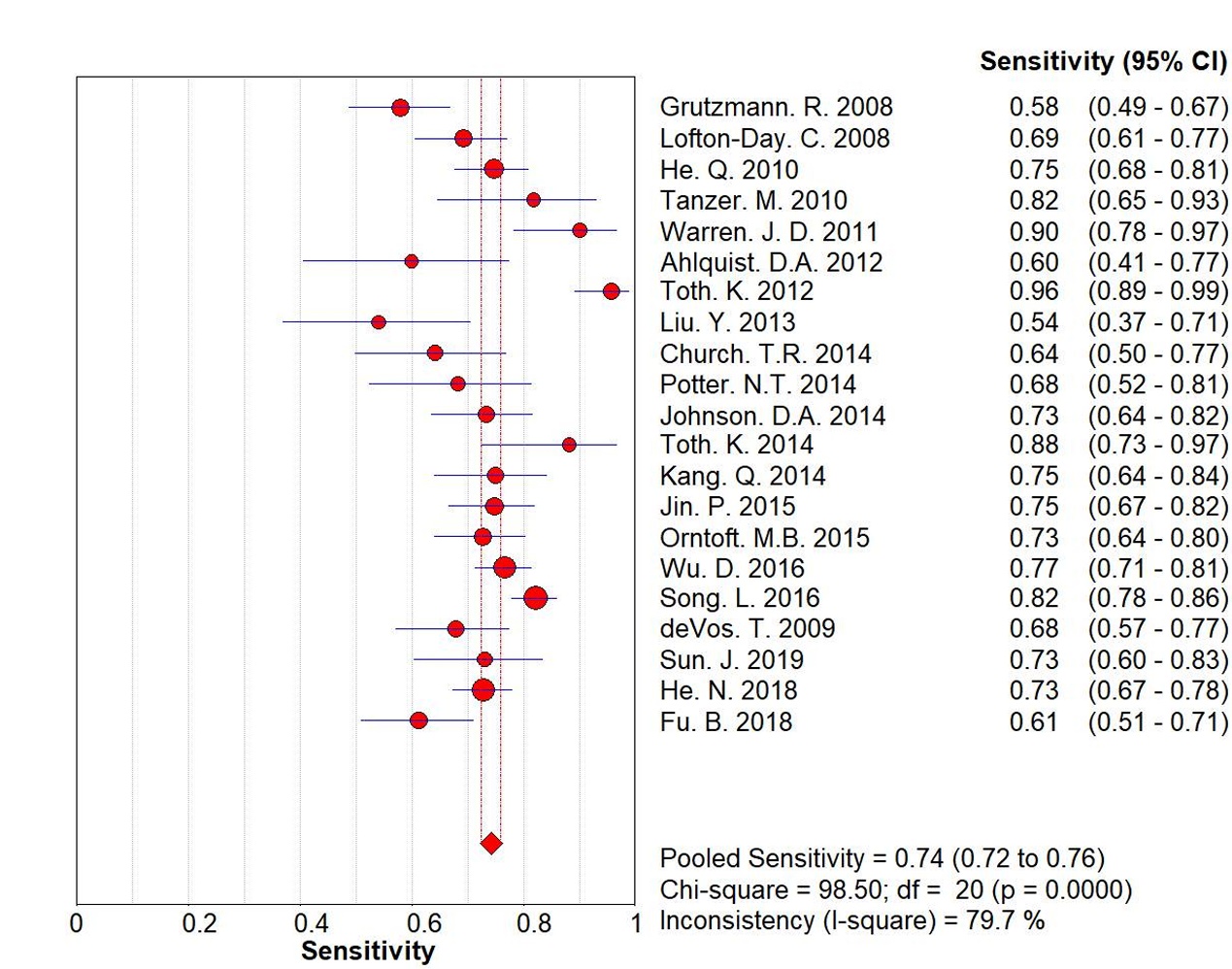

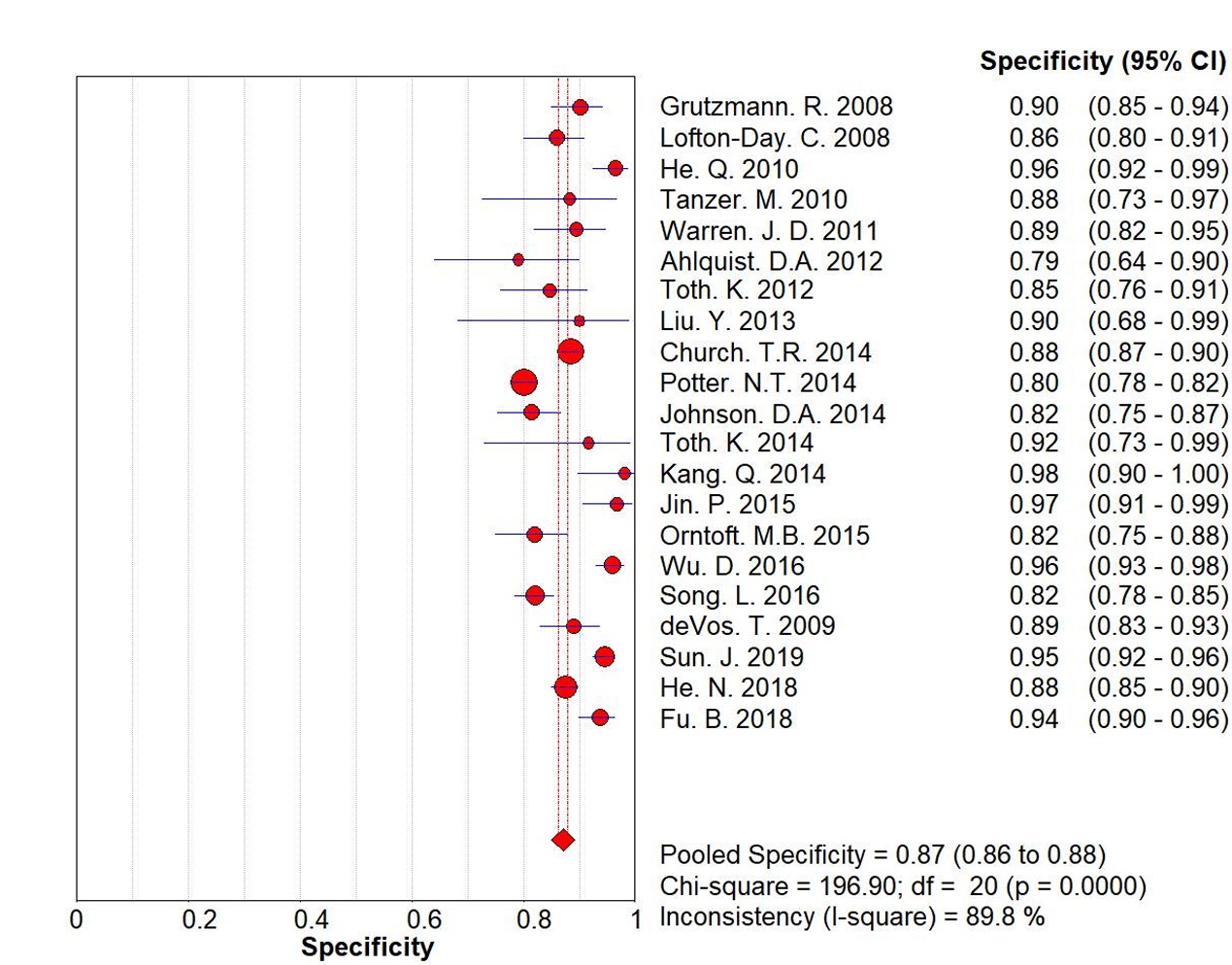


**B**

**A**


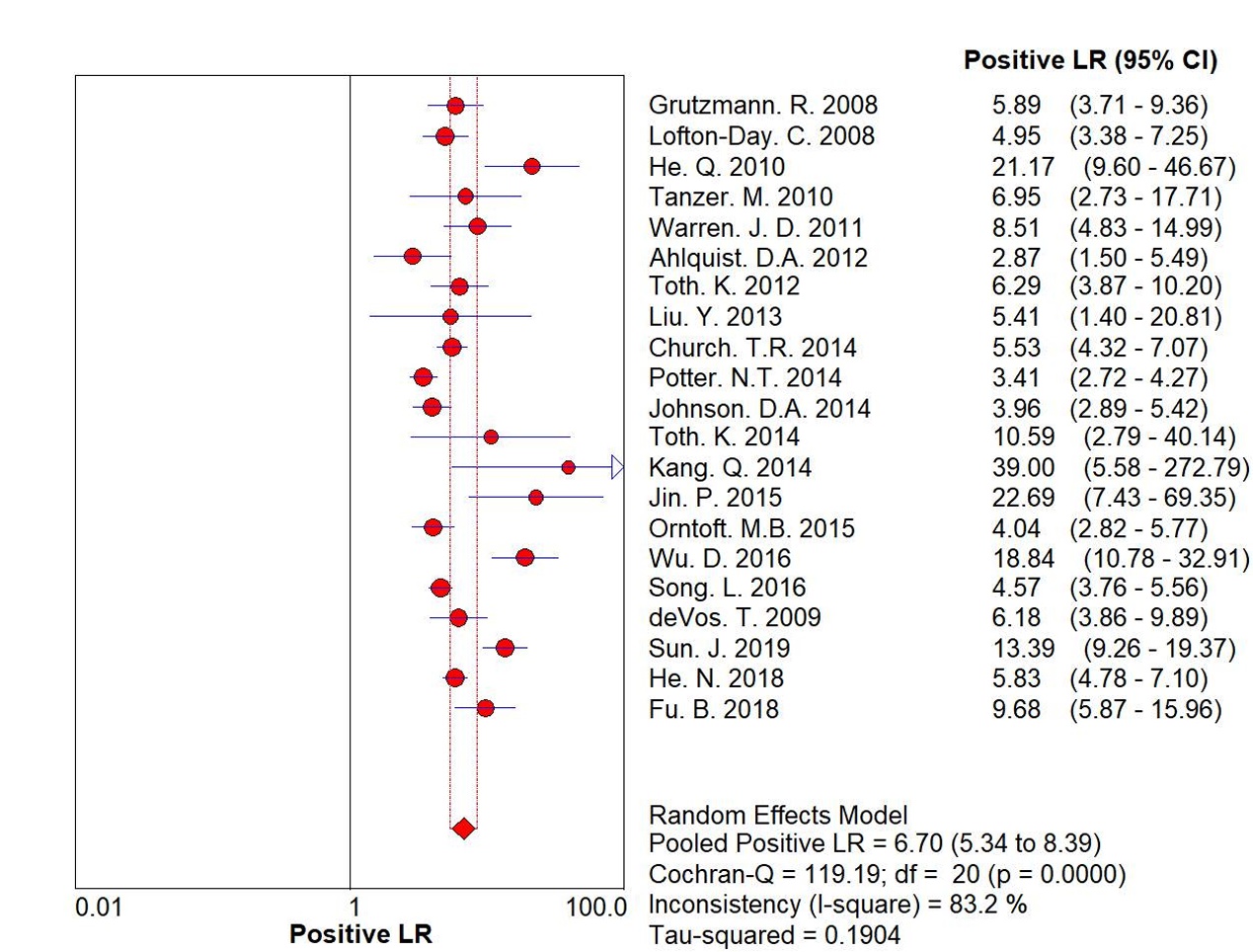


**C**


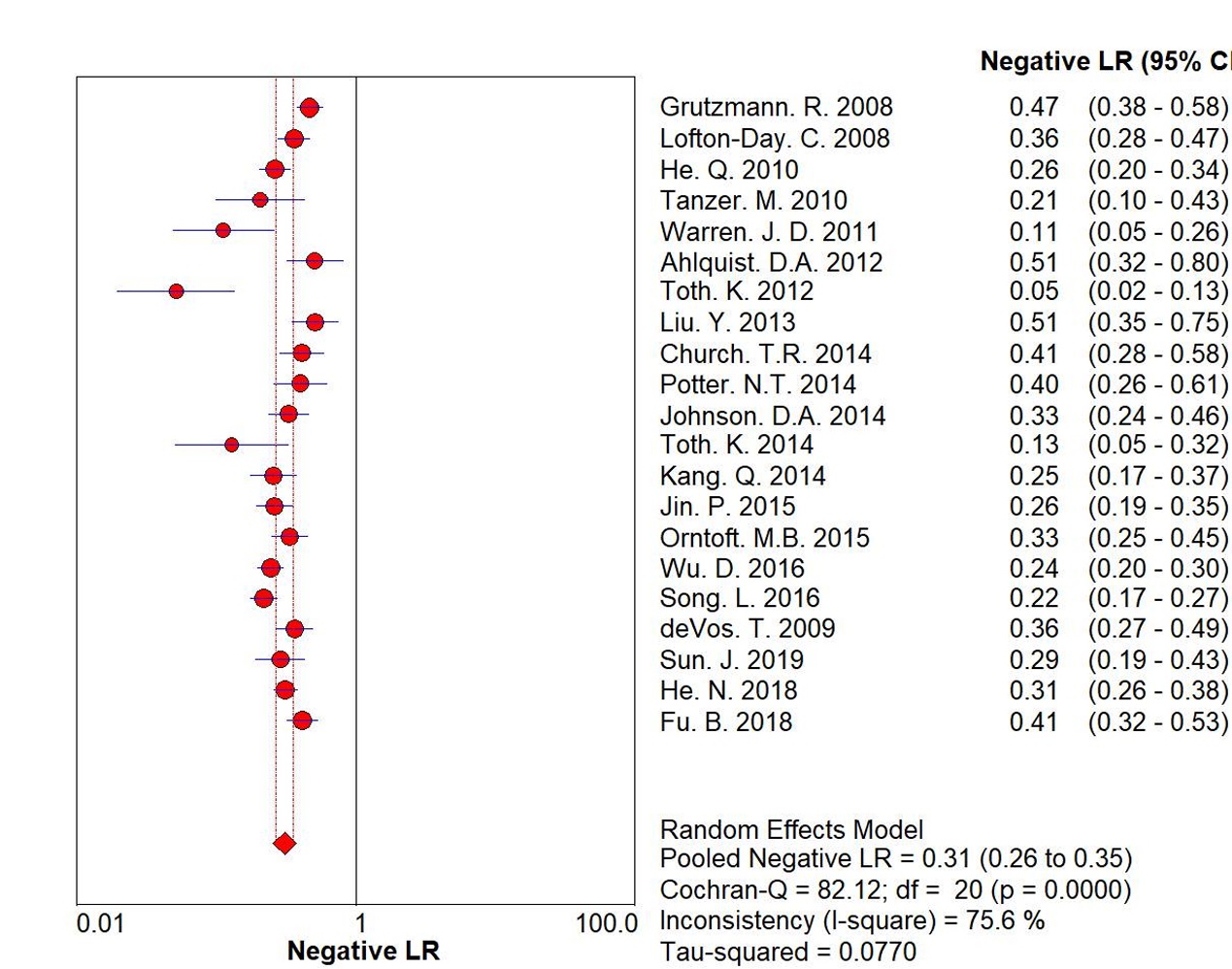


**D**


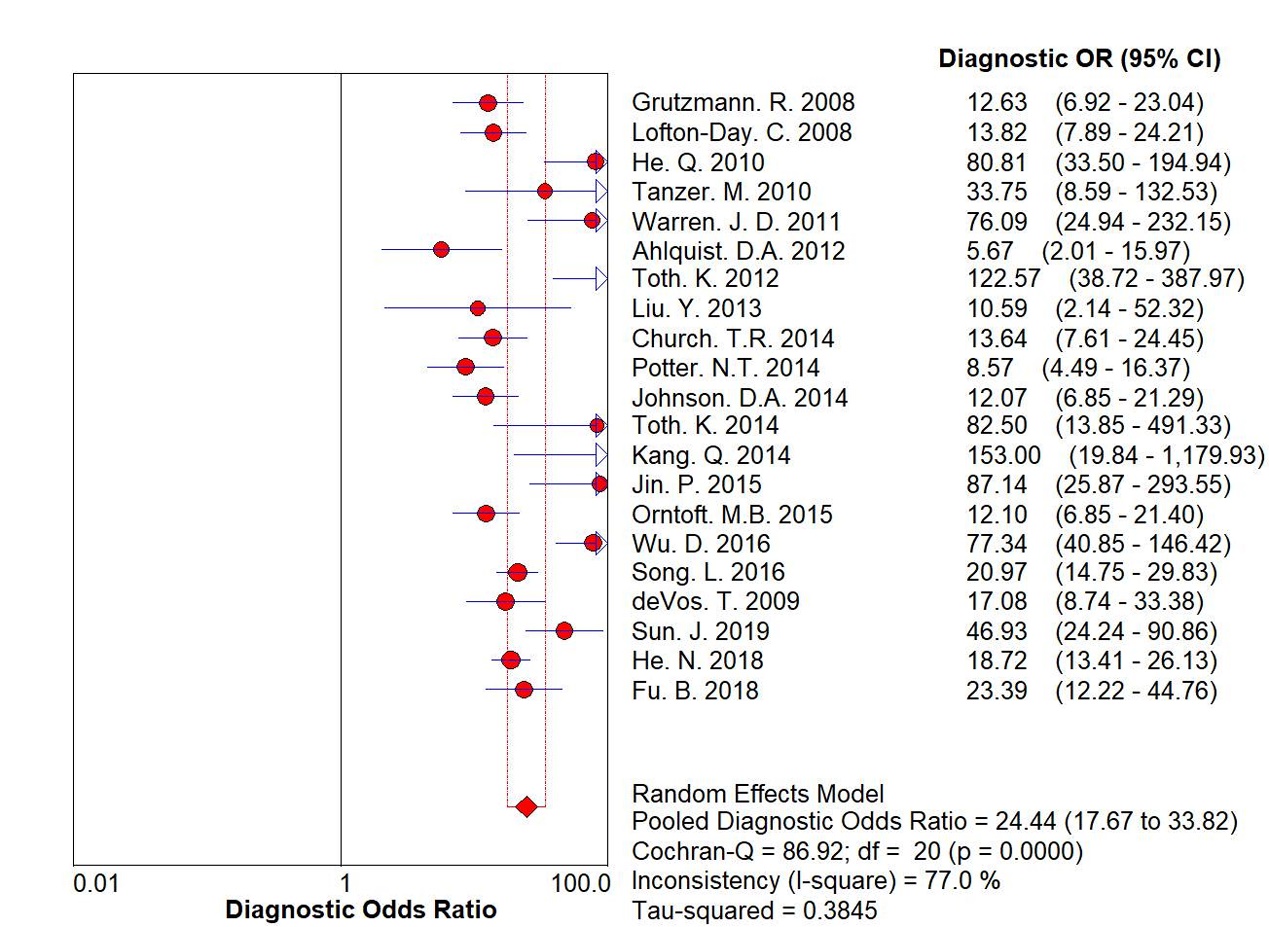


**E**


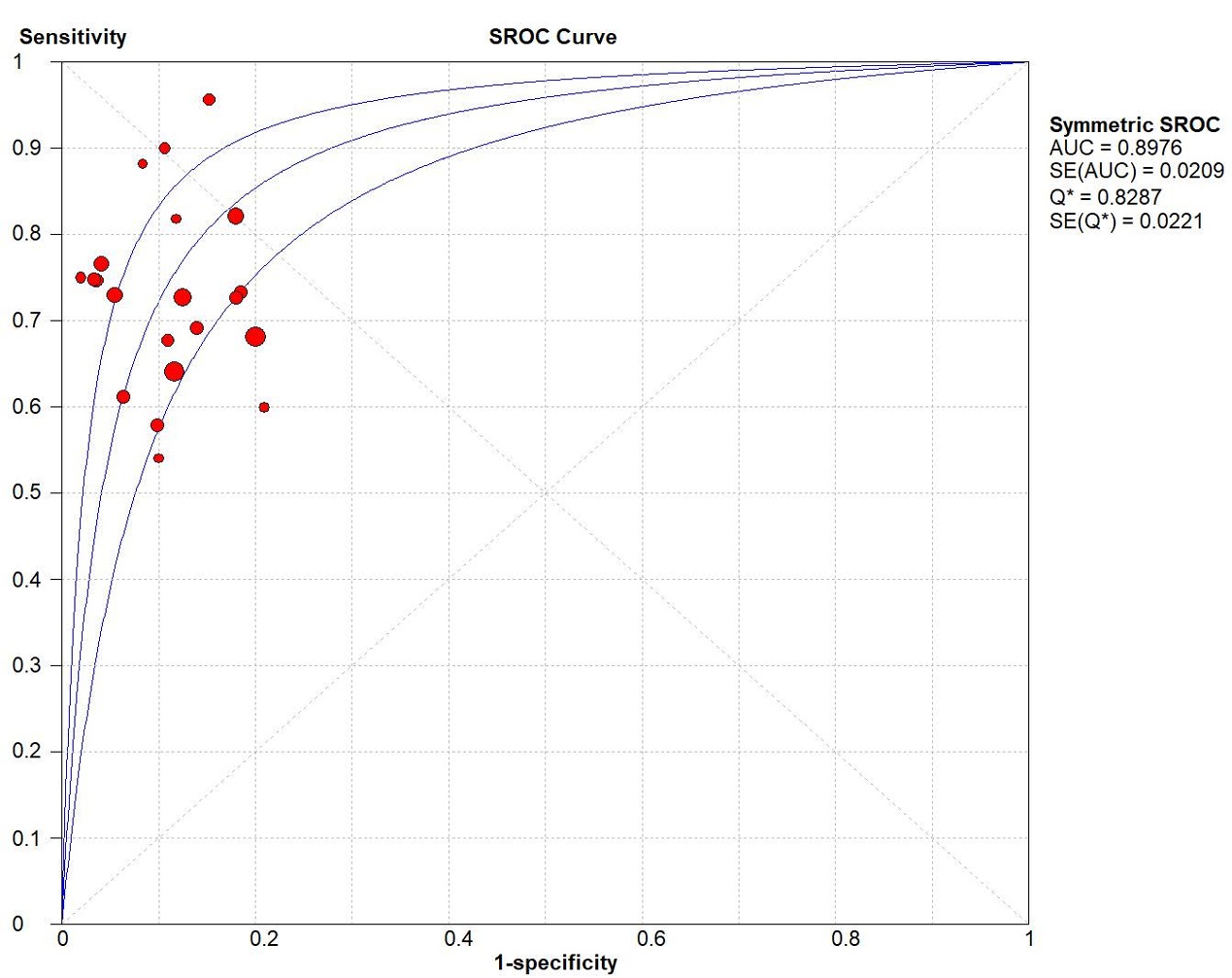


**F**

**F**

**Figure S9.** Forest plots of the diagnostic value for SPET9 methylation in detecting colorectal cancer. (**A**) Sensitivity. (**B**) Specificity. (**C**) positive likelihood ratio. (**D**) negative likelihood ratio. (**E**) Diagnostic odds ratio. (**F**) SROC curve.


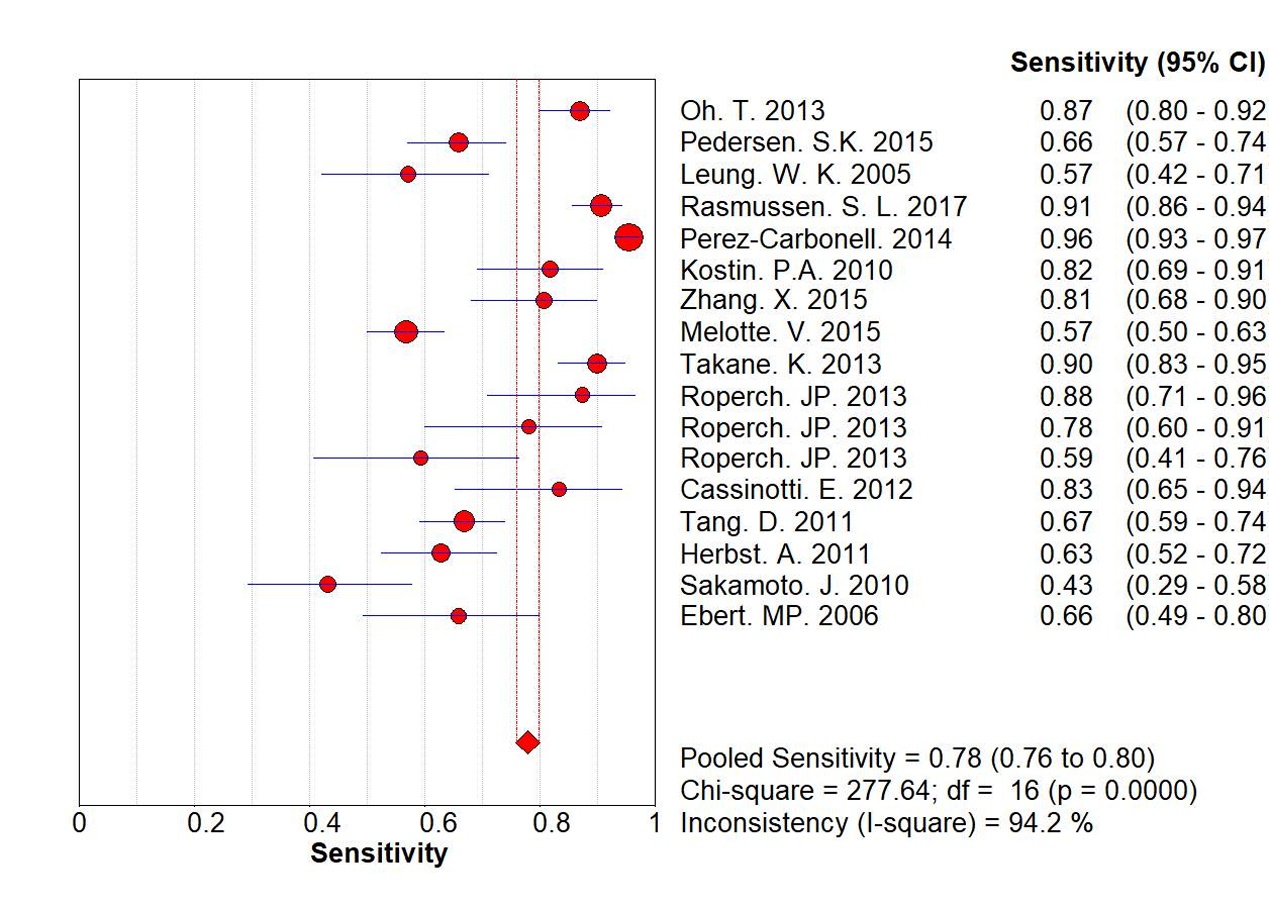

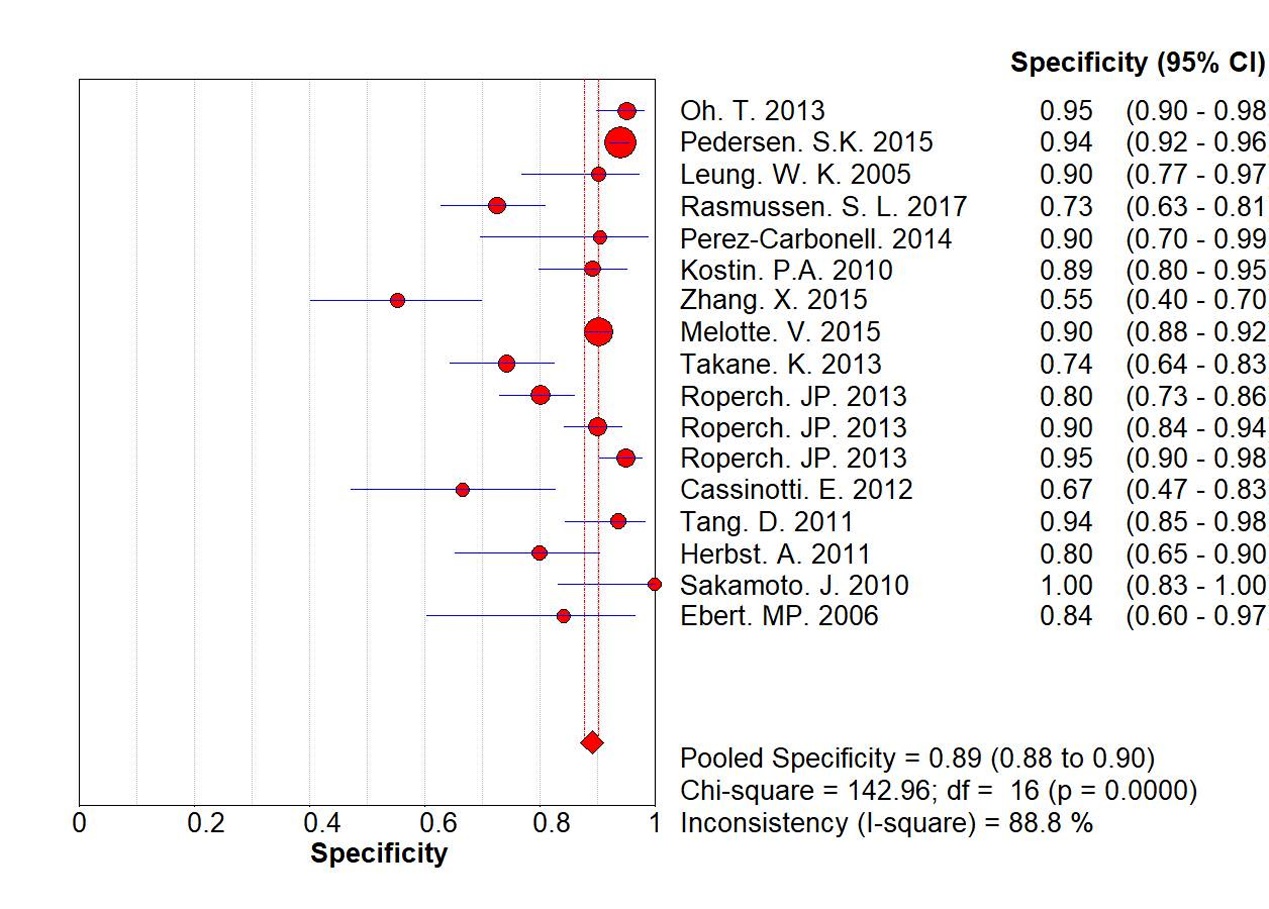

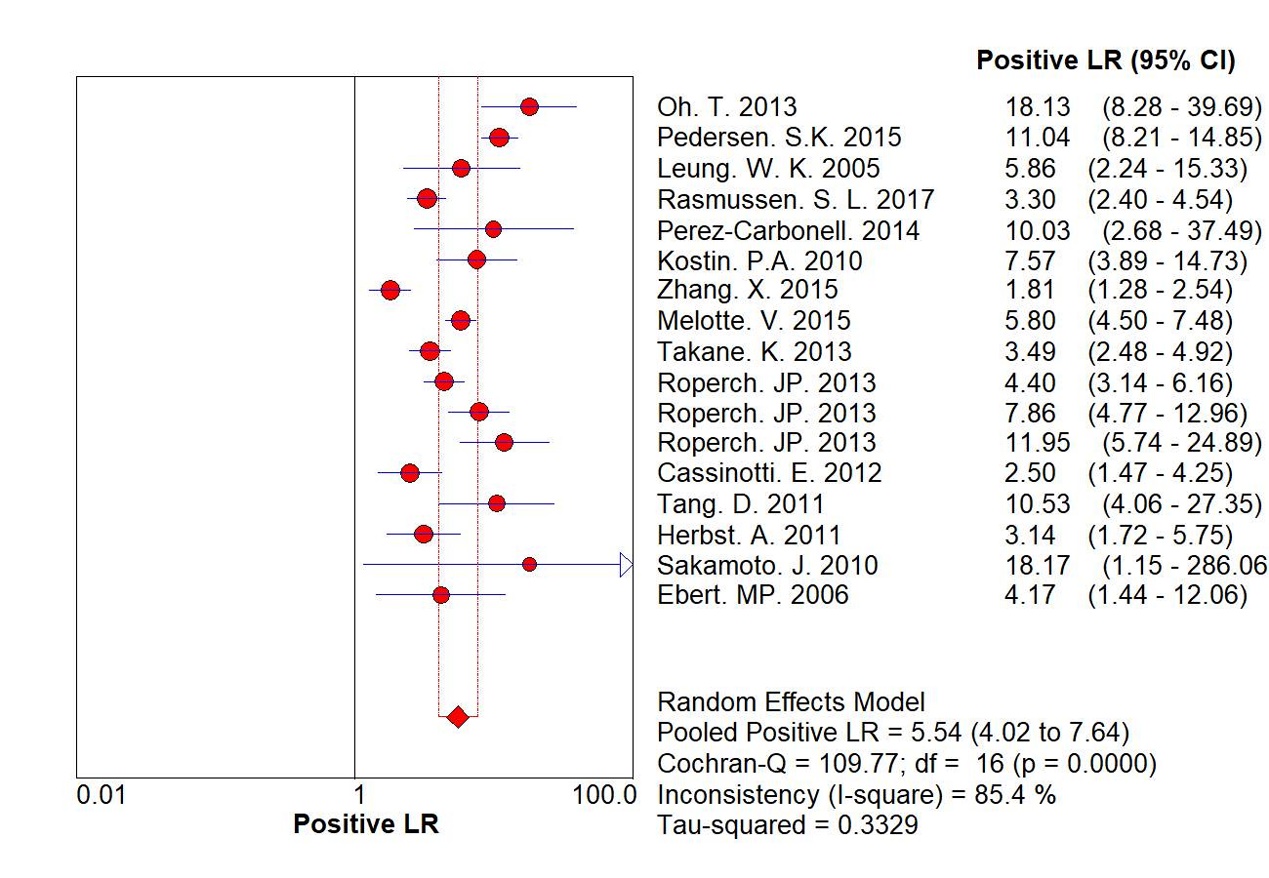

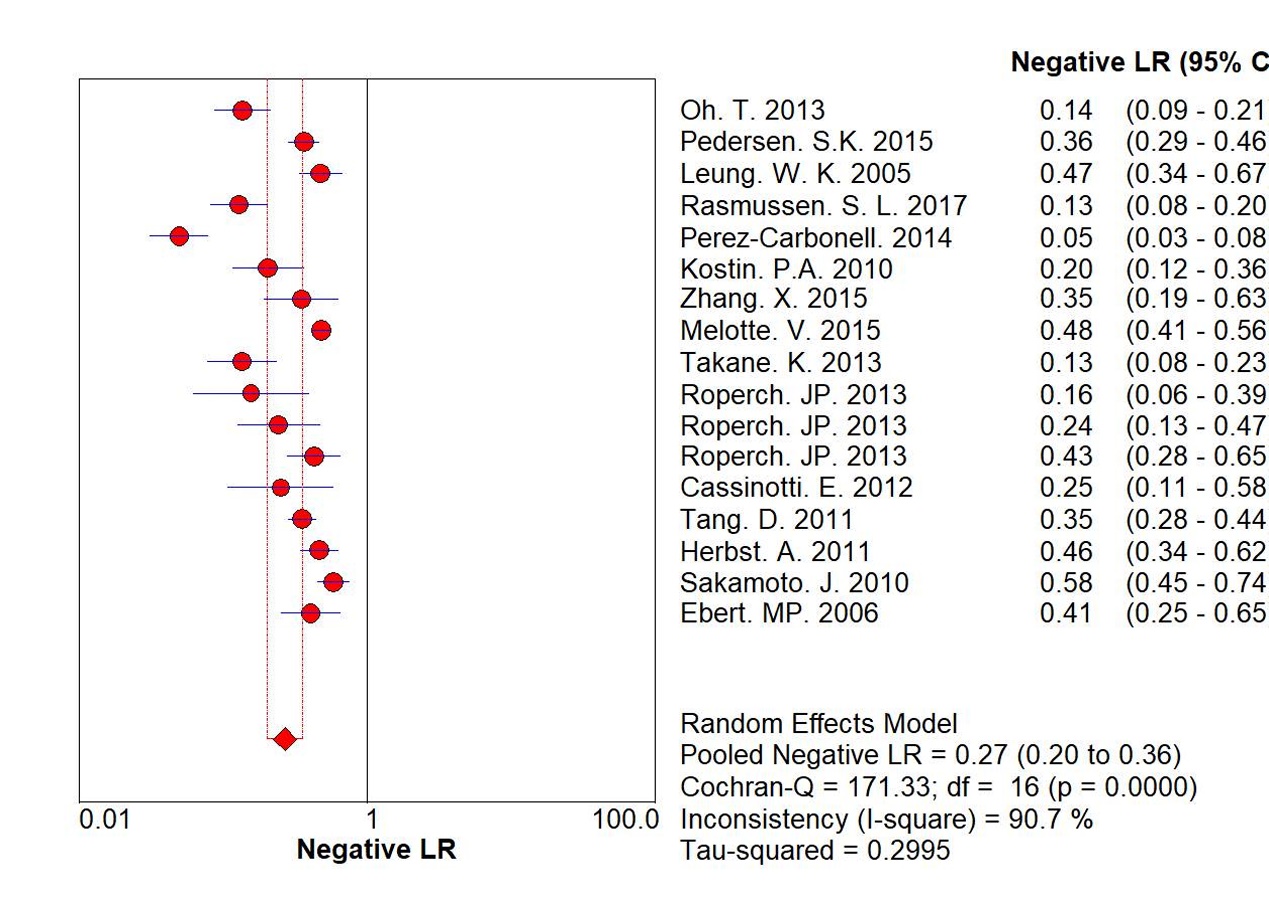

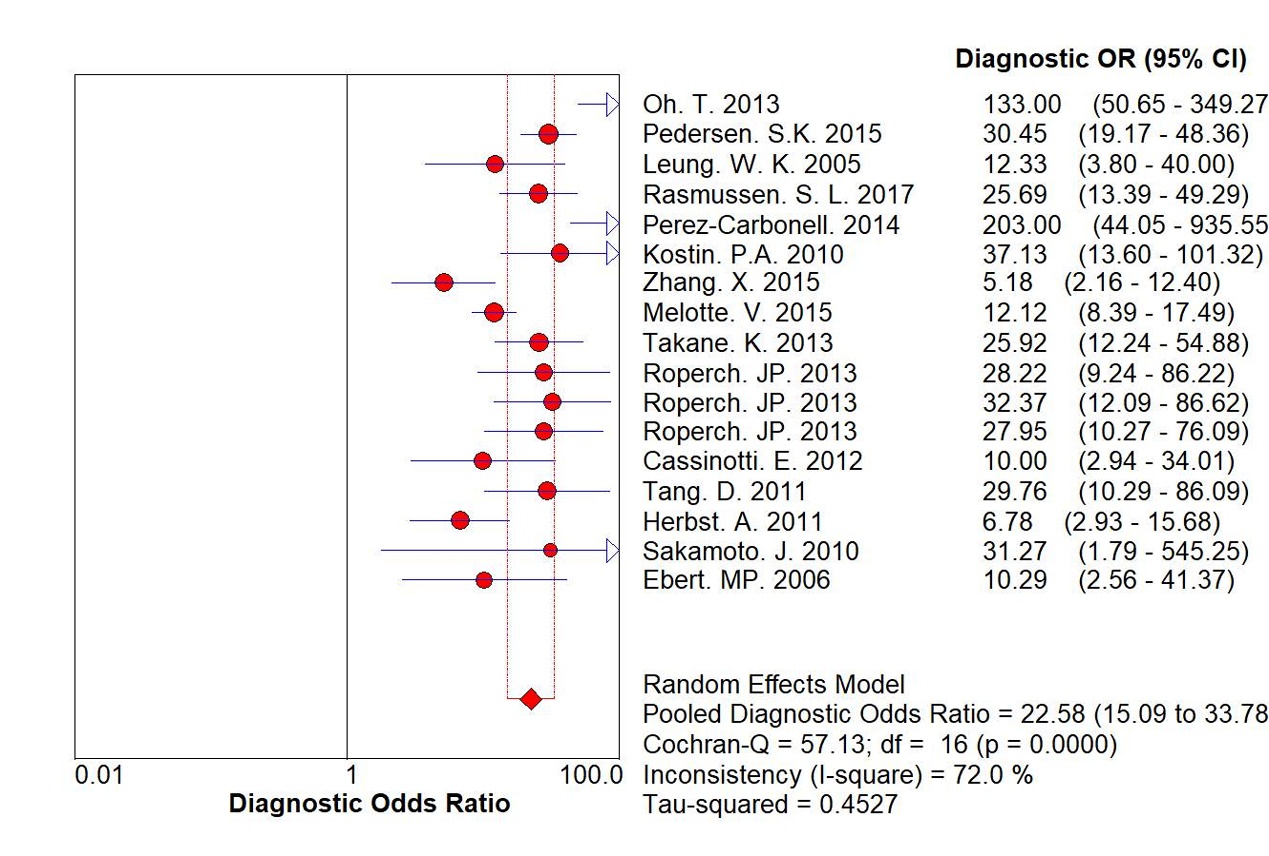


**B**

**A**

**C**

**D**

**E**


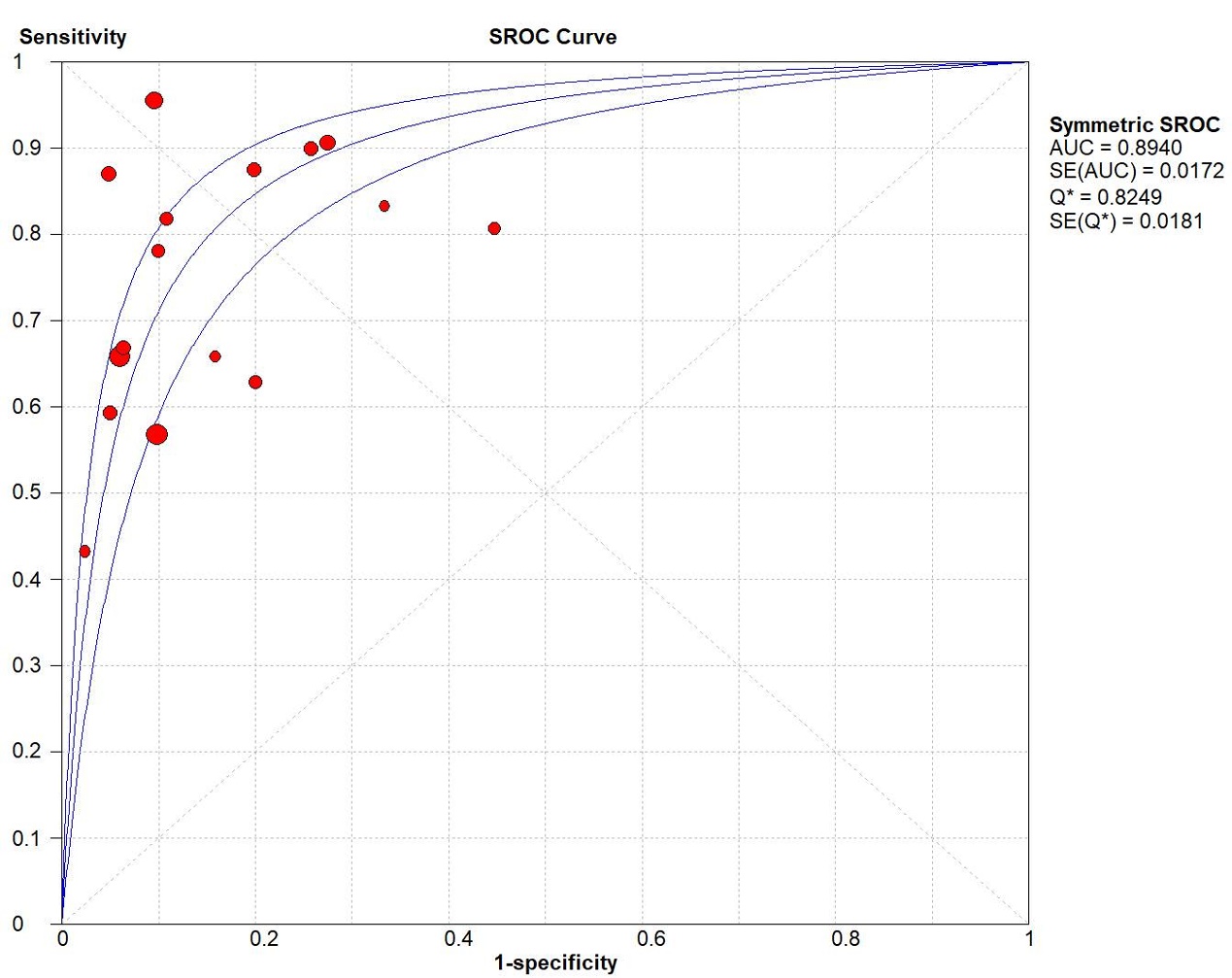


**F**

**Figure S10.** Forest plots of the diagnostic value for cfDNA methylation on other sites (except SEPT9) in detecting colorectal cancer. (**A**) Sensitivity. (**B**) Specificity. (**C**) positive likelihood ratio. (**D**) negative likelihood ratio. (**E**) Diagnostic odds ratio. (**F**) SROC curve.


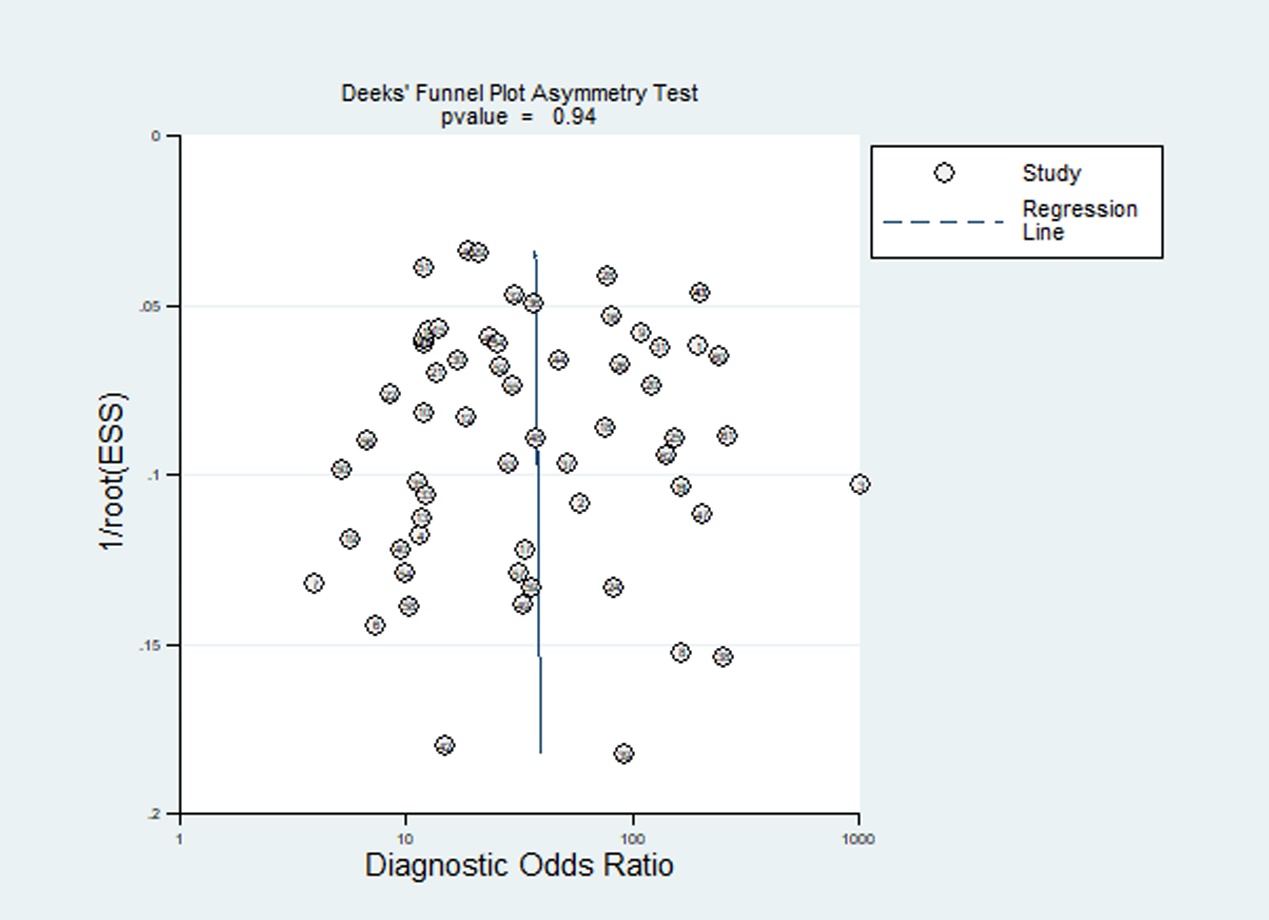


**Figure S11.** Result of Deeks’ funnel test
